# Comparative effect of antihypertensive drugs in improving arterial stiffness in adults with hypertension (RIGIPREV study). A network meta-analysis

**DOI:** 10.3389/fphar.2023.1225795

**Published:** 2023-09-01

**Authors:** Iván Cavero-Redondo, Alicia Saz-Lara, Cristina Lugones-Sánchez, Diana P. Pozuelo-Carrascosa, Leticia Gómez-Sánchez, José Francisco López-Gil, Luis García-Ortiz, Rosa Maria Bruno, Manuel Ángel Gómez-Marcos

**Affiliations:** ^1^ Facultad de Ciencias de la Salud, Universidad Autónoma de Chile, Talca, Chile; ^2^ Health and Social Research Center, Universidad de Castilla- La Mancha, Cuenca, Spain; ^3^ Unidad de Investigación en Atención Primaria de Salamanca (APISAL), Gerencia de Atención Primaria de Salamanca, Gerencia Regional de salud de Castilla y León (SACyL), Salamanca, Spain; ^4^ Instituto de Investigación Biomédica de Salamanca (IBSAL), Salamanca, Spain; ^5^ Red de Investigación en Cronicidad, Atención Primaria y Promoción de la Salud (RICAPPS) (RD21/0016), Barcelona, Spain; ^6^ Navarrabiomed, Hospital Universitario de Navarra (HUN), Universidad Pública de Navarra (UPNA), Pamplona, Spain; ^7^ Department of Environmental Health, Harvard University T. H. Chan School of Public Health, Boston, MA, United States; ^8^ One Health Research Group, Universidad de Las Américas, Quito, Ecuador; ^9^ INSERM U970, Paris Cardiovascular Research Center (PARCC), Université de Paris, Hopital Europeen Georges Pompidou—APHP, Paris, France

**Keywords:** antihypertensive drugs, hypertension, arterial stiffness, pulse wave velocity, augmentation index

## Abstract

**Aims:** To synthesize and evaluate the available scientific evidence on the efficacy of antihypertensive drugs on arterial stiffness in patients with hypertension by using a network meta-analysis approach.

**Methods:** A systematic search of the MEDLINE (via PubMed), Scopus, and Web of Science databases was conducted to identify experimental studies addressing the effect of different antihypertensive drugs on arterial stiffness parameters (pulse wave velocity [PWV] and augmentation index [AIx]) in adults with hypertension. Comparative evaluation of the effect of antihypertensive drugs was performed by conducting a standard pairwise meta-analysis and a network meta-analysis for direct and indirect comparisons between antihypertensive drugs and placebo/other antihypertensive drugs. Analyses were performed including studies of any duration and only studies longer than 6 months length.

**Results:** Seventy-six studies were included in the main analysis and considering only studies longer than 6 months length, thiazide diuretics, ACEIs, ARBs, the ACEI/ARB combination, the ACEI/CCB combination, and the ARB/CCB combination showed a higher effect on reducing PWV, and ACEIs and ARBs on reducing AIx.

**Conclusion:** Our research provides evidence that antihypertensive medications are an effective way to treat arterial stiffness in adults with hypertension. Based on our findings, patients with hypertension who have greater levels of arterial stiffness may benefit from using thiazide diuretics, ACEIs, ARBs, the ACEI/ARB combination, the ACEI/CCB combination, and the ARB/CCB combination.

**Systematic Review Registration:** PROSPERO (CRD42021276360).

## 1 Introduction

Increased blood pressure is one of the major risk factors for cardiovascular disease, affecting more than one-third of the population, accounting for 20.5% of the global burden of cardiovascular disease and the leading cause of death and disability worldwide. (Cooper et al., 2017). Large artery stiffness, a consequence of decreased elastin and increased collagen fibre content in the arterial wall, is an established biomarker of vascular aging; its progression is related to chronological aging but also to cumulative exposure to classical cardiovascular risk factors throughout life. (Laurent et al., 2016). Although increased blood pressure and arterial stiffness are closely related, the temporal relationships between arterial stiffness and blood pressure are not fully established. This relationship is complex and probably bidirectional, with blood pressure stiffening arteries and stiff arteries inducing blood pressure increase. (Nilsson et al., 2018). Longitudinal studies investigating the relationship between arterial stiffness and the development of arterial hypertension have shown that increased arterial stiffness is associated with an increased incidence of hypertension, suggesting that arterial stiffness precedes hypertension. (Mitchell, 2014).

Most patients with hypertension are treated with one or more antihypertensive drugs, (Menéndez et al., 2016), and there are numerous strategies for the treatment of hypertension. (Williams et al., 2018a). The different antihypertensive drugs are effective in preventing the risk of fatal and nonfatal cardiovascular events, and the reduction in these events is attributed to blood pressure reduction *per se* rather than to specific drug properties. (Thomopoulos et al., 2018). However, other studies suggest that the effect of antihypertensive drugs on arterial stiffness differs between groups. (Williams et al., 2018b). Angiotensin-converting enzyme inhibitors (ACEIs), angiotensin II receptor blockers (ARBs), and calcium channel blockers (CCBs) have been shown to decrease arterial stiffness, promote vascular remodelling and improve endothelial function. (Cameron et al., 2016). Other studies suggest that aldosterone is the causative agent of increased arterial stiffness in hypertension. (Seccia et al., 2017; Srinivasa et al., 2023). Drugs such as spironolactone and eplenorone, as aldosterone blockers, reduce arterial stiffness levels in patients with hypertension (Davies et al., 2005; Kalizki et al., 2017) independently of blood pressure levels. (Aryal et al., 2021). However, the medical approach to choosing an antihypertensive drug is mainly based on blood pressure lowering ability, individual patient needs and potential side effects, (Williams et al., 2018b), without considering the effect of each drug on arterial stiffness, even though the evidence suggests that arterial stiffness is an independent risk factor for cardiovascular disease morbidity and mortality. (Williams et al., 2018b).

The efficacy of antihypertensive treatments in arterial stiffness has been meta-analysed separately for different blood pressure-lowering drug classes, such as ACEIs, (Shahin et al., 2012; Li et al., 2020), ARBs (Yen et al., 2014; Peng et al., 2015) or beta-blockers, (Kuyper and Khan, 2014), and using traditional meta-analysis methodology, (Ong et al., 2011; Chen et al., 2015), suggesting that not all antihypertensive drugs may be equally effective in improving arterial stiffness for the same blood pressure reduction. However, this body of evidence does not assist the clinician in making the best choice of antihypertensive drug for the patient in terms of both blood pressure improvement and arterial stiffness reduction. The network meta-analysis (NMA) approach allows estimating the relative effects of different treatments based on the data reported by all available studies and through direct and indirect comparisons, which makes it possible to determine the effects of various treatments in a more comprehensive way. In this article, we aim to synthesize and evaluate the available scientific evidence on the efficacy of antihypertensive drugs on arterial stiffness in patients with hypertension by using an NMA approach.

## 2 Methods

This NMA followed the Preferred Reporting Items for Systematic Review incorporating Network Meta-analysis (PRISMA-NMA) (Hutton et al., 2016) and the Cochrane Collaboration Handbook. (Higgins and Green, 2011). In addition, the protocol for this network meta-analysis has been registered in PROSPERO (CRD42021276360) and published elsewhere. (Cavero-Redondo et al., 2021).

### 2.1 Search methods for study identification electronic search

The literature search was conducted through the MEDLINE, Scopus, Cochrane Central Register of Controlled Trials, Cochrane Database of Systematic Reviews, and Web of Science databases. The above searches were supplemented by manual searches of published or ongoing randomized controlled trials (RCTs) in international trial registries (ClinicalTrials.gov) and on drug approval agency websites. Prior to the final analyses, the searches were repeated only to include all current and potential studies.

To perform the literature search, search strategies were performed by antihypertensive drug groups in combination with the following search terms applying Boolean operators (Supplementary Table S1).

### 2.2 Inclusion/exclusion criteria

Type of Studies: RCTs were included without language restrictions. Type of Participants: Studies evaluating the effect of different antihypertensive drugs on the reduction of arterial stiffness in hypertensive adults with a primary diagnosis of hypertension according to the diagnostic criteria of the International Classification of Diseases (ICD-11) (>18 years of age and of both genders) were selected. If two or more studies provided data on the same sample, the one that presented the most detailed results or provided the largest sample size was chosen. Types of Intervention: Studies using any of the different drugs in the antihypertensive groups as an intervention (Supplementary Table S2), as well as possible drug combinations, were suitable for inclusion, as were studies comparing different types of antihypertensive drugs and examining antihypertensive treatment with or without a control group. However, studies combining antihypertensive drugs with nutritional or lifestyle interventions were excluded when data regarding the effect of antihypertensive drug interventions on arterial stiffness could not be extracted separately. Reductions in different arterial stiffness parameters were analysed as primary outcomes: pulse wave velocity (PWV), augmentation index (AIx), and cardio-ankle vascular index (CAVI).

Indeed, since PWV (and similar approaches, such as CAVI) is a measure of large artery stiffness, whereas AIx is an integrated measure of arterial stiffness and wave reflection, these two measures are analysed separately.

### 2.3 Assessing the risk of bias in the included studies

Based on the recommendations of the Cochrane Collaboration Handbook, two authors independently conducted the risk of bias assessment. (Higgins and Green, 2011). Disagreements were resolved by consensus or with the intervention of a third researcher.

The risk of bias of RCTs was assessed using the Cochrane Collaboration’s tool for assessing risk of bias (RoB2). (Sterne et al., 2019).

### 2.4 Grading the quality of evidence

We used the Grading of Recommendations, Assessment, Development and Evaluation (GRADE) tool to assess the evidence quality and provide recommendations. (Guyatt et al., 2011).

### 2.5 Synthesis of data

We qualitatively summarize the included RCTs in an *ad hoc* table describing direct and indirect comparisons.

The present NMA was conducted as follows: we assessed the strength of the available evidence using a network geometry graph to display the evidence in the network for arterial stiffness (PWV and AIx). In addition, the network geometry graph to show the evidence in the network for arterial stiffness (PWV and AIx) was performed including only studies longer than 6 months length. In this graph, the size of the nodes was proportional to the number of participants in trials who received the intervention specified in the node, and the thickness of the continuous line connecting nodes was proportional to the number of trials directly comparing the two treatments. (Salanti et al., 2011).

Comparative evaluation of the intervention effect on arterial stiffness (PWV and AIx) was performed by conducting a random effects pairwise meta-analysis and a frequentist NMA for comparisons between interventions and controls. Cohen *d* values was calculated, as an estimate of effect size (ES). In addition, these analyses were performed by including only studies longer than 6 months length. We assessed heterogeneity using the *I* ^2^ statistic, (Higgins and Thompson, 2002), ranging from 0% to 100%. Based on the values of *I* ^2^, we categorized heterogeneity as not important (0%–30%), moderate (30%–60%), substantial (60%–75%), or considerable (75%–100%). We also considered the corresponding *p* values. Furthermore, the size and clinical relevance of heterogeneity was determined by the *τ* ^2^ statistic.

Sensitivity analysis was conducted to evaluate the robustness of the pooled estimates, and a reanalysis was conducted by eliminating one study at a time.

Subgroup analyses were conducted based on the type of PWV (central, peripheral, or mixed PWV) and on the type of population (population with exclusively hypertension *versus* patients with hypertension and other pathologies).

Random-effects meta-regression analyses were used to analyse whether mean age, percentage of women, duration of treatment and antihypertensive drug systolic and diastolic blood pressure reduction changed the effect of antihypertensives drugs on arterial stiffness (PWV and AIx).

The probability that each intervention is the most effective was presented by rankograms. In addition, for each intervention, we estimated the surface under cumulative ranking (SUCRA). (Salanti et al., 2011). With SUCRA, a value between 0 and 1 is assigned to rank each intervention in the rankogram. A SUCRA value of approximately 1 was the best intervention, and a SUCRA value of approximately 0 was the worst intervention. SUCRA simplifies the information on the effect of each treatment into a single value, and all complex results of network meta-analysis are expressed with a few numbers. The SUCRA result is most meaningful when the difference in preference between consecutive ranks remains the same over the entire rating scale.

Publication bias was tested using Egger’s regression asymmetry test, (Sterne et al., 2001), setting a level of <0.10 to determine whether publication bias might be present.

The analyses were performed using STATA 15 (StataCorp, College Station, TX).

## 3 Results

Seventy-six studies (Supplementary Table S3) addressing antihypertensive drug interventions for the effect on arterial stiffness were identified, which were conducted in 27 countries from the continents of North America, Europe, Asia, South America, and Oceania. These reports were published between 1992 and 2022.

In the included populations, a total of 5413 patients with arterial hypertension were aged between 37.0 and 72.8 years. The duration of treatment with antihypertensive drugs in the studies ranged from 4 to 208 weeks.

### 3.1 Risk of bias and grade

As evaluated by the RoB2 tool, 80.3% of the studies showed some concerns in the risk of bias, with 10.5% and 9.2% studies showing high and low risks of bias in the overall bias, respectively. (Supplementary Figure S1).

When the quality grading of evidence of each pairwise comparison was evaluated for PWV using the GRADE system, 3.1% of the pairwise comparisons were categorized as high, 45.2% as moderate, 31.3% as low and 19.4% as very low (Supplementary Table S4). When the quality grading of evidence of each pairwise comparison was evaluated for AIx, 3.2% of the pairwise comparisons were categorized as high, 35.4% as moderate, 29.0% as low and 32.4% as very low (Supplementary Table S5).

### 3.2 Effect on pulse wave velocity and augmentation index

**FIGURE 1:**
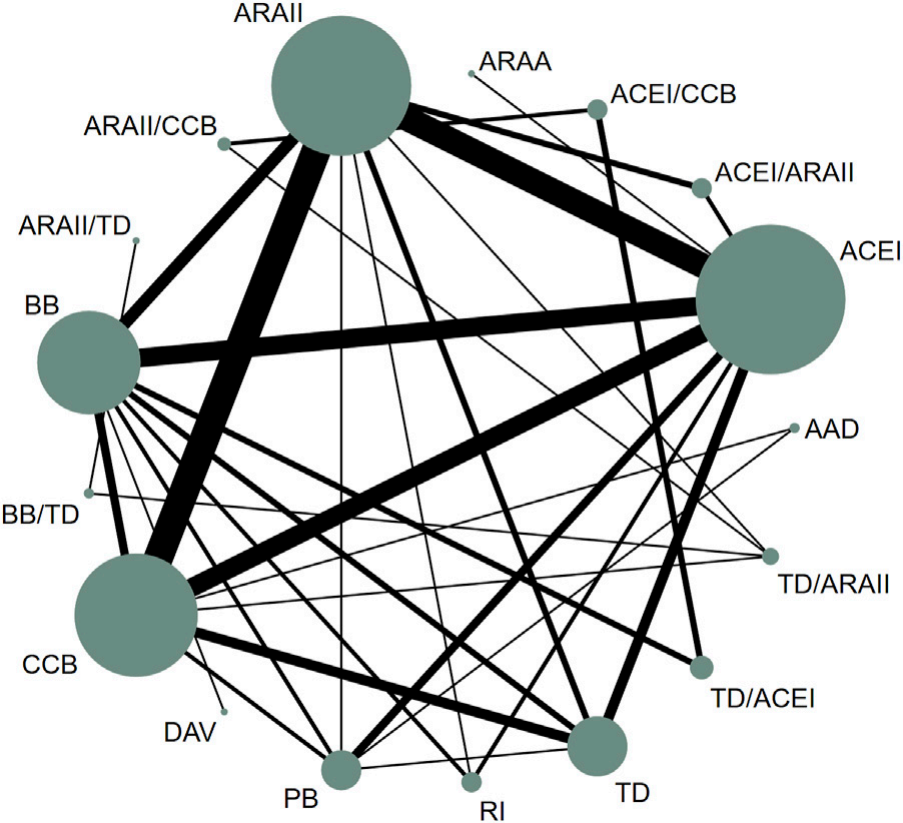
Network of available comparisons between different types of antihypertensive drugs on pulse wave velocity. AAD: antiadosterone diuretics; AARA: Alpha-adrenergic receptor antagonists; ACEI: Angiotensin-converting enzyme inhibitors; ARB: Angiotensin receptor blockers; BB: beta-blockers; CCB: Calcium channel blockers; TD: thiazide diuretics; DAV: direct-acting vasodilators; PB: placebo; RI: renin inhibitors.

Network available comparisons between different types of antihypertensive drugs on PWV are shown in Figure 1. Considering the NMA estimates (lower diagonal) (Table 1), beta-blockers (ES = −0.49; 95% CI: −0.91, −0.07), ACEI (ES = −0.68; 95% CI: −1.08, −0.29), ARB (ES = −0.59; 95% CI: −0.99, −0.19), the ACEI/ARB combination (ES = −1.35; 95% CI: −2.10, −0.60), and the ARB/CCB combination (ES = −0.99; 95% CI: −1.94, −0.04) were effective in reducing PWV. When only studies longer than 6 months length were included, the available network comparisons between the different types of antihypertensive drugs on PWV are shown in Supplementary Figure S2. For these analyses, considering the NMA estimates (lower diagonal) (Table 2), thiazide diuretics (ES = −0.84: 95% CI: −1.55, −0.12), ACEI (ES = −1.05; 95% CI: −1.66, −0.44), ARB (ES = −0.82; 95% CI: −1.48, −0.16), the ACEI/ARB combination (ES = −1.43; 95% CI: −2.49, −0.38), the ACEI/CCB combination (ES = −2.52; 95% CI: −4.75, −0.30), and the ARB/CCB combination (ES = −2.57; 95% CI: −4.96, −0.18) were effective in reducing PWV.

**TABLE 1:** Pooled mean differences of different types of antihypertensive drugs on pulse wave velocity. Upper right triangle gives the pooled mean differences from pairwise comparisons (column intervention relative to row), lower left triangle pooled mean differences from the network meta-analysis (row intervention relative to column). * Values *p* < 0.05 were considered significant.

PB	−0.48 (−0.98, 0.02) n^1^ = 1/n^2^ = 48	0.07 (−0.38, 0.51) n^1^ = 1/n^2^ = 79	−**0.39 (**−**0.76,** −**0.02)** n^1^ = 1/n^2^ = 115	−**1.15** **(**−**2.13,** −**0.18)** n^1^ = 3/n^2^ = 126	0.01 (−0.47, 0.49) n^1^ = 1/n^2^ = 68	−0.36 (−0.72, 0.01) n^1^ = 2/n^2^ = 85	NA	NA	NA	NA	NA	NA	NA	NA	NA
−**0.49 (**−**0.91,** −**0.07)**	BB	0.09 (−0.30, 0.47) n^1^ = 3/n^2^ = 106	NA	−**0.24 (**−**0.47,** −**0.01)** n^1^ = 8/n^2^ = 281	0.12 (−0.07, 0.30) n^1^ = 6/n^2^ = 456	−0.05 (−0.51, 0.41) n^1^ = 4/n^2^ = 135	−0.46 (−0.93, 0.02) n^1^ = 1/n^2^ = 52	NA	−0.34 (−0.71, 0.03) n^1^ = 1/n^2^ = 114	NA	0.02 (−0.15, 0.20) n^1^ = 3/n^2^ = 1019	NA	NA	NA	NA
−0.42 (−0.86, 0.03)	0.08 (−0.27, 0.43)	TD	NA	−0.30 (−0.65, 0.05) n^1^ = 5/n^2^ = 221	−0.04 (−0.35, 0.28) n^1^ = 3/n^2^ = 153	−0.17 (−0.41, 0.07) n^1^ = 5/n^2^ = 196	NA	NA	NA	NA	NA	NA	NA	NA	NA
−0.50 (−1.21, 0.22)	−0.00 (−0.75, 0.75)	−0.08 (−0.85, 0.69)	AAD	NA	NA	−0.20 (−0.54, 0.14) n^1^ = 1/n^2^ = 139	NA	NA	NA	NA	NA	NA	NA	NA	NA
−**0.68 (**−**1.08,** −**0.29)**	−0.19 (−0.46, 0.08)	−0.27 (−0.59, 0.06)	−0.19 (−0.93, 0.55)	ACEI	−0.06 (−0.48, 0.35) n^1^ = 9/n^2^ = 448	0.20 (−0.04, 0.44) n^1^ = 8/n^2^ = 319	0.03 (−0.39, 0.45) n^1^ = 2/n^2^ = 88	0.15 (−0.35, 0.66) n^1^ = 1/n^2^ = 58	NA	NA	NA	NA	−**1.76 (**−**3.04,** −**0.48)** n^1^ = 2/n^2^ = 45	NA	NA
−**0.59 (**−**0.99,** −**0.19)**	−0.10 (−0.36, 0.17)	−0.17 (−0.50, 0.15)	−0.09 (−0.83, 0.64)	0.09 (−0.14, 0.33)	ARB	**0.30 (0.06, 0.55)** n^1^ = 12/n^2^ = 910	0.50 (−0.03, 1.02) n^1^ = 1/n^2^ = 29	NA	NA	NA	NA	−0.34 (−1.09, 0.41) n^1^ = 1/n^2^ = 39	−1.06 (−2.49, 0.36) n^1^ = 3/n^2^ = 435	NA	NA
−0.30 (−0.69, 0.10)	0.20 (−0.09, 0.48)	0.12 (−0.20, 0.44)	0.20 (−0.52, 0.92	**0.39 (0.14, 0.63)**	**0.29 (0.06, 0.53)**	CCB	NA	NA	NA	NA	NA	−0.33 (−0.68, 0.01) n^1^ = 1/n^2^ = 144	NA	NA	NA
−0.46 (−1.11, 0.19)	0.03 (−0.52, 0.59)	−0.04 (−0.65, 0.57)	0.04 (−0.87, 0.94)	0.22 (−0.32, 0.77)	0.13 (−0.42, 0.68)	−0.16 (−0.74, 0.41)	RI	NA	NA	NA	NA	NA	NA	NA	NA
−0.28 (−1.35, 0.78)	0.21 (−0.82, 1.24)	0.13 (−0.92, 1.18)	0.21 (−1.03, 1.45)	0.40 (−0.60, 1.40)	0.31 (−0.72, 1.33)	0.01 (−1.01, 1.04)	0.18 (−0.96, 1.31)	AARA	NA	NA	NA	NA	NA	NA	NA
−0.96 (−2.03, 0.11)	−0.47 (−1.45, 0.51)	−0.55 (−1.59, 0.50)	−0.47 (−1.70, 0.77)	−0.28 (−1.30, 0.74)	−0.37 (−1.39, 0.65)	−0.67 (−1.69, 0.36)	−0.50 (−1.63, 0.63)	−0.68 (−2.10, 0.75)	DAV	NA	NA	NA	NA	NA	NA
−0.52 (−1.58, 0.54)	−0.03 (−1.04, 0.99)	−0.10 (−1.14, 0.93)	−0.02 (−1.25, 1.20)	0.17 (-0.85, 1.18)	0.07 (-0.94, 1.08)	−0.22 (−1.22, 0.78)	−0.06 (−1.07, 1.19)	−0.23 (−1.66, 1.19)	0.44 (−0.97, 1.86)	BB/TD	NA	−0.49 (−1.31, 0.34) n^1^ = 2/n^2^ = 124	NA	NA	NA
−0.58 (−1.22, 0.07)	−0.09 (−0.59, 0.42)	−0.16 (−0.77, 0.44)	−0.08 (−0.98, 0.82)	0.11 (−0.46, 0.67)	0.01 (−0.55, 0.57)	−0.28 (−0.85, 0.29)	−0.12 (−0.87, 0.63)	−0.29 (−1.44, 0.85)	0.38 (−0.72, 1.49)	−0.06 (−1.14, 1.02)	TD/ACEI	0.14 (−0.48, 0.76) n^1^ = 1/n^2^ = 40	NA	0.35 (−0.07, 0.78) n^1^ = 3/n^2^ = 128	0.04 (−0.58, 0.66) n^1^ = 1/n^2^ = 40
−0.63 (−1.35, 0.08)	−0.23 (−0.96, 0.51)	−0.30 (−1.07, 0.46)	−0.23 (−1.23, 0.78)	−0.04 (−0.77, 0.69)	−0.13 (−0.59, 0.85)	−0.42 (−1.14, 0.29)	−0.26 (−1.15, 0.63)	−0.44 (−1.67, 0.80)	0.24 (−0.99, 1.47)	−0.20 (−0.90, 0.50)	−0.14 (−0.96, 0.67)	TD/ARB	NA	−0.00 (−0.62, 0.62) n^1^ = 1/n^2^ = 40	−0.27 (−0.54, 0.00) n^1^ = 1/n^2^ = 207
−**1.35 (**−**2.10,** −**0.60)**	−**0.86 (**−**1.54,** −**0.18)**	−**0.94 (**−**1.64,** −**0.23)**	−0.86 (−1.83, 0.11)	−**0.67 (**−**1.32, -0.02)**	−**0.76 (**−**1.40,** −**0.13)**	−**1.06 (**−**1.73,** −**0.39)**	−**0.89 (**−**1.73,** −**0.06)**	−1.07 (−2.26, 0.12)	−0.39 (−1.59, 0.81)	−0.83 (−2.02, 0.35)	−0.78 (−1.61, 0.06)	−0.63 (−1.59, 0.32)	ACEI/ARB	NA	NA
−0.80 (−1.69, 0.09)	−0.31 (−1.11, 0.50)	−0.38 (−1.24, 0.48)	−0.30 (−1.39, 0.78)	−0.12 (−0.95, 0.72)	−0.21 (−1.04, 0.62)	−0.50 (−1.33, 0.33)	−0.34 (−1.31, 0.63)	0.52 (−1.81, 0.78)	0.16 (−1.11, 1.43)	−0.28 (−1.41, 0.85)	−0.22 (−0.93, 0.49)	−0.08 (−0.96, 0.80)	0.55 (−0.48, 1.59)	ACEI/CCB	−0.27 (−0.62, 0.08) n^1^ = 2/n^2^ = 128
−**0.99 (**−**1.94,** −**0.04)**	−0.50 (−1.38, 0.39)	−0.57 (−1.50, 0.35)	−0.49 (−1.63, 0.64)	−0.31 (−1.20, 0.59)	−0.40 (−1.29, 0.49)	−0.69 (−1.58, 0.19)	−0.53 (−1.56, 0.50)	−0.71 (−2.05, 0.63)	−0.03 (−1.35, 1.29)	−0.47 (−1.52, 0.58)	−0.41 (−1.28, 0.46)	−0.27 (−1.05, 0.52)	0.36 (−0.73, 1.45)	−0.19 (−0.91, 0.53)	ARB/CCB

n^1^ = trials; n^2^ = subjects.

AAD: antiadosterone diuretics; AARA: Alpha-adrenergic receptor antagonists; ACEI: Angiotensin-converting enzyme inhibitors; ARB: Angiotensin receptor blockers; BB: beta-blockers; CCB: Calcium channel blockers; TD: thiazide diuretics; DAV: direct-acting vasodilators; PB: placebo; RI: renin inhibitors.

**TABLE 2:** Pooled mean differences of different types of antihypertensive drugs on pulse wave velocity including only studies longer than 6 months length. Upper right triangle gives the pooled mean differences from pairwise comparisons (column intervention relative to row), lower left triangle pooled mean differences from the network meta-analysis (row intervention relative to column). * Values *p* < 0.05 were considered significant.

PB	NA	**NA**	NA	**−1.13** **(−2.06, −0.21)** n^1^ = 4/n^2^ = 126	NA	NA	NA	NA	NA	NA	NA	NA	NA
−0.71 (−1.42, 0.00)	BB	**0.05** **(−0.40, 0.50)** **n^1^ = 2/n^2^ = 76**	NA	−0.23 (−0.57, 0.10) n^1^ = 4/n^2^ = 141	0.11 (−0.10, 0.32) n^1^ = 4/n^2^ = 358	−0.09 (−0.54, 0.36) n^1^ = 2/n^2^ = 76	NA	−0.34 (−0.71, 0.03) n^1^ = 1/n^2^ = 114	0.02 (−0.15, 0.20) n^1^ = 3/n^2^ = 731	NA	NA	NA	NA
**−0.84** **(−1.55, −0.12)**	−0.13 (−0.62, 0.36)	TD	NA	−0.40 (−0.92, 0.13) n^1^ = 3/n^2^ = 116	−0.00 (−0.45, 0.45) n^1^ = 2/n^2^ = 76	−0.15 (−0.60, 0.30) n^1^ = 2/n^2^ = 76	NA	NA	NA	NA	NA	NA	NA
−0.99 (−2.24, 0.26)	−0.28 (−1.43, 0.87)	−0.15 (−1.31, 1.00)	AAD	NA	NA	0.20 (−0.14, 0.54) n^1^ = 1/n^2^ = 139	NA	NA	NA	NA	NA	NA	NA
**−1.05** **(−1.66, −0.44)**	−0.34 (−0.74, 0.06)	−0.21 (−0.65, 0.23)	−0.06 (−1.18, 1.07)	ACEI	−0.01 (−0.56, 0.54) n^1^ = 9/n^2^ = 436	0.13 (−0.16, 0.43) n^1^ = 4/n^2^ = 132	NA	NA	NA	NA	**−1.15** **(−2.13, −0.18)** n^1^ = 1/n^2^ = 21	NA	NA
**−0.82** **(−1.48, −0.16)**	−0.11 (−0.50, 0.27)	0.02 (−0.43, 0.46)	0.17 (−0.95, 1.29)	0.23 (−0.09, 0.54)	ARB	0.01 (−0.11, 0.13) n^1^ = 7/n^2^ = 686	0.50 (−0.03, 1.02) n^1^ = 1/n^2^ = 29	NA	NA	NA	−0.38 (−1.30, 0.55) n^1^ = 2/n^2^ = 411	NA	NA
−0.61 (−1.26, 0.04)	0.10 (−0.33, 0.53)	0.23 (−0.22, 0.67)	0.38 (−0.68, 1.44)	**0.44** **(0.07, 0.80)**	0.21 (**−**0.13, 0.55)	CCB	NA	NA	NA	−0.33 (−0.68, 0.01) n^1^ = 1/n^2^ = 131	NA	NA	NA
−0.33 (−1.63, 0.97)	0.38 (−0.80, 1.56)	0.51 (−0.69, 1.71)	0.66 (−0.92, 2.24)	0.72 (−0.44, 1.88)	0.49 (**−**0.63, 1.61)	0.28 (−1.45, 0.89)	RI	NA	NA	NA	NA	NA	NA
−1.18 (−2.47, 0.11)	−0.47 (−1.55, 0.61)	−0.34 (−1.52, 0.84)	−0.19 (−1.76, 1.39)	−0.13 (−1.28, 1.02)	−0.36 (−1.50, 0.79)	−0.57 (−1.73, 0.59)	−0.85 (−2.44, 0.75)	DAV	NA	NA	NA	NA	NA
−0.74 (−1.66, 0.18)	−0.03 (−0.62, 0.56)	0.10 (−0.66, 0.86)	0.25 (−1.04, 1.54)	0.31 (−0.40, 1.02)	0.08 (−0.62, 0.78)	−0.13 (−0.85, 0.60)	−0.41 (−1.72, 0.91)	0.44 (−0.79, 1.567	TD/ACEI	NA	NA	**−0.68** **(−1.17, −0.19)** n^1^ = 1/n^2^ = 54	NA
−1.17 (−2.42, 0.08)	−0.46 (−1.61, 0.69)	−0.33 (−1.49, 0.82)	−0.18 (−1.69, 1.33)	−0.12 (−1.25, 1.00)	−0.35 (−1.47, 0.77)	−0.56 (−1.63, 0.51)	−0.84 (−2.42, 0.74)	0.01 (−1.57, 1.58)	0.43 (−1.72, 0.86)	TD/ARB	NA	−0.00 (−0.62, 0.62) n^1^ = 1/n^2^ = 207	NA
**−1.43** **(−2.49, −0.38)**	−0.73 (−1.64, 0.19)	-0.60 (-1.53, 0.34)	−0.45 (−1.84, 0.95)	−0.39 (−1.26, 0.48)	−0.61 (−1.45, 0.22)	−0.83 (−1.72, 0.07)	−1.10 (−2.50, 0.29)	−0.26 (−1.67, 1.16)	−0.70 (−1.78, 0.39)	−0.27 (−1.66, 1.13)	ACEI/ARB	NA	NA
**−2.52** **(−4.75, −0.30)**	−1.82 (−3.93, 0.29)	−1.69 (−3.85, 0.48)	−1.53 (−3.94, 0.87)	−1.48 (−3.63, 0.67)	−1.70 (−3.85, 0.44)	−1.91 (−4.07, 0.24)	−2.19 (−4.61, 0.22)	−1.35 (−3.72, 1.02)	−1.79 (−3.82, 0.24)	−1.35 (−3.76, 1.05)	−1.09 (−3.39, 1.21)	ACEI/CCB	−0.27 (−0.62, 0.08) n^1^ = 2/n^2^ = 128
**−2.57** **(−4.96, −0.18)**	−1.87 (−4.15, 0.42)	−1.74 (−4.07, 0.60)	−1.58 (−4.14, 0.97)	−1.53 (−3.84, 0.79)	−1.75 (−4.07, 0.56)	−1.96 (−4.29, 0.36)	−2.24 (−4.81, 0.33)	−1.40 (−3.92, 1.13)	−1.84 (−4.04, 0.37)	−1.40 (−3.96, 1.15)	−1.14 (−3.60, 1.32)	−0.05 (−0.92, 0.82)	ARB/CCB

n^1^ = trials; n^2^ = subjects.

AAD: antialdosterone diuretics; ACEI: Angiotensin-converting enzyme inhibitors; ARB: Angiotensin receptor blockers; BB: beta-blockers; CCB: Calcium channel blockers; DAV: direct-acting vasodilators; PB: placebo; RI: renin inhibitors; TD: thiazide diuretics.

**FIGURE 2:**
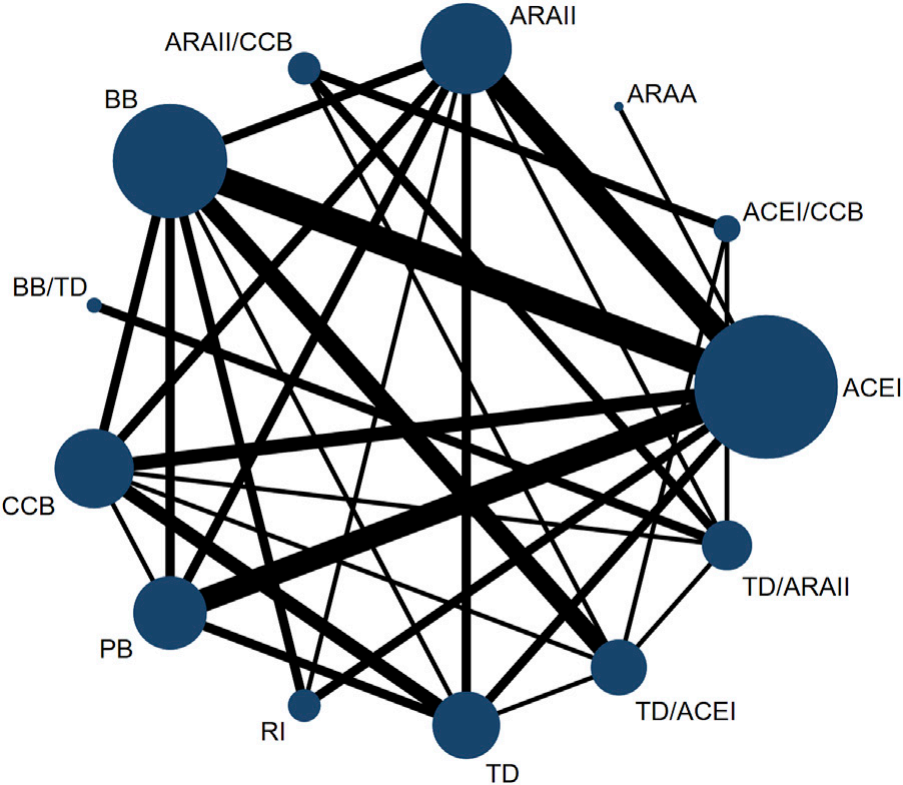
Network of available comparisons between different types of antihypertensive drugs on augmentation index. AARA: Alpha-adrenergic receptor antagonists; ACEI: Angiotensin-converting enzyme inhibitors; ARB: Angiotensin receptor blockers; BB: beta-blockers; CCB: Calcium channel blockers; TD: thiazide diuretics; PB: placebo; RI: renin inhibitors.

Furthermore, network available comparisons between different types of antihypertensive drugs on AIx are shown in Figure 2. Considering the NMA estimates (lower diagonal) (Table 3), ACEI (ES = −0.83; 95% CI: −1.27, −0.38), ARB (ES = −0.56; 95% CI: −1.05, −0.08), CCB (ES = −0.63; 95% CI: −1.17, −0.09), renin inhibitor (ES = −0.73; 95% CI: −1.44, −0.02), the thiazide diuretic/ACEI combination (ES = −0.72; 95% CI: −1.42, −0.02) and the ARB/CCB combination (ES = −0.81; 95% CI: −1.62, −0.01) were effective in reducing AIx. When only studies longer than 6 months length were included, the available network comparisons between the different types of antihypertensive drugs on AIx are shown in Supplementary Figure S3. For these analyses, considering the NMA estimates (lower diagonal) (Table 4), ACEI (ES = −1.65; 95% CI: −2.52, −0.88), and ARB (ES = −1.21; 95% CI: −2.30, −0.12) were effective in reducing AIx.

**TABLE 3:** Pooled mean differences of different types of antihypertensive drugs on augmentation index. Upper right triangle gives the pooled mean differences from pairwise comparisons (column intervention relative to row), lower left triangle pooled mean differences from the network meta-analysis (row intervention relative to column). * Values *p* < 0.05 were considered significant.

PB	**0.73** **(0.22, 1.24)** n^1^ = 1/n^2^ = 48	−0.24 (−0.60, 0.12) n^1^ = 2/n^2^ = 119	**−1.32** **(−2.42, −0.22)** n^1^ = 4/n^2^ = 237	−1.00 (−2.61, 0.60) n^1^ = 2/n^2^ = 108	−0.25 (−0.73, 0.23) n^1^ = 1/n^2^ = 68	NA	NA	NA	NA	NA	NA	NA
−0.03 (−0.52, 0.47)	BB	−0.47 (−1.20, 0.26) n^1^ = 1/n^2^ = 30	−0.28 (−0.87, 0.30) n^1^ = 5/n^2^ = 207	−0.36 (−0.73, 0.00) n^1^ = 2/n^2^ = 338	**−1.20** **(−1.75, −0.66)** n^1^ = 2/n^2^ = 66	0.35 (−0.34, 1.03) n^1^ = 1/n^2^ = 52	NA	NA	**−0.39** **(−0.58, −0.19)** n^1^ = 4/n^2^ = 1071	NA	NA	NA
−0.36 (−0.89, 0.16)	−0.33 (−0.85, 0.18)	TD	**−0.37** **(−0.72, -0.02)** n^1^ = 2/n^2^ = 129	−0.83 (−2.51, 0.86) n^1^ = 2/n^2^ = 117	0.09 (−0.65, 0.46) n^1^ = 3/n^2^ = 132	NA	NA	NA	**−1.02** **(−1.58, −0.46)** n^1^ = 1/n^2^ = 28	NA	NA	NA
**−0.83** **(−1.27, −0.38)**	**−0.80** **(−1.18, −0.42)**	−0.47 (−0.95, 0.02)	ACEI	0.39 (−0.19, 0.96) n^1^ = 3/n^2^ = 234	−0.15 (−0.59, 0.28) n^1^ = 3/n^2^ = 88	−0.14 (−0.49, 0.21) n^1^ = 2/n^2^ = 103	0.21 (−0.30, 0.71) n^1^ = 1/n^2^ = 58	NA	NA	NA	NA	NA
**−0.56** **(−1.05, −0.08)**	**−0.54** **(−0.96, −0.11)**	−0.20 (−0.70, 0.30)	0.26 (−0.12, 0.65)	ARB	−0.02 (−0.22, 0.26) n^1^ = 2/n^2^ = 266	−0.29 (−1.02, 0.44) n^1^ = 1/n^2^ = 29	NA	NA	NA	−0.45 (−0.31, 1.21) n^1^ = 1/n^2^ = 39	NA	NA
**−0.63** **(−1.17, −0.09)**	**−0.60** **(−1.09, -0.12)**	−0.27 (−0.80, 0.26)	0.20 (−0.27, 0.66)	−0.07 (−0.54, 0.41)	CCB	NA	NA	NA	−**1.33** **(**−**1.91,** −**0.75)** n^1^ = 1/n^2^ = 28	0.06 (−0.28, 0.40) n^1^ = 1/n^2^ = 144	NA	NA
**−0.73** **(−1.44, −0.02)**	**−0.71** **(−1.32, −0.09)**	−0.37 (−1.10, 0.36)	0.10 (−0.51, 0.70)	−0.17 (−0.81, 0.47)	−0.10 (−0.81, 0.61)	RI	NA	NA	NA	NA	NA	NA
−0.73 (−2.08, 0.63)	−0.70 (−2.03, 0.63)	−0.37 (−1.73, 1.00)	0.10 (−1.18, 1.38)	−0.16 (−1.50, 1.17)	−0.10 (−1.46, 1.26)	−0.00 (−1.41, 1.42)	AARA	NA	NA	NA	NA	NA
−0.19 (−1.44, 1.06)	−0.16 (−1.36, 1.04)	0.17 (−1.07, 1.42)	0.54 (−0.57, 1.85)	0.38 (−0.83, 1.58)	0.44 (−0.76, 1.64)	0.54 (−0.77, 1.86)	0.54 (−1.22, 2.30)	BB/TD	NA	**−0.90** **(−1.62, −0.18)** n^1^ = 2/n^2^ = 124	NA	NA
**−0.72** **(−1.42, −0.02)**	**−0.70** **(−1.25, −0.14)**	−0.36 (−1.05, 0.33)	0.11 (−0.52, 0.73)	−0.16 (−0.80, 0.48)	−0.09 (−0.75, 0.57)	0.01 (−0.79, 0.81)	0.01 (−1.42, 1.43)	−0.53 (−1.72, 0.65)	TD/ACEI	0.02 (−0.60, 0.64) n^1^ = 1 / n^2^ = 40	−0.14 (−0.76, 0.48) n^1^ = 1/n^2^ = 40	0.01 (−0.61, 0.63) n^1^ = 1/n^2^ = 40
−0.70 (−1.54, 0.13)	−0.68 (−1.44, 0.08)	−0.34 (−1.17, 0.48)	0.12 (−0.65, 0.90)	−0.14 (−0.91, 0.62)	−0.07 (−0.83, 0.68)	0.03 (−0.90, 0.96)	0.02 (−1.47, 1.52)	−0.52 (−1.45, 0.41)	0.02 (−0.71, 0.74)	TD/ARB	−0.14 (−0.76, 0.48) n^1^ = 1/n^2^ = 40	−0.16 (−0.34, 0.03) n^1^ = 2/n^2^ = 247
−0.94 (−1.97, 0.10)	−0.91 (−1.87, 0.05)	−0.58 (−1.61, 0.45)	−0.11 (−1.10, 0.88)	−0.38 (−0.61, 1.36)	−0.31 (−1.30, 0.68)	−0.21 (−1.32, 0.90)	−0.21 (−1.83, 1.40)	−0.75 (−1.99, 0.49)	−0.22 (−1.10, 0.66)	−0.23 (−1.05, 0.59)	ACEI/CCB	−0.14 (−0.57, 0.30) n^1^ = 2/n^2^ = 128
**−0.98** **(−1.95, −0.01)**	**−0.95** **(−1.85, −0.06)**	−0.62 (−1.58, 0.34)	−0.15 (−1.07, 0.77)	−0.42 (−1.33, 0.50)	−0.35 (−1.26, 0.57)	−0.25 (−1.30, 0.80)	−0.25 (−1.83, 1.32)	−0.79 (−1.96, 0.37)	−0.26 (−1.07, 0.56)	−0.27 (−0.97, 0.42)	−0.04 (−0.78, 0.70)	ARB/CCB

n^1^ = trials; n^2^ = subjects.

AARA: Alpha-adrenergic receptor antagonists; ACEI: Angiotensin-converting enzyme inhibitors; ARB: Angiotensin receptor blockers; BB: beta-blockers; CCB: Calcium channel blockers; TD: thiazide diuretics; PB: placebo; RI: renin inhibitors.

**TABLE 4:** Pooled mean differences of different types of antihypertensive drugs on augmentation index including only studies longer than 6 months length. Upper right triangle gives the pooled mean differences from pairwise comparisons (column intervention relative to row), lower left triangle pooled mean differences from the network meta-analysis (row intervention relative to column). * Values *p* < 0.05 were considered significant.

PB	NA	**−1.64** **(−3.23, −0.06)** n^1^ = 4/n^2^ = 126	NA	NA	NA	NA	NA	NA	NA
−0.84 (−2.27, 0.59)	BB	NA	−0.36 (−0.73, 0.00) n^1^ = 2/n^2^ = 338	NA	NA	**−0.47** **(−0.67, −0.28)** n^1^ = 3/n^2^ = 602	NA	NA	NA
**−1.65** **(−2.52, -0.78)**	−0.81 (−1.94, 0.32)	ACEI	0.39 (−0.19, 0.96) n^1^ = 5/n^2^ = 300	NA	NA	NA	NA	NA	NA
**−1.21** **(−2.30, -0.12)**	−0.37 (−1.30, -0.55)	0.44 (−0.22, 1.10)	ARB	−0.02 (−0.30, 0.26) n^1^ = 1/n^2^ = 200	−0.29 (−1.02, 0.44) n^1^ = 1/n^2^ = 29	NA	NA	NA	NA
−1.12 (−2.85, 0.61)	−0.28 (−1.91, 1.35)	0.53 (−0.96, 2.02)	−0.09 (−1.43, 1.25)	CCB	NA	NA	0.06 (−0.28, 0.40) n^1^ = 1/n^2^ = 131	NA	NA
−1.51 (−3.35, 0.32)	−0.77 (−2.41, 1.07)	0.14 (−1.48,1.76)	−0.30 (−1.18, 1.78)	−0.39 (−2.38, 1.60)	RI	NA	NA	NA	NA
−1.21 (−2.92, 0.50)	−0.37 (−1.31, 0.57)	0.44 (−1.03,1.91)	−0.00 (−1.32, 1.32)	−0.09 (−1.97, 1.79)	0.30 (−1.68, 2.28)	TD/ACEI	NA	NA	NA
−0.94 (−2.95, 1.06)	−0.10 (−1.51, 1.31)	0.71 (−1.10,2.52)	−0.27 (−1.96, 1.42)	−0.18 (−2.33, 1.98)	0.57 (−1.67, 2.81)	0.27 (−0.79, 1.33)	TD/ARB	NA	0.16 (−0.03, 0.34) n^1^ = 1/n^2^ = 207
−1.29 (−3.30, 0.73)	−0.45 (−1.86, 0.97)	−0.37 (−2.18, 1.45)	−0.07 (−1.77, 1.62)	−0.16 (−2.32, 1.99)	−0.22 (−2.47, 2.02)	−0.08 (−1.14, 0.99)	−0.34 (−1.32, 0.63)	ACEI/CCB	−0.14 (−0.57, 0.30) n^1^ = 2/n^2^ = 128
−1.30 (−3.29, 0.69)	−0.46 (−1.84, 0.93)	−0.35 (−2.14, 1.4)	−0.09 (−1.75, 1.58)	−0.18 (−2.32, 1.96)	−0.21 (−2.44, 2.01)	−0.09 (−1.11, 0.93)	−0.36 (−1.19, 0.48)	−0.01 (−0.86, 0.84)	ARB/CCB

n^1^ = trials; n^2^ = subjects.

ACEI: Angiotensin-converting enzyme inhibitors; ARB: Angiotensin receptor blockers; BB: beta-blockers; CCB: Calcium channel blockers; PB: placebo; RI: renin inhibitors; TD: thiazide diuretics.

### 3.3 Treatment ranking

For PWV, the ACEI/ARB combination showed the highest SUCRA (93.0%) (Supplementary Table S6). For AIx, the ARB/CCB combination showed the highest SUCRA (78.0%) (Supplementary Table S7).

### 3.4 Subgroup analyses

Based on the type of PWV, beta-blockers (ES = −0.51; 95% CI: −0.99, −0.03), ACEI (ES = −0.85; 95% CI: −1.29, −0.40), ARB (ES = −0.48; 95% CI: −0.95, −0.02), the ACEI/ARB combination (ES = −1.11; 95% CI: −2.00, −0.22), the ACEI/CCB combination (ES = −1.11; 95% CI: −2.03, −0.18), the ARB/CCB combination (ES = −1.05; 95% CI: −2.02, −0.08), and the thiazide diuretics/ARB combination (ES = −0.93; 95% CI: −1.80, −0.07) were effective in reducing central PWV (Supplementary Figure S4; Supplementary Table S8), and ACEI/ARB combination (ES = −2.09; 95% CI: −4.17, −0.01) were effective in reducing peripheral PWV (Supplementary Figure S5 Supplementary Table S9).

Based on the type of population, the ACEI/ARB combination (ES = −0.83; 95% CI: −1.49, −0.17) and ACEI (ES = −0.43; 95% CI: −0.83, −0.02) were effective in reducing PWV (Supplementary Figure S6; Supplementary Table S10), and ACEI (ES = −0.64; 95% CI: −1.24, −0.04) was effective in reducing AIx in the population with exclusively hypertension (Supplementary Figure S7; Supplementary Table S11).

### 3.5 Random-effects meta-regression analyses

For PWV, random-effects meta-regression models showed that mean age and diastolic blood pressure reduction in ARB *versus* CCB and diastolic blood pressure reduction in ACEI *versus* CCB were related to pooled ES estimate (Supplementary Table S12).

For AIx, random-effects meta-regression models showed that systolic and diastolic blood pressure reduction in ACEI *versus* ARB were related to pooled ES estimates (Supplementary Table S13).

### 3.6 Sensitivity analysis, heterogeneity, and small study effect

For both PWV and AIx, the pooled ES was not significantly modified when the individual study data were removed, one at a time, from any pairwise comparison analysis.

Considerable heterogeneity was found for ACEI *versus* placebo for PWV and AIx (*I* ^2^ = 83.1, *τ* ^2^ = 0.81 and *I* ^2^ = 91.8, *τ* ^2^ = 1.40, respectively), ACEI *versus* ARB for PWV and AIx (*I* ^2^ = 81.5, *τ* ^2^ = 0.41 and *I* ^2^ = 83.5, *τ* ^2^ = 0.36, respectively), ARB *versus* the ACEI/ARB combination for PWV (*I* ^2^ = 91.3, *τ* ^2^ = 1.41), ARB *versus* placebo for AIx (*I* ^2^ = 92.5, *τ* ^2^ = 1.24), beta-blockers *versus* ACEI for AIx (*I* ^2^ = 80.4, *τ* ^2^ = 0.41), and diuretics *versus* ARB for AIx (*I* ^2^ = 93.6, *τ* ^2^ = 1.38) (Supplementary Tables S14, S15).

Finally, there was evidence of a small study effect in Egger’s test for ARB *versus* CCB (*p* = 0.005), diuretics *versus* CCB (*p* = 0.041) and the thiazide diuretic/ACEI combination *versus* the ACEI/CCB combination (*p* = 0.040) for PWV and for ACEI *versus* ARB (*p* = 0.039) for AIx.

## 4 Discussion

This NMA provides an overview of the evidence comparing the effect of antihypertensive drugs on reducing arterial stiffness in patients with hypertension. Although all types of hypertensive drugs reduced arterial stiffness measured by PWV and AIx, beta-blockers, ACEI, ARB, the ACEI/ARB combination, and the ARB/CCB combination showed a higher effect on reducing PWV, and ACEIs, ARBs, CCBs, renin inhibitors, the thiazide diuretic/ACEI combination and the ARB/CCB combination on reducing AIx. When only studies longer than 6 months length were included, thiazide diuretics, ACEI, ARB, the ACEI/ARB combination, the ACEI/CCB combination, and the ARB/CCB combination showed a higher effect on reducing PWV, and ACEIs and ARBs on reducing AIx. Additionally, when analyses were performed in patients with hypertension only (without other comorbidities), the ACEI/ARB combination and ACEI showed a higher effect on reducing PWV and AIx, respectively. Finally, beta-blockers, ACEI, ARB, the ACEI/ARB combination, the ACEI/CCB combination, the ARB/CCB combination, and the thiazide diuretics/ARB combination showed a statistically significant effect on reducing central PWV, and the ACEI/ARB combination on reducing peripheral PWV. However, it is worth noting that the ACEI/ARB combination of, despite showing improvement in stiffness measures, has been associated with an increased risk of cardiovascular events. As such, caution should be exercised when considering the use of this combination therapy in hypertensive patients, and alternative approaches may be warranted to mitigate potential risks. (Investigators et al., 2008).

Focusing on the results of the five classes of drugs used in clinical practice in the treatment of hypertension, we found that beta-blockers (high decrease for PWV), ACEIs (high decrease for PWV and AIx), ARBs (high decrease for PWV and moderate decrease for AIx) and CCBs (moderate decrease for AIx) are the pharmacological groups with the greatest effect in reducing arterial stiffness. The results found in this NMA on the effect of different types of antihypertensive drugs on arterial stiffness are consistent with data published in previous meta-analyses. Thus, the meta-analysis by Ong et al. (Ong et al., 2011) included 15 RCTs and 294 subjects with untreated hypertension, comparing the abovementioned antihypertensive drugs (except ARBs) *versus* placebo. They found that PWV decreased between −0.75 and −1.3 m/s in the treatment group compared with placebo, which decreased between −0.17 and −0.44 m/s. In short-term trials, ACEIs were more effective than CCBs, and in long-term trials, ACEIs, CCBs, beta-blockers and diuretics were more effective than placebo. The meta-analysis by Chen et al. (Chen et al., 2015) included 10 RCTs and 938 adults with hypertension and analysed the effects of ARBs *versus* other antihypertensive agents (except ACEIs) in reducing PWV and AIx. ARBs were not found to be superior to other types of antihypertensive agents in lowering PWV, but the ability of ARBs to improve Aix was superior. Another meta-analysis published by Shahin et al. (Shahin et al., 2012) analysed data from 469 patients included in 5 trials and evaluated the effect of ACEIs on arterial stiffness *versus* placebo or *versus* other antihypertensive agents (ARBs, CCBs, beta-blockers and diuretics). These authors concluded that ACEIs reduce PWV and Aix. However, due to the lack of high-quality and adequately powered RCTs, it was unclear whether the effect of ACEIs on arterial stiffness was superior to that of other antihypertensives. In the meta-analysis by Li et al., 2020, 17 RCTs, including 1458 individuals, analysed the effects of ACEIs on arterial stiffness. No significant differences were observed between ACEIs and controls for ba-PWV and cf-PWV in patients with hypertension, while the therapeutic effects of ACEIs *versus* placebo showed statistically significant differences. In the same vein, longitudinal studies that have analysed the effect of ACEIs or ARBs alone, in combination or in combination with CCBs or diuretics have been shown to be effective in reducing arterial stiffness, (Jatic et al., 2019), although the combination of ACEIs and ARBs is associated with more cardiovascular events than ACEIs alone. (Ma et al., 2010).

Thus, all results suggest that antihypertensive agents may have beneficial effects on arterial stiffness and central hemodynamic parameters, but the effect on arterial stiffness differs between them. Possible explanations for these differences include that ACEIs and ARBs influence arterial stiffness by reducing fibrosis and increasing arterial wall distensibility. In addition, some authors have suggested other mechanisms, such as reduction of oxidative stress, inflammation, and vasodilation through inhibition of angiotensin II, favoring vascular remodelling and endothelial function. (Peng et al., 2015; Cameron et al., 2016). The mechanism by which CCBs reduce arterial stiffness is related to the relaxation of arterial wall muscle cells. (Chen et al., 2015). For beta-blockers, the effect on PWV is moderate; however, they decrease central blood pressure and AIx with beta-blockers with vasodilator properties, probably due to an increase in nitric oxide levels, such as nebivolol, associated with the vasodilator effects of the drug, improving endothelial function and long-term reduction of arterial stiffness. (Kuyper and Khan, 2014). The effects of diuretics on measures of arterial stiffness have not been as well studied as other classes of drugs, and the possible mechanisms on arterial wall composition and arterial stiffness are not known. (Jatic et al., 2019). Therefore, to efficiently prescribe an antihypertensive drug, we must consider the effect on blood pressure as well as on arterial stiffness and other central hemodynamic parameters. (Chen et al., 2015).

Similarly, the effect varies according to the type of measure used to assess arterial stiffness, and we found less effect on PWV than on AIx. AIx is an index of wave reflection, which is influenced not only by arterial stiffness but also by microcirculatory status and cardiac contractility, which are highly dynamic, more than structural parameters such as arterial stiffness. (Climie et al., 2019).

Although the blood pressure response to different antihypertensive drug classes may differ according to the age of the patient, (Chen et al., 2015), we did not find a substantial effect of age in our meta-analysis. Finally, previous studies suggest that the effect of different antihypertensive drugs on arterial stiffness may vary according to the treatment duration; thus, beneficial effects have been obtained in the short term with some classes, while in the long term, most of them achieve beneficial effects on arterial stiffness. (Ong et al., 2011; Shahin et al., 2012). In contrast, we did not find any substantial effect of treatment duration in our meta-analysis.

The main limitations of this NMA are as follows: Some drug combinations in the treatment of hypertension, such as ACEIs and ARBs, have been included, (Yusuf et al., 2008), as well as some antihypertensive drugs, such as renin inhibitors, (Parving et al., 2012), as they do not provide additional benefits and increase the risk of adverse renal complications. Follow-up periods of RCTs vary widely, ranging from 4 to 208 weeks, and results from previous studies have shown that the effect of different antihypertensive drugs or their combinations on arterial stiffness in the short and long term is not the same and may influence the results obtained. (Ong et al., 2011). For this reason, in addition to the overall analysis, we performed analyses including only studies longer than 6 months length, to assess whether the duration of the intervention could modify our results. Heterogeneity in the studies, in terms of the number of subjects included, clinical characteristics of the subjects and health status of the subjects included in the different RCTs, may also have influenced the results obtained. Finally, we have not found any RCTs analysing the effect of antihypertensive drugs vs. placebo on CAVI, a parameter that estimates central and peripheral arterial stiffness. (Shirai et al., 2011). However, we should not forget that other drugs, such as statins or some treatments used in the treatment of type 2 diabetes mellitus, may influence arterial stiffness and have not been considered in this study.

Therefore, it is necessary to plan future RCTs with the 5 classes of drugs currently indicated for the treatment of hypertension: (Williams et al., 2018a): ACEIs, ARBs, beta-blockers, CCBs and diuretics (thiazides and thiazide analogues, such as chlorthalidone and indapamide and antiadosterone diuretics), since these drugs have been shown to be effective in reducing blood pressure as well as cardiovascular complications and overall cardiovascular morbidity and mortality. (Thomopoulos et al., 2017). In addition, we assessed the effect on arterial stiffness in the short and long term, including in the analysis all available evidence on the issue, both studies with positive and negative results, which is considered a strength for our results. This will help to clarify whether the effect of different antihypertensive drugs on arterial stiffness is independent of changes in blood pressure and to further investigate the mechanisms involved.

In summary, our study supports that antihypertensive drugs are a suitable treatment to reduce arterial stiffness in patients with hypertension. Based on our results, ACEIs, ARBs, beta-blockers, CCBs, renin inhibitors, the thiazide diuretics/ACEI combination, the ARB/CCB combination and the ACEI/ARB combination could be useful for patients with hypertension who have higher levels of arterial stiffness. Additionally, if we only consider antihypertensive treatments longer than 6 months, thiazide diuretics, ACEI, ARB, the ACEI/ARB combination, the ACEI/CCB combination, and the ARB/CCB combination are the most effective treatments. This result is clinically relevant since arterial stiffness is a closely related factor to hypertension, which produces a global burden of cardiovascular disease and is the leading cause of death and disability worldwide. However, it is worth noting that the ACEI/ARB combination, despite showing improvement in stiffness measures, has been associated with an increased risk of cardiovascular events. As such, caution should be exercised when considering the use of this combination therapy in hypertensive patients, and alternative approaches may be warranted to mitigate potential risks. Notwithstanding, it is essential that future well-designed, statistically powered RCTs are conducted to strengthen the currently weak evidence.

## Data Availability

The original contributions presented in the study are included in the article/Supplementary Material, further inquiries can be directed to the corresponding author.
