# Supplementary material for: Comparative effect of antihypertensive drugs in improving arterial stiffness in adults with hypertension (RIGIPREV study). A network meta-analysis
Source: Front Pharmacol. 2023 Sep 1;14:1225795. doi: 10.3389/fphar.2023.1225795 (PMC10505405; doi:10.3389/fphar.2023.1225795)
Supplement: Supplementary file 2 [file Table2.DOCX]

***Supplementary Material***

Comparative Effect of Antihypertensive Drugs in Improving Arterial Stiffness in Adults with Hypertension (RIGIPREV Study). A Network Meta-Analysis.

# Iván Cavero-Redondo1,2, Alicia Saz-Lara1*, Cristina Lugones-Sánchez3,4,5, Diana

**P. Pozuelo-Carrascosa1, Leticia Gómez-Sánchez3,4, José Francisco López-Gil6,7,8, Luis García-Ortiz3,4,5, Rosa Maria Bruno9, Manuel Ángel Gómez-Marcos3,4,5**

*** Correspondence:** Alicia Saz-Lara, [Alicia.delsaz@uclm.es](mailto:Alicia.delsaz@uclm.es)

**Table S1.** MEDLINE search strategy.

| “Hypertensive adults”  OR  “Hypertensive population”  OR  “Hypertensive subjects”  OR  “Arterial hypertension” | AND | “Antihypertensive treatment” OR “Antihypertensive drugs” OR “Beta- blockers” OR acebutolol OR atenolol OR atenolol OR betaxolol OR bisoprolol OR carteolol OR esmolol OR metoprolol OR nadolol OR oxprenolol OR penbutolol OR propranolol OR timolol OR celiprolol OR carvedilol OR labetalol OR nebivolol OR pindololol OR Diuretics OR furosemide OR bumetanide OR torsemide OR bendroflumethiazide OR chlorothiazide OR chlorthalidone OR hydrochlorothiazide OR indapamide OR polythiazide OR trichlormethiazide OR amiloride OR eplerenone OR spironolactone OR triamterene OR “Angiotensin-converting enzyme inhibitors” OR benazepril OR captopril OR cilazapril OR enalapril OR fosinopril OR imidapril OR lisinopril OR moexipril OR perindopril OR quinapril OR ramipril OR trandolapril OR zofenopril OR “Angiotensin II receptor antagonists” OR candesartan OR eprosartan OR irbesartan OR losartan OR olmesartan OR  telmisartan OR valsartan OR “Calcium channel blockers” OR diltiazem OR verapamil OR amlodipine OR felodipine OR isradipine OR lacidipine OR lercanidipine OR manidipine OR nicardipine OR “Renin inhibitors” OR aliskiren OR “Alpha-adrenergic receptor antagonists” OR doxazosin OR prazosin OR terazosin OR “Centrally acting agents”  OR clonidine OR methyl-dopa OR rilmenidine OR “Direct acting vasodilators” OR hydralazine OR minoxidine | AND | “Arterial stiffness”  OR  “Pulse wave velocity”  OR PWV  OR  “Augmentation index”  OR  Aix  OR  “Ambulatory arterial stiffness index”  OR AASI  OR  “Cardio-ankle vascular index”  OR  CAVI | AND | “Randomised controlled trial”  OR  “Randomized clinical trial”  OR  RCT |
| --- | --- | --- | --- | --- | --- | --- |

**Table S2.** List of Antihypertensive Drugs

| **Antihypertensive drug group** | | **Antihypertensive drug** |
| --- | --- | --- |
| Beta-blockers | | acebutolol, atenolol, atenolol, betaxolol, bisoprolol, carteolol, esmolol, metoprolol, nadolol, oxprenolol, penbutolol, propranolol, timolol, celiprolol, carvedilol, labetalol, nebivolol, pindololol |
| Diuretics | Thiazide diuretics | bendroflumethiazide, chlorothiazide, chlorthalidone, indapamide, hydrochlorothiazide, polythiazide, trichlormethiazide |
|  | Loop diuretics | furosemide, bumetanide, torsemide |
|  | Antialdosterone diuretics | amiloride, eplerenone, spironolactone, triamterene |
| Angiotensin-converting enzyme inhibitors | | benazepril, captopril, cilazapril, enalapril, fosinopril, imidapril, lisinopril, moexipril, perindopril, quinapril, ramipril, tran- dolapril, zofenopril |
| Angiotensin receptor blockers | | candesartan, eprosartan, irbesartan, losartan, olmesartan, telmisartan, valsartan |
| Calcium channel blockers | | diltiazem, verapamil, amlodipine, felodipine, isradipine, lacidipine,  lercanidipine, manidipine, nicardipine |
| Renin inhibitors | | aliskiren |
| Alpha-adrenergic receptor antagonists | | doxazosin, prazosin, terazosin |
| Centrally acting agents | | clonidine, methyl-dopa, rilmenidine |
| Direct-acting vasodilators | | hydralazine, minoxidil |

**Table S3.** Characteristics of the included studies.

| **Reference** | **Country** | **Study design** | **Population characteristics** | | | **Intervention: Antihypertensive drugs** | | | **Outcome: Arterial stiffness** | | |
| --- | --- | --- | --- | --- | --- | --- | --- | --- | --- | --- | --- |
|  |  |  | **Sample size (n,**  **%female**  **)** | **Mean age (years)** | **Type of population** | **Pharmacological group** | **Drug (dose and frequency)** | **Length (weeks)** | **Index** | **Device** | **Basal levels** |
| Asmar et al. | France | RCT | IG1: 8 | IG1: | Essential | IG1: CCB | IG1: Nitrendipine | 4 | cf-PWv | Doppler | cf-PWv |
| 1992 |  |  | (12.5) | 49.0±8.0 | hypertension | IG2: Placebo | (20mg daily) |  | ft-PWv |  | IG1: 10.7±2.5 |
|  |  |  | IG2: 9 | IG2: |  |  | IG2: Placebo |  | br-PWv |  | IG2: 9.8±1.6 |
|  |  |  | (22.2) | 51.0±7.0 |  |  |  |  |  |  |  |
|  |  |  |  |  |  |  |  |  |  |  | ft-PWv |
|  |  |  |  |  |  |  |  |  |  |  | IG1: 13.2±2.2 |
|  |  |  |  |  |  |  |  |  |  |  | IG2: 12.9±1.3 |
|  |  |  |  |  |  |  |  |  |  |  | br-PWv |
|  |  |  |  |  |  |  |  |  |  |  | IG1: 12.7±2.5 |
|  |  |  |  |  |  |  |  |  |  |  | IG2: 12.2±1.4 |
| Asmar et al. 1993 | France | Cross-over RCT | 16 (31.3) | 53.0±12.0 | Systemic hypertension | IG1: CCB  IG2: Thiazide diuretics | IG1: Felodipine (5- 10mg daily)  IG2: HCTZ (25-  50mg daily) | 12 | cf-PWv ft-PWv cr-PWv | Doppler | cf-PWv 11.0±2.0  ft-PWv 13.0±2.0  cr-PWv 12.0±2.0 |
| Merli et al. | Germany | RCT | IG1: 14 | IG1: | Hypertension | IG1: CCB | IG1: Isradipine | 12 | br-PWv | ES-2000 recorder | IG1: 10.6±1.9 |
| 1993 |  |  | (14.3) | 53.0±7.0 |  | IG2: Beta- | (2.5mg daily) |  |  |  | IG2: 10.3±3.4 |
|  |  |  | IG2: 14 | IG2: |  | blockers | IG2: Metoprolol |  |  |  |  |
|  |  |  | (14.3) | 51.0±9.0 |  |  | (50mg daily) |  |  |  |  |
| London et al. | France | RCT | IG1: 14 | IG1: | End-stage renal | IG1: ACEI | IG1: Perindopril | 48 | a-PWv | Doppler | a-PWv |
| 1994 |  |  | (50.0) | 54.7±2.7 | disease and | IG2: CCB | (2mg daily) |  | AIx |  | IG1: 12.9±2.6 |
|  |  |  | IG2: 10 | IG2: | predialysis |  | IG2: Nitrendipine |  |  |  | IG2: 13.1±3.8 |
|  |  |  | (30.0) | 51.7±4.9 | hypertension |  | (20mg daily) |  |  |  |  |
|  |  |  |  |  |  |  |  |  |  |  | AIx |
|  |  |  |  |  |  |  |  |  |  |  | IG1: |
|  |  |  |  |  |  |  |  |  |  |  | 28.3±11.9 |
|  |  |  |  |  |  |  |  |  |  |  | IG2: 30.1±7.6 |
| Chen et al, | China | RCT | IG1: 41 | IG1: | Hypertension | IG1: ACEI | IG1: Fosinopril (10- | 8 | AIx | Millar tonometers | IG1: |
| 1995 |  |  | (39.0) | 45.1±8.9 |  | IG2: Beta-blocker | 20mg daily) |  |  |  | 15.7±10.9 |
|  |  |  | IG2: 38 | IG2: |  |  | IG2: Atenolol (50- |  |  |  | IG2: |
|  |  |  | (28.9) | 46.6±8.0 |  |  | 100mg daily) |  |  |  | 15.9±11.0 |
| Breithaupt- Grogler et al. | Germany | RCT | IG1: 9 (NA) | 45-67 | Essential hypertension | IG1: ACEI  IG2: Thiazide | IG1: Cilazapril (5mg daily) | 12 | cf-PWv | Doppler | IG1: 9.4±2.7  IG2: 8.9±0.8 |
| 1996 |  |  | IG2: 8 |  |  | diuretics | IG2: HCTZ (25mg |  |  |  |  |
|  |  |  | (NA) |  |  |  | daily) |  |  |  |  |

| **Reference** | **Country** | **Study design** | **Population characteristics** | | | **Intervention: Antihypertensive drugs** | | | **Outcome: Arterial stiffness** | | |
| --- | --- | --- | --- | --- | --- | --- | --- | --- | --- | --- | --- |
|  |  |  | **Sample size (n,**  **%female**  **)** | **Mean age (years)** | **Type of population** | **Pharmacological group** | **Drug (dose and frequency)** | **Length (weeks)** | **Index** | **Device** | **Basal levels** |
| Savolainen et | Finland | RCT | IG1: 20 | IG1: | Essential | IG1: ACEI | IG1: Cilazapril | 12 and 24 | a-PWv | Doppler | IG1: 8.8±3.8 |
| al. 1996 |  |  | (NA) | 46.0±8.9 | hypertension | IG2: Beta-blocker | (5mg daily) |  |  |  | IG2: 7.7±2.3 |
|  |  |  | IG2: 20 | IG2: |  |  | IG2: Atenolol |  |  |  |  |
|  |  |  | (NA) | 47.5±9.4 |  |  | (100mg daily) |  |  |  |  |
| Armentano | USA | RCT | IG1: 17 | IG1: | Mild to moderate | IG1: ACEI | IG1: Ramipril | 12 | br-PWV | Millar tonometers | IG1: 11.6±1.7 |
| et al. 2001 |  |  | (12.5) | 57.0±7.0 | essential | IG2: Beta- | (2.5mg daily) |  |  |  | IG2: 11.7±1.3 |
|  |  |  | IG2: 17 | IG2: | hypertension | blockers | IG2: Atenolol |  |  |  |  |
|  |  |  | (35.3) | 53.0±8.0 |  |  | (50mg daily) |  |  |  |  |
| Asmar et al. | Multinational | RCT | IG1: 204 | IG1: NA | Essential and | IG1: ACEI + | IG1: Perindopril + | 52 | a-PWv | Complior | a-PWv |
| 2001 (a) |  |  | (NA) | IG2: NA | uncomplicated | Thiazide diuretics | Indapamide |  | c-AIx |  | IG1: 12.3±2.9 |
|  |  |  | IG2: 202 |  | hypertension | IG2: Beta- | (2mg/0.625mg |  | a-AIx |  | IG2: 12.3±2.8 |
|  |  |  | (NA) |  |  | blockers | daily) |  |  |  |  |
|  |  |  |  |  |  |  | IG2: Atenolol |  |  |  | c-AIx |
|  |  |  |  |  |  |  | (50mg daily) |  |  |  | IG1: |
|  |  |  |  |  |  |  |  |  |  |  | 27.9±18.5 |
|  |  |  |  |  |  |  |  |  |  |  | IG2: |
|  |  |  |  |  |  |  |  |  |  |  | 27.5±21.5 |
|  |  |  |  |  |  |  |  |  |  |  | a-AIx |
|  |  |  |  |  |  |  |  |  |  |  | IG1: |
|  |  |  |  |  |  |  |  |  |  |  | 29.5±10.1 |
|  |  |  |  |  |  |  |  |  |  |  | IG2: |
|  |  |  |  |  |  |  |  |  |  |  | 30.1±10.0 |
| Asmar et al. | Multinational | RCT | IG1: 70 | IG1: NA | Essential | IG1: ACEI + | IG1: Perindopril + | 52 | c-AIx | Complior | c-AIx |
| 2001 (b) |  |  | (NA) | IG2: NA | hypertension | Thiazide diuretics | Indapamide |  | a-AIx |  | IG1: |
|  |  |  | IG2: 74 |  |  | IG2: Beta- | (2mg/0.625mg |  |  |  | 27.9±18.5 |
|  |  |  | (NA) |  |  | blockers | daily) |  |  |  | IG2: |
|  |  |  |  |  |  |  | IG2: Atenolol |  |  |  | 27.5±21.5 |
|  |  |  |  |  |  |  | (50mg daily) |  |  |  |  |
|  |  |  |  |  |  |  |  |  |  |  | a-AIx |
|  |  |  |  |  |  |  |  |  |  |  | IG1: |
|  |  |  |  |  |  |  |  |  |  |  | 29.5±10.1 |
|  |  |  |  |  |  |  |  |  |  |  | IG2: |
|  |  |  |  |  |  |  |  |  |  |  | 30.1±10.0 |
| Dart et al. | Australia | RCT | IG1: 51 | IG1: | Hypertension | IG1: ACEI | IG1: Perindopril | 12 | AIx | PWV Medical | IG1: |
| 2001 |  |  | (58.8) | 59.5±1.6 |  | IG2: Placebo | (4mg daily) |  |  | Blood Pressure | 151.7±2.3 |
|  |  |  | IG2: 60 | IG2: |  |  | IG2: Placebo |  |  | Analysis System | IG2: |
|  |  |  | (46.7) | 61.8±1.4 |  |  |  |  |  |  | 149.5±2.7 |

| **Reference** | **Country** | **Study design** | **Population characteristics** | | | **Intervention: Antihypertensive drugs** | | | **Outcome: Arterial stiffness** | | |
| --- | --- | --- | --- | --- | --- | --- | --- | --- | --- | --- | --- |
|  |  |  | **Sample size (n,**  **%female**  **)** | **Mean age (years)** | **Type of population** | **Pharmacological group** | **Drug (dose and frequency)** | **Length (weeks)** | **Index** | **Device** | **Basal levels** |
| Klingbeil et | Germany | RCT | IG1: 20 | IG1: | Essential | IG1: ARB | IG1: Valsartan (80 | 6 | AIx | SphygmoCor | IG1: - |
| al. 2002 |  |  | (35.0) | 52.0±9.0 | hypertension | IG2: Thiazide | mg daily) |  |  |  | 21.7±10.5 |
|  |  |  | IG2: 20 | IG2: |  | diuretics | IG2: HCTZ (25mg |  |  |  | IG2: - |
|  |  |  | (40.0) | 55.0±11.0 |  | IG3: Placebo | daily) |  |  |  | 3.2±11.1 |
|  |  |  | IG3: 20 | IG3: |  |  | IG3: Placebo |  |  |  | IG3: 0.3±12.6 |
|  |  |  | (60.0) | 52.0±9.0 |  |  |  |  |  |  |  |
| Komai et al. | Japan | RCT | IG1:15 | IG1: | Hypertension | IG1: ACEI | IG1: Cilazapril | 24 | cf-PWV | FCP-4731 | IG1: 9.8±0.4 |
| 2002 |  |  | (33.3) | 63.9±3.8 |  | IG2: Beta- | (2mg daily) |  |  |  | IG2: 8.9±0.5 |
|  |  |  | IG2: 10 | IG2: |  | blockers | IG2: Atenolol |  |  |  |  |
|  |  |  | (40.0) | 59.3±2.9 |  |  | (25mg daily) |  |  |  |  |
| Mahmud and | Ireland | Crossover | IG1:12 | 49.0±11.0 | Hypertension | IG1: ARB | IG1: Valsartan | 4 | cf-PWV | Complior | cf-PWv |
| Feely 2002 |  |  | (NA) |  |  | IG2: ACEI | (160mg daily) |  | AIx |  | IG1: NA |
|  |  |  | IG2:12 |  |  | IG3: ARB + | IG2: Captopril |  |  |  | IG2: NA |
|  |  |  | (NA) |  |  | ACEI | (100mg daily) |  |  |  | IG3: NA |
|  |  |  | IG3:12 |  |  |  | IG3: Valsartan + |  |  |  |  |
|  |  |  | (NA) |  |  |  | Captopril |  |  |  | AIx |
|  |  |  |  |  |  |  | (160mg/100mg |  |  |  | IG1: NA |
|  |  |  |  |  |  |  | daily) |  |  |  | IG2: NA |
|  |  |  |  |  |  |  |  |  |  |  | IG3: NA |
| Rajzer et al. | Poland | RCT | IG1: 37 | 53.7±9.1 | Essential | IG1: ACEI | IG1: Amlodipine | 36 | cf-PWV | Complior | IG1: NA |
| 2003 |  |  | (NA) |  | hypertension | IG2: ARB | (10mg daily) |  |  |  | IG2: NA |
|  |  |  | IG2: 38 |  |  | IG3: CCB | IG2: Quinapril |  |  |  | IG3: NA |
|  |  |  | (NA) |  |  |  | (20mg daily) |  |  |  |  |
|  |  |  | IG3: 24 |  |  |  | IG3: Losartan (5mg |  |  |  |  |
|  |  |  | (NA) |  |  |  | daily) |  |  |  |  |
| Takami et al. | Japan | RCT | IG1: 20 | IG1: | Elderly | IG1: ARB | IG1: Valsartan | 12 | ba-PWv | Form/ABI | IG1: NA |
| 2003 |  |  | (0.0) | 71.0±3.0 | hypertensive | IG2: ACEI | (80mg daily) |  |  |  | IG2: NA |
|  |  |  | IG2: 20 | IG2: | patients | IG3: CCB | IG2: Temocapril: |  |  |  | IG3: NA |
|  |  |  | (0.0) | 71.4±2.7 |  | IG4: CCB | (2-4mg daily) |  |  |  | IG4: NA |
|  |  |  | IG3: 20 | IG3: |  |  | IG3: Cilnidipine |  |  |  |  |
|  |  |  | (0.0) | 72.0±3.0 |  |  | (10mg daily) |  |  |  |  |
|  |  |  | IG4: 16 | IG4: |  |  | IG4: Nifedipine CR |  |  |  |  |
|  |  |  | (0.0) | 72.8±2.7 |  |  | (20mg daily) |  |  |  |  |

| **Reference** | **Country** | **Study design** | **Population characteristics** | | | **Intervention: Antihypertensive drugs** | | | **Outcome: Arterial stiffness** | | | |
| --- | --- | --- | --- | --- | --- | --- | --- | --- | --- | --- | --- | --- |
|  |  |  | **Sample size (n,**  **%female**  **)** | **Mean age (years)** | **Type of population** | **Pharmacological group** | **Drug (dose and frequency)** | **Length (weeks)** | **Index** | **Device** | **Basal levels** | |
| White et al. | Multinational | RCT | IG1: 134 | IG1: | Systolic | IG1: AAD | IG1: Eplerenone | 14 and 24 | cf-PWv | Complior | IG1: 15.5±2.0 | |
| 2003 |  |  | (54.5) | 67.0±8.0 | hypertension | IG2: CCB | (50-200mg daily) |  |  |  | IG2: 16.5±3.6 | |
|  |  |  | IG2: 135 | IG2: |  |  | IG2: Amlodipine |  |  |  |  | |
|  |  |  | (51.1) | 69.0±8.0 |  |  | (2.5-10mg daily) |  |  |  |  | |
| De Luca et | France | RCT | IG1: 30 | IG1: | Essential | IG1: ACEI + | IG1: Perindopril + | 48 | c-AIx | SphygmoCor | IG1: 33.4±3.2 | |
| al. 2004 |  |  | (33.0) | 51.6±12.5 | hypertension | Thiazide diuretics | Indapamide |  |  |  | IG2: 32.8±3.2 | |
|  |  |  | IG2: 22 | IG2: |  | IG2: Beta- | (2mg/0.625mg |  |  |  |  | |
|  |  |  | (32.0) | 52.3±12.1 |  | blockers | daily) |  |  |  |  | |
|  |  |  |  |  |  |  | IG2: Atenolol |  |  |  |  | |
|  |  |  |  |  |  |  | (50mg daily) |  |  |  |  | |
| London et al. | Multinational | RCT | IG1: 235 | IG1: | Essential | IG1: ACEI + | IG1: Perindopril + | 48 | cf-PWv | Complior | cf-PWv | |
| 2004 |  |  | (34.0) | 55.0±12.2 | hypertension | Thiazide diuretics | Indapamide |  | c-AIx |  | IG1: 12.2±2.9 | |
|  |  |  | IG2: 234 | IG2: |  | IG2: Beta- | (2mg/0.625mg |  | a-AIx |  | IG2: 12.3±2.9 | |
|  |  |  | (31.0) | 54.8±12.0 |  | blockers | daily) |  |  |  |  | |
|  |  |  |  |  |  |  | IG2: Atenolol |  |  |  | c-AIx | |
|  |  |  |  |  |  |  | (50mg daily) |  |  |  | IG1: 24.4±1.9 | |
|  |  |  |  |  |  |  |  |  |  |  | IG2: 29.7±1.9 | |
|  |  |  |  |  |  |  |  |  |  |  | a-AIx | |
|  |  |  |  |  |  |  |  |  |  |  | IG1: | |
|  |  |  |  |  |  |  |  |  |  |  | 26.3±13.8 | |
|  |  |  |  |  |  |  |  |  |  |  | IG2: | |
|  |  |  |  |  |  |  |  |  |  |  | 30.2 | ±15.3 |
| Munakata et | Japan | RCT | IG1: 20 | IG1: | Essential | IG1: CCB | IG1: Nifedipine | 12 | ba-PWv | Form/ABI | IG1: 16.7±3.2 | |
| al. 2004 |  |  | (50.0) | 55.0±2.0 | hypertension | IG2: ARB | (20mg daily) |  |  |  | IG2: | |
|  |  |  | IG2: 21 | IG2: |  |  | IG2: Valsartan |  |  |  | 16.2±6.4 | |
|  |  |  | (52.4) | 53.0±3.0 |  |  | (80mg daily) |  |  |  |  | |
| Neal et al. | United | Crossover | IG1: 24 | IG1: 60 | Hypertension and | IG1: CCB | IG1: Amlodipine | 12 | AIx | SphygmoCor | IG1: 27.3±2.0 | |
| 2004 | Kingdom | RCT | (NA) | (36-74) | liver | IG2: Beta-blocker | (5/10mg daily) |  |  |  | IG2: 21.7±3.0 | |
|  |  |  | IG2: 11 | IG2: 55 | transplantation | IG3: ACEI | IG2: Bisoprolol |  |  |  | IG3: 24.9±1.9 | |
|  |  |  | (NA) | (36-67) IG3 |  |  | (5/10mg daily) |  |  |  |  | |
|  |  |  | IG3: 11 | :55 (36-67) |  |  | IG3: Lisinopril: |  |  |  |  | |
|  |  |  | (NA) |  |  |  | (5/10mg daily) |  |  |  |  | |

| **Reference** | **Country** | **Study design** | **Population characteristics** | | | **Intervention: Antihypertensive drugs** | | | **Outcome: Arterial stiffness** | | |
| --- | --- | --- | --- | --- | --- | --- | --- | --- | --- | --- | --- |
|  |  |  | **Sample size (n,**  **%female**  **)** | **Mean age (years)** | **Type of population** | **Pharmacological group** | **Drug (dose and frequency)** | **Length (weeks)** | **Index** | **Device** | **Basal levels** |
| Anan et al. | Japan | RCT | IG1: 10 | IG1: | Essential | IG1: ARB | IG1: Valsartan | 40 | ba-PWv | Form/ABI | IG1: 18.5±1.1 |
| 2005 |  |  | (60.0) | 59.0±8.0 | hypertension | IG2: ACEI | (160mg daily) |  |  |  | IG2: 18.2±1.4 |
|  |  |  | IG2: 11 | IG2: |  | IG3: ARB + ACE | IG2: Perindopril |  |  |  | IG3: 18.7±1.0 |
|  |  |  | (45.5) | 59.0±7.0 |  |  | (8mg daily) |  |  |  |  |
|  |  |  | IG3:10 | IG3: |  |  | IG3: Valsartan + |  |  |  |  |
|  |  |  | (60.0) | 58.0±7.0 |  |  | Perindopril |  |  |  |  |
|  |  |  |  |  |  |  | (80mg/4mg daily) |  |  |  |  |
| Ichihara et | Japan | RCT | IG1: 50 | IG1: | Hypertension | IG1: CCB | IG1: Amlodipine | 48 | cf-PWv | BP-203RPE | IG1: 17.2±3.5 |
| al. 2006 |  |  | (28.0) | 53.9±1.3 |  | IG2: ARB | (10mg daily) |  |  |  | IG2: 16.7±4.2 |
|  |  |  | IG2: 50 | IG2: |  |  | IG2: Valsartan |  |  |  |  |
|  |  |  | (24.0) | 54.3±1.4 |  |  | (320mg daily) |  |  |  |  |
| Kaiser et al. | Germany | Crossover | IG1:10 | 61.7 (52.1- | DM type 2 and | IG1: Beta- | IG1: Nebivolol | 12 | cr-PWv | SphygmoCor | cr-PWv |
| 2006 |  | RCT | (30.0) | 64.1) | hypertension | blockers | (5mg daily) |  | AIx |  | IG1: 10.2±1.2 |
|  |  |  | IG2: 10 |  |  | IG2: ACEI | IG2: Enalapril |  |  |  | IG2: 11.0±1.4 |
|  |  |  | (30.0) |  |  |  | (10mg daily) |  |  |  |  |
|  |  |  |  |  |  |  |  |  |  |  | AIx |
|  |  |  |  |  |  |  |  |  |  |  | IG1: |
|  |  |  |  |  |  |  |  |  |  |  | 138.0±19.6 |
|  |  |  |  |  |  |  |  |  |  |  | IG2: |
|  |  |  |  |  |  |  |  |  |  |  | 156.3±56.2 |
| Morimoto et | Japan | RCT | IG1: 22 | IG1: | Essential | IG1: CCB | IG1: Amlodipine | 24 | ba-PWv | Form/ABI | IG1: 16.1±2.8 |
| al. 2006 |  |  | (59.1) | 58.0±2.0 | hypertension | IG2: ARB | (5mg daily) |  |  |  | IG2: 16.9±3.2 |
|  |  |  | IG2: 21 | IG2: |  |  | IG2: Telmisartan |  |  |  |  |
|  |  |  | (57.1) | 56.0±2.0 |  |  | (40mg daily) |  |  |  |  |
| Yu et al. | Taiwan | RCT | IG1: 24 | IG1: | End-stage renal | IG1: ACEI | IG1: Ramipril | 26 and 52 | a-PWv | Doppler | a-PWv |
| 2006 |  |  | (29.0) | 45.0±13.0 | disease or regular | IG2: Placebo | (2.5mg 3 |  | AIx | Echoaortography | IG1: 8.4±3.0 |
|  |  |  | IG2: 22 | IG2: | hemodialysis |  | times/week) |  |  |  | IG2: 7.9±2.1 |
|  |  |  | (40.0) | 48.0±14.0 | terapy |  | IG2: Placebo |  |  |  |  |
|  |  |  |  |  |  |  |  |  |  |  | AIx |
|  |  |  |  |  |  |  |  |  |  |  | IG1: 5.0±22.0 |
|  |  |  |  |  |  |  |  |  |  |  | IG2: |
|  |  |  |  |  |  |  |  |  |  |  | 12.0±23.0 |
| Jiang et al. | China | RCT | IG1:51 | IG1: | Essential | IG1: ACEI | IG1: Enalapril | 8 | AIx | SphygmoCor | IG1: 33.7±9.8 |
| 2007 |  |  | (43.0) | 53.1±11.2 | hypertension | IG2: Thiazide | (10mg daily) |  |  |  | IG2: |
|  |  |  | IG2: 50 | IG2: |  | diuretics | IG2: Indapamide |  |  |  | 32.8±14.5 |
|  |  |  | (48.0) | 54.6±10.5 |  |  | (2.5mg daily) |  |  |  |  |

| **Reference** | **Country** | **Study design** | **Population characteristics** | | | **Intervention: Antihypertensive drugs** | | | **Outcome: Arterial stiffness** | | |
| --- | --- | --- | --- | --- | --- | --- | --- | --- | --- | --- | --- |
|  |  |  | **Sample size (n,**  **%female**  **)** | **Mean age (years)** | **Type of population** | **Pharmacological group** | **Drug (dose and frequency)** | **Length (weeks)** | **Index** | **Device** | **Basal levels** |
| Mitchell et | Canada | RCT | IG1: 152 | IG1: | Coronary disease | IG1: ACEI | IG1: Trandolapril | 208 | cf-PWv | Doppler | cf-PWv |
| al. 2007 |  |  | (11.0) | 64.2±7.9 | and preserved left | IG2: Placebo | (2mg daily) |  | AIx | Echoaortography | IG1: 10.4 |
|  |  |  | IG2: 148 | IG2: | ventricular |  | IG2: Placebo |  |  |  | (10.0-10.9) |
|  |  |  | (9.0) | 62.9±7.7 | function |  |  |  |  |  | IG2: 11.3 |
|  |  |  |  |  |  |  |  |  |  |  | (10.7-11.8) |
|  |  |  |  |  |  |  |  |  |  |  | AIx |
|  |  |  |  |  |  |  |  |  |  |  | IG1: 14.7 |
|  |  |  |  |  |  |  |  |  |  |  | (12.7-16.7) |
|  |  |  |  |  |  |  |  |  |  |  | IG2: 15.8 |
|  |  |  |  |  |  |  |  |  |  |  | (13.6-18.0) |
| Rahman et | Malaysia | RCT | DM type | DM type 2 | DM type 2 | IG1: ACEI | IG1: Ramipril (5mg | 48 | cf-PWv | SphygmoCor | DM type 2 |
| al. 2007 |  |  | 2 | IG1: | and IGT | IG2: Placebo | daily) |  | AIx |  | cf-PWv |
|  |  |  | IG1: 11 | 46.2±8.7 |  |  | IG2: Placebo |  |  |  | IG1: 10.7±3.5 |
|  |  |  | (NA) | IG2: |  |  |  |  |  |  | IG2: 10.1±1.9 |
|  |  |  | IG2: 10 | 46.1±8.3 |  |  |  |  |  |  |  |
|  |  |  | (NA) |  |  |  |  |  |  |  | AIx |
|  |  |  |  | IGT |  |  |  |  |  |  | IG1: |
|  |  |  | IGT | IG1: |  |  |  |  |  |  | 141.9±17.8 |
|  |  |  | IG1: 10 | 49.9±5.1 |  |  |  |  |  |  | IG2: |
|  |  |  | (NA) | IG2: |  |  |  |  |  |  | 132.8±15 IGT |
|  |  |  | IG2: 9 | 46.6±7.1 |  |  |  |  |  |  |  |
|  |  |  | (NA) |  |  |  |  |  |  |  | IGT |
|  |  |  |  |  |  |  |  |  |  |  | a-PWv |
|  |  |  |  |  |  |  |  |  |  |  | IG1: 10.2±2.2 |
|  |  |  |  |  |  |  |  |  |  |  | IG2: 8.8±1.1 |
|  |  |  |  |  |  |  |  |  |  |  | AIx |
|  |  |  |  |  |  |  |  |  |  |  | IG1: |
|  |  |  |  |  |  |  |  |  |  |  | 135.3±13.5 |
|  |  |  |  |  |  |  |  |  |  |  | IG2: |
|  |  |  |  |  |  |  |  |  |  |  | 139.8±17.2 |
| Rehman et | Malaysia | RCT | IG1: 19 | IG1: | Hypertension | IG1: ACEI | IG1: Losartan | 24 | cf-PWv | Complior | IG1: 12.0±1.8 |
| al. 2007 |  |  | (NA) | 52.5±7.6 |  | IG2: ARB | (50mg daily) |  |  |  | IG2: 11.0±1.7 |
|  |  |  | IG2: 20 | IG2: |  |  | IG2: Peridonpril |  |  |  |  |
|  |  |  | (NA) | 53.1±8.3 |  |  | (4mg daily) |  |  |  |  |

| **Reference** | **Country** | **Study design** | **Population characteristics** | | | **Intervention: Antihypertensive drugs** | | | **Outcome: Arterial stiffness** | | |
| --- | --- | --- | --- | --- | --- | --- | --- | --- | --- | --- | --- |
|  |  |  | **Sample size (n,**  **%female**  **)** | **Mean age (years)** | **Type of population** | **Pharmacological group** | **Drug (dose and frequency)** | **Length (weeks)** | **Index** | **Device** | **Basal levels** |
| Ahimastos et | Australia | RCT | IG1: 20 | 66.0±4.0 | Peripheral arterial | IG1: ACEI | IG1: Ramipril | 24 | Central | NA | Central PWv |
| al. 2008 |  |  | (NA) |  | disease | IG2: Placebo | (10mmg daily) |  | PWv |  | IG1: NA |
|  |  |  | IG2: 20 |  |  |  | IG2: Placebo |  | AIx |  | IG2: NA |
|  |  |  | (NA) |  |  |  |  |  |  |  |  |
|  |  |  |  |  |  |  |  |  |  |  | Aix |
|  |  |  |  |  |  |  |  |  |  |  | IG1: NA |
|  |  |  |  |  |  |  |  |  |  |  | IG2: NA |
| Dhakam et | UK | Crossover | IG1: 16 | 70.0±6.0 | Isolated systolic | IG1: Beta- | IG1: Atenolol | 5 | a-PWv | Doppler | a-PWv |
| al. 2008 |  | RCT | (37.5) |  | hypertension | blockers | (50mg daily) |  | AIx | Echoaortography | 10.2±1.5 |
|  |  |  | IG2: 16 |  |  | IG2: Beta- | IG2: Nevibolol |  |  |  |  |
|  |  |  | (37.5) |  |  | blockers | (5mg daily) |  |  |  | Aix |
|  |  |  | IG3: 16 |  |  | IG3: Placebo | IG3: Placebo |  |  |  | 24.0±8.0 |
|  |  |  | (37.5) |  |  |  |  |  |  |  |  |
| Ferguson et | Australia | Crossover | 28 (50.0) | IG1: | Isolated systolic | IG1: ACEI + | IG1: Fosinopril + | 8 | AIx | SphygmoCor | IG1: NA |
| al. 2008 |  |  |  | 62.0±1.4 | hypertension | Thiazide diuretics | HCTZ |  |  |  | IG2: NA |
|  |  |  |  | IG2: |  | IG2: CCB | (10mg/12.5mg |  |  |  | IG3: NA |
|  |  |  |  | 62.5±1.4 |  | IG3: Thiazide | daily) |  |  |  |  |
|  |  |  |  | IG3: |  | Diuretics | IG2: Amlodipine |  |  |  |  |
|  |  |  |  | 63.1±1.5 |  |  | (5mg daily) |  |  |  |  |
|  |  |  |  |  |  |  | IG3: Indapamide |  |  |  |  |
|  |  |  |  |  |  |  | (2.5mg daily) |  |  |  |  |
| Golavanova | Russia | RCT | IG1:13 | IG1: | Essential | IG1: Thiazide | IG1: Indapamide | 24 | CAVI | Vasera | IG1: 8.3±1.6 |
| et al. 2008 |  |  | (NA) | 44.7±10.1 | hypertension | diuretics | (1.5mg) |  |  |  | IG2: 7.4±1.3 |
|  |  |  | IG2:13 | IG2: |  | IG2: Beta- | IG2: Metoprolol |  |  |  | IG3: 8.0±1.2 |
|  |  |  | (NA) | 47.3±8.0 |  | blockers | (50mg) |  |  |  |  |
|  |  |  | IG3:13 | IG3: |  | IG3: ACEI + | IG3: Perindopril + |  |  |  |  |
|  |  |  | (NA) | 48.1±9.0 |  | Thiazide diuretics | Indapamide |  |  |  |  |
|  |  |  |  |  |  |  | (4mg/1.25mg daily) |  |  |  |  |
| Ishii et al. | Japan | RCT | IG1:11 | IG1: | DM type 2 and | IG1: ARB | IG1: Candesartan | 12 | ba-PWv | Form/ABI | IG1: 20.1±4.7 |
| 2008 |  |  | (NA) | 68.4±9.5 | hypertension | IG2: CCB | (8mg daily) |  |  |  | IG2: 19.9±6.4 |
|  |  |  | IG2: 11 | IG2: |  |  | IG2: Amlodipine |  |  |  |  |
|  |  |  | (NA) | 68.0±6.0 |  |  | (5mg daily) or |  |  |  |  |
|  |  |  |  |  |  |  | Nifedipine (40mg |  |  |  |  |
|  |  |  |  |  |  |  | daily) |  |  |  |  |

| **Reference** | **Country** | **Study design** | **Population characteristics** | | | **Intervention: Antihypertensive drugs** | | | **Outcome: Arterial stiffness** | | |
| --- | --- | --- | --- | --- | --- | --- | --- | --- | --- | --- | --- |
|  |  |  | **Sample size (n,**  **%female**  **)** | **Mean age (years)** | **Type of population** | **Pharmacological group** | **Drug (dose and frequency)** | **Length (weeks)** | **Index** | **Device** | **Basal levels** |
| Karalliedde | Switzerland | RCT | IG1: 73 | 40-80 | DM type 2 | IG1: ARB + | IG1: Valsartan + | 24 | a-PWv | NA | a-PWv |
| et al, 2008 |  |  | (NA) |  |  | Thiazide diuretics | Hydrochlorothiazid |  | AIx |  | IG1: 12.5±2.5 |
|  |  |  | IG2: 71 |  |  | IG2: CCB | e (160mg/25mg |  |  |  | IG2: 12.0±2.5 |
|  |  |  | (NA) |  |  |  | daily) |  |  |  |  |
|  |  |  |  |  |  |  | IG2: Amlodipine |  |  |  | AIx |
|  |  |  |  |  |  |  | (5-10mg daily) |  |  |  | IG1: 28.2±7.4 |
|  |  |  |  |  |  |  |  |  |  |  | IG2: 27.2±7.3 |
| Kosch et al | Germany | RCT | IG1:35 | IG1: | Essential | IG1: ARB | IG1: Valsartan (80- | 12 | a-PWv | Doppler | IG1: 10.9±1.9 |
| 2008 |  |  | (48.6) | 45.4±5.0 | hypertension | IG2: Beta- | 160mg daily) |  |  |  | IG2: 11.0±2.2 |
|  |  |  | IG2: 33 | IG2: |  | blockers | IG2: Metoprolol |  |  |  |  |
|  |  |  | (42.4) | 46.2±6.0 |  |  | (50-100mg daily) |  |  |  |  |
| Morimoto et | Japan | RCT | IG1: 16 | IG1: | Essential | IG1: CCB | IG1: Amlodipine | 24 | ba-PWv | Form/ABI | IG1: 16.2±2.4 |
| al. 2008 |  |  | (56.3) | 65.0±2.0 | hypertension | IG2: ACEI | (5mg daily) |  |  |  | IG2: 16.5±2.4 |
|  |  |  | IG2: 16 | IG2: |  |  | IG2: Peridonpril |  |  |  |  |
|  |  |  | (50.0) | 63.0±3.0 |  |  | (4mg daily) |  |  |  |  |
| Schneider et | Germany | RCT | IG1: 75 | IG1: | Essential | IG1: ARB | IG1: Irbesartan | 72 | AIx | SphygmoCor | IG1: |
| al 2008 |  |  | (48.0) | 53.0±8.0 | hypertension | IG2: Beta-blocker | (150mg daily) |  |  |  | 27.0±11.0 |
|  |  |  | IG2: 81 | IG2: |  |  | IG2: Atenolol |  |  |  | IG2: |
|  |  |  | (38.0) | 55.0±8.0 |  |  | (50mg daily) |  |  |  | 26.0±12.0 |
| Mackenzie et | UK | RCT | IG1: 15 | IG1: | Isolated systolic | IG1: ACEI | IG1: Perindopril | 10 | cf-PWv | SphygmoCor | cf-PWv |
| al. 2009 |  |  | (53.3) | 69.0±1.0 | hypertension | IG2: Beta- | (4mg daily) |  | AIx |  | IG1: 10.0±1.9 |
|  |  |  | IG2: 17 | IG2: |  | blockers | IG2: Atenolol |  |  |  | IG2: 9.8±2.5 |
|  |  |  | (47.0) | 69.0±1.0 |  | IG3: CCB | (50mg daily) |  |  |  | IG3: 9.8±2.2 |
|  |  |  | IG3: 14 | IG3: |  | IG4: Thiazide | IG3: Lercanidipine |  |  |  | IG4: 10.1±2.2 |
|  |  |  | (42.8) | 68.0±2.0 |  | diuretics | (10mg daily) |  |  |  |  |
|  |  |  | IG4: 13 | IG4: |  |  | IG4: |  |  |  | AIx |
|  |  |  | (46.2) | 68.0±2.0 |  |  | Bendrofluazide |  |  |  | IG1: |
|  |  |  |  |  |  |  | (2.5mg daily) |  |  |  | 28.0±11.6 |
|  |  |  |  |  |  |  |  |  |  |  | IG2: 31.0±8.3 |
|  |  |  |  |  |  |  |  |  |  |  | IG3: |
|  |  |  |  |  |  |  |  |  |  |  | 26.0±11.2 |
|  |  |  |  |  |  |  |  |  |  |  | IG4: |
|  |  |  |  |  |  |  |  |  |  |  | 27.0±10.8 |

| **Reference** | **Country** | **Study design** | **Population characteristics** | | | **Intervention: Antihypertensive drugs** | | | **Outcome: Arterial stiffness** | | |
| --- | --- | --- | --- | --- | --- | --- | --- | --- | --- | --- | --- |
|  |  |  | **Sample size (n,**  **%female**  **)** | **Mean age (years)** | **Type of population** | **Pharmacological group** | **Drug (dose and frequency)** | **Length (weeks)** | **Index** | **Device** | **Basal levels** |
| Matsui et al. | Japan | RCT | IG1: 103 | IG1: | Hypertension | IG1: ARB + CCB | IG1: Olmesartan + | 12 | cf-PWv | SphygmoCor | cf-PWv |
| 2009 |  |  | (60.0) | 68.9±8.1 |  | IG2: ARB + | Azelnidipine |  | AIx |  | IG1: 10.2±2.0 |
|  |  |  | IG2: 104 | IG2: |  | Thiazide diuretics | (20mg/16mg daily) |  |  |  | IG2: 10.3±2.2 |
|  |  |  | (60.0) | 68.0±9.1 |  |  | IG2: Olmesartan + |  |  |  |  |
|  |  |  |  |  |  |  | HCTZ |  |  |  | AIx |
|  |  |  |  |  |  |  | (20mg/12.5mg |  |  |  | IG1: 34.7±6.3 |
|  |  |  |  |  |  |  | daily) |  |  |  | IG2: 34.6±8.1 |
| Musikhina et | Russia | RCT | IG1: 15 | IG1: | Hypertension | IG1: CCB | IG1: Felodipine (5- | 8 | cf-PWv | NA | cf-PWv |
| al. 2009 |  |  | (NA) | 56.3±1.3 |  | IG2: ACEI | 10mg daily) |  | cr-PWv |  | IG1: 9.6±1.5 |
|  |  |  | IG2: 19 | IG2: |  |  | IG2: Perindopril (4- |  |  |  | IG2: 9.6±1.7 |
|  |  |  | (NA) | 55.3±1.4 |  |  | 8mg daily) |  |  |  |  |
|  |  |  |  |  |  |  |  |  |  |  | cr-PWv |
|  |  |  |  |  |  |  |  |  |  |  | IG1: 9.0±1.5 |
|  |  |  |  |  |  |  |  |  |  |  | IG2: 9.8±2.2 |
| Li et al. 2009 | China | RCT | IG1: 34 | IG1: | Hypertension | IG1: ACEI | IG1: Peridonpril | 12 | ba-PWv | BP-203RPE | IG1:18.6±4.9 |
|  |  |  | (38.2) | 58.0±9.8 |  | IG2: | (4mg daily) |  |  |  | IG2: 17.8±3.4 |
|  |  |  | IG2: 34 | IG2: |  | CCB | IG2: Amlodipine |  |  |  | IG3: 18.6±3.4 |
|  |  |  | (32.4) | 57.1±11.1 |  | IG3: ARB | (5mg daily) |  |  |  |  |
|  |  |  | IG3: 34 | IG3: |  |  | IG3: Telmisartan |  |  |  |  |
|  |  |  | (35.3) | 58.3±9.7 |  |  | (80mg daily) |  |  |  |  |
| Moltzer et al. | USA | Crossover | IG1: 15 | 37.0±12.4 | Hypertension | IG1: ARB | IG1: Candesartan | 8 | ff-PWv | Complior | IG1: 6.8±1.6 |
| 2010 |  | RCT | (NA) |  | after repaired | IG2: Beta-blocker | (8mg daily) |  |  |  | IG2: 7.3±1.1 |
|  |  |  | IG2: 15 |  | aortic coarctation |  | IG2: Metoprolol |  |  |  |  |
|  |  |  | (NA) |  |  |  | (100mg daily) |  |  |  |  |
| Tomiyama et | Japan | RCT | IG1: 56 | IG1: | Hypertension | IG1: ARB | IG1: Candersartan | 104 | ba-PWv | Form/ABI | IG1: 17.4±3.6 |
| al. 2011 |  |  | (35.7) | 56.0±10.0 |  | IG2: CCB | (8mg daily) |  |  |  | IG2: 17.7±3.6 |
|  |  |  | IG2: 57 | IG2: |  |  | IG2: Amlodipine |  |  |  |  |
|  |  |  | (36.8) | 58.0±11.0 |  |  | (5mg daily) |  |  |  |  |
| Hayoz et al. 2012 | Swizterla nd | RCT | IG1: 63  (100.0)  IG2: 62  (100.0) | IG1: 62.3±5.8 IG2: 60.4±5.1 | Postmenopausal hypertensive women | IG1: ARB +  Thiazide diuretics IG2: CCB +  Thiazide diuretics | IG1: Valsartan + HCTZ (320  mg/12.5mg daily) IG2: Amlodipine + HCTZ (10  mg/12.5mg daily) | 42 | cf-PWv | Transcut aneous Doppler | IG1: 14.0±3.4  IG2: 13.6±3.2 |

| **Reference** | **Country** | **Study design** | **Population characteristics** | | | **Intervention: Antihypertensive drugs** | | | **Outcome: Arterial stiffness** | | |
| --- | --- | --- | --- | --- | --- | --- | --- | --- | --- | --- | --- |
|  |  |  | **Sample size (n,**  **%female**  **)** | **Mean age (years)** | **Type of population** | **Pharmacological group** | **Drug (dose and frequency)** | **Length (weeks)** | **Index** | **Device** | **Basal levels** |
| Matsui et al. | Japan | RCT | IG1: 103 | IG1: | Hypertension | IG1: ARB + CCB | IG1: Olmesartan + | 12 | cf-PWv | SphygmoCor | IG1: NA |
| 2012 |  |  | (60.0) | 68.9±8.1 |  | IG2: ARB + | Azelnidipine |  |  |  | IG2: NA |
|  |  |  | IG2: 104 | IG2: |  | Thiazide diuretics | (20mg/16mg daily) |  |  |  |  |
|  |  |  | (60.0) | 68.0±9.1 |  |  | IG2: Olmesartan + |  |  |  |  |
|  |  |  |  |  |  |  | HCTZ |  |  |  |  |
|  |  |  |  |  |  |  | (20mg/12.5mg |  |  |  |  |
|  |  |  |  |  |  |  | daily) |  |  |  |  |
| Nedogoba et | Russia | RCT | IG1: 30 | IG1: | Hypertension and | IG1: ACEI | IG1: Peridonpril | 24 | cf-PWv cr-PWv AIx | Complior | cf-PWv |
| al. 2012 |  |  | (46.7) | 49.7±8.2 | obesity | IG2: ARB | (10mg daily) |  |  | Sphygmocor | IG1: 12.8±1.7 |
|  |  |  | IG2: 30 | IG2: |  |  | IG2: Losartan |  |  |  | IG2: 12.1±1.6 |
|  |  |  | (60.0) | 46.7±8.2 |  |  | (100mg daily) |  |  |  |  |
|  |  |  |  |  |  |  |  |  |  |  | cr-PWv |
|  |  |  |  |  |  |  |  |  |  |  | IG1: 12.4±1.6 |
|  |  |  |  |  |  |  |  |  |  |  | IG2: 12.1±2.2 |
|  |  |  |  |  |  |  |  |  |  |  | AIx: |
|  |  |  |  |  |  |  |  |  |  |  | IG1: 44.9±5.3 |
|  |  |  |  |  |  |  |  |  |  |  | IG2: 39.8±4.1 |
| Spanos et al. | Greece | RCT | IG1: 15 | IG1: | Hypertension | IG1: Renin | IG1: Aliskiren | 24 v | cf-PWv | SphygmoCor | cf-PWv |
| 2012 |  |  | (30.0) | 55.0±13.0 | with albuminuria | inhibitor | (150mg daily) |  | cr-PWv |  | IG1: 9.4±2.1 |
|  |  |  | IG2: 14 | IG2: |  | IG2: ARB | IG2: Valsartan |  | AIx |  | IG2: 11.1±1.7 |
|  |  |  | (28.6) | 60.0±17.0 |  |  | (160mg daily) |  |  |  |  |
|  |  |  |  |  |  |  |  |  |  |  | cr-PWv |
|  |  |  |  |  |  |  |  |  |  |  | IG1: 8.6±1.6 |
|  |  |  |  |  |  |  |  |  |  |  | IG2: 7.5±1.2 |
|  |  |  |  |  |  |  |  |  |  |  | AIx |
|  |  |  |  |  |  |  |  |  |  |  | IG1: 30.3±8.8 |
|  |  |  |  |  |  |  |  |  |  |  | IG2: |
|  |  |  |  |  |  |  |  |  |  |  | 29.1±13.2 |

| **Reference** | **Country** | **Study design** | **Population characteristics** | | | **Intervention: Antihypertensive drugs** | | | **Outcome: Arterial stiffness** | | |
| --- | --- | --- | --- | --- | --- | --- | --- | --- | --- | --- | --- |
|  |  |  | **Sample size (n,**  **%female**  **)** | **Mean age (years)** | **Type of population** | **Pharmacological group** | **Drug (dose and frequency)** | **Length (weeks)** | **Index** | **Device** | **Basal levels** |
| Tishina et al, | Russia | RCT | IG1: 20 | IG1: | Hypertension and | IG1: DAV + CCB | IG1: Monoxidine + | 24 | a- PWv | NA | IG1: 11.3±4.6 |
| 2012 |  |  | (65.0) | 54.1±7.2 | metabolic | IG2: DAV + | Amlodipine (0.2- |  |  |  | IG2: 10.1±2.3 |
|  |  |  | IG2: 20 | IG2: | syndrome | Thiazide diuretics | 0.4mg/5-10mg |  |  |  | IG3: 9.7±2.8 |
|  |  |  | (60.0) | 52.7±8.2 |  | IG3: DAV + | daily) |  |  |  |  |
|  |  |  | IG3: 20 | IG3: |  | ACEI | IG2: Monoxidine + |  |  |  |  |
|  |  |  | (65.0) | 56.2±7.6 |  |  | HCTZ (0.2- |  |  |  |  |
|  |  |  |  |  |  |  | 0.4mg/12.5mg |  |  |  |  |
|  |  |  |  |  |  |  | daily) |  |  |  |  |
|  |  |  |  |  |  |  | IG3: Monoxidine + |  |  |  |  |
|  |  |  |  |  |  |  | Enalapril (0.2- |  |  |  |  |
|  |  |  |  |  |  |  | 0.4mg/10-20mg |  |  |  |  |
|  |  |  |  |  |  |  | daily) |  |  |  |  |
| Virdis et al. | Italy | RCT | IG1: 25 | IG1: | Essential | IG1: Renin | IG1: Aliskiren | 12 | cf-PWv | SphygmoCor | cf-PWv |
| 2012 |  |  | (28.0) | 44.9±8.1 | hypertension | inhibitor | (150-300mg daily) |  | AIx |  | IG1: 7.7±3.5 |
|  |  |  | IG2: 25 | IG2: |  | IG2: ACEI | IG2: Ramipril (5- |  |  |  | IG2: 7.5±3.0 |
|  |  |  | (32.0) | 45.1±6.3 |  |  | 10mg daily) |  |  |  |  |
|  |  |  |  |  |  |  |  |  |  |  | AIx |
|  |  |  |  |  |  |  |  |  |  |  | IG1: |
|  |  |  |  |  |  |  |  |  |  |  | 18.0±15.5 |
|  |  |  |  |  |  |  |  |  |  |  | IG2: |
|  |  |  |  |  |  |  |  |  |  |  | 18.8±16.5 |
| Vitale et al. | Italy | RCT | IG1: 31 | IG1: | Essential | IG1: Beta-blocker | IG1: Nebivolol + | 8 | cf-PWv | SphygmoCor | cf-PWv |
| 2012 |  |  | (35.0) | 54.5±8.7 | hypertension | + Thiazide | HCTZ |  | AIx |  | IG1: 11.5±1.6 |
|  |  |  | IG2: 34 | IG2: |  | diuretics | (5mg/12.5mg daily) |  |  |  | IG2: 11.3±2.2 |
|  |  |  | (35.5) | 52.7±11.4 |  | IG2: CCB + | IG2: Irbesartan + |  |  |  |  |
|  |  |  |  |  |  | Thiazide diuretics | HCTZ |  |  |  | AIx |
|  |  |  |  |  |  |  | (150mg/12.5mg |  |  |  | IG1: |
|  |  |  |  |  |  |  | daily) |  |  |  | 30.9±10.9 |
|  |  |  |  |  |  |  |  |  |  |  | IG2: |
|  |  |  |  |  |  |  |  |  |  |  | 29.3±10.1 |

| **Reference** | **Country** | **Study design** | **Population characteristics** | | | **Intervention: Antihypertensive drugs** | | | **Outcome: Arterial stiffness** | | |
| --- | --- | --- | --- | --- | --- | --- | --- | --- | --- | --- | --- |
|  |  |  | **Sample size (n,**  **%female**  **)** | **Mean age (years)** | **Type of population** | **Pharmacological group** | **Drug (dose and frequency)** | **Length (weeks)** | **Index** | **Device** | **Basal levels** |
| Agnoletti et | Multinational | RCT | IG1: 33 | IG1: | Essential | IG1: CCB | IG1: Amlodipine | 12 | cf-PWv | SphygmoCor | cf-PWv |
| al. 2013 |  |  | (48.0) | 59.5±11.6 | hypertension | IG2: ARB | (5mg daily) |  | AIx |  | IG1: 10.3±3.2 |
|  |  |  | IG2: 33 | IG2: |  | IG3: Thiazide | IG2: Candesartan |  |  |  | IG2: 9.6±2.2 |
|  |  |  | (45.0) | 57.2±8.6 |  | diuretics | (8mg daily) |  |  |  | IG3: 9.7±2.7 |
|  |  |  | IG3: 44 | IG3: |  | IG4: Placebo | IG3: Indapamide |  |  |  | IG4: 9.7±3.1 |
|  |  |  | (43.0) | 59.2±10.6 |  |  | (1.5 mg daily) |  |  |  |  |
|  |  |  | IG4: 35 | IG4: |  |  | IG4: Placebo |  |  |  | AIx |
|  |  |  | (54.0) | 58.2±10.7 |  |  |  |  |  |  | IG1: |
|  |  |  |  |  |  |  |  |  |  |  | 24.7±15.4 |
|  |  |  |  |  |  |  |  |  |  |  | IG2: |
|  |  |  |  |  |  |  |  |  |  |  | 26.5±16.0 |
|  |  |  |  |  |  |  |  |  |  |  | IG3: |
|  |  |  |  |  |  |  |  |  |  |  | 25.2±10.9 |
|  |  |  |  |  |  |  |  |  |  |  | IG4: |
|  |  |  |  |  |  |  |  |  |  |  | 30.5±12.3 |
| Hare et al. | Australia | RCT | IG1: 58 | IG1: | Hypertension | IG1: AAD | IG1: Spironolactone | 12 | cf-PWv | SphygmoCor | IG1: 8.4±1.9 |
| 2013 |  |  | (43.0) | 54.0±9.0 |  | IG2: Placebo | (25mg daily) |  |  |  | IG2: 8.2±1.7 |
|  |  |  | IG2: 52 | IG2: |  |  | IG2: Placebo |  |  |  |  |
|  |  |  | (44.0) | 55.0±8.0 |  |  |  |  |  |  |  |
| Hyun et al. | Korea | RCT | IG1:101 | IG1: | Essential | IG1: CCB | IG1: Benidipine | 24 | Central | PP-1000 | Central PWv |
| 2013 |  |  | (45.5) | 51.1±7.7 | hypertension | IG2: ARB | (4mg daily) |  | PWv | GAON21A | IG1: 7.8±1.2 |
|  |  |  | IG2: 99 | IG2: |  |  | IG2: Losartan |  | Brachial |  | IG2: 8.1±1.2 |
|  |  |  | (38.4) | 50.6±8.7 |  |  | (50mg daily) |  | PWv |  |  |
|  |  |  |  |  |  |  |  |  | Femoral |  | Brachial PWv |
|  |  |  |  |  |  |  |  |  | PWv |  | IG1: 8.5±1.3 |
|  |  |  |  |  |  |  |  |  | AIx |  | IG2: 9±1.1 |
|  |  |  |  |  |  |  |  |  |  |  | Femoral PWv |
|  |  |  |  |  |  |  |  |  |  |  | IG1: 10.6±1.5 |
|  |  |  |  |  |  |  |  |  |  |  | IG2: 10±1.7 |
|  |  |  |  |  |  |  |  |  |  |  | AIx |
|  |  |  |  |  |  |  |  |  |  |  | IG1: |
|  |  |  |  |  |  |  |  |  |  |  | 30.1±19.1 |
|  |  |  |  |  |  |  |  |  |  |  | IG2: |
|  |  |  |  |  |  |  |  |  |  |  | 30.2±12.9 |

| **Reference** | **Country** | **Study design** | **Population characteristics** | | | **Intervention: Antihypertensive drugs** | | | **Outcome: Arterial stiffness** | | |
| --- | --- | --- | --- | --- | --- | --- | --- | --- | --- | --- | --- |
|  |  |  | **Sample size (n,**  **%female**  **)** | **Mean age (years)** | **Type of population** | **Pharmacological group** | **Drug (dose and frequency)** | **Length (weeks)** | **Index** | **Device** | **Basal levels** |
| Koumaras et | Greece | RCT | IG1: 20 | IG1: | Essential | IG1: ACEI | IG1: Quinalapril | 10 | cf-PWv | SphygmoCor | cf-PWv |
| al. 2013 |  |  | (25.0) | 48.4±11.9 | hypertension | IG2: Renin | (20mg daily) |  | AIx |  | IG1: 9.2±1.9 |
|  |  |  | IG2:18 | IG2: |  | inhibitors | IG2: Aliskiren |  |  |  | IG2: 8.7±1.9 |
|  |  |  | (50.0) | 48.4±10.5 |  | IG3: Beta- | (150mg daily) |  |  |  | IG3: 8.4±1.5 |
|  |  |  | IG3: 17 | IG3: |  | blockers | IG3: Atenolol |  |  |  | IG4: 8.2±1.2 |
|  |  |  | (17.6) | 44.7±10.2 |  | IG4: Beta- | (50mg daily) |  |  |  |  |
|  |  |  | IG4: 17 | IG4: |  | blockers | IG4: Nevibolol |  |  |  | AIx |
|  |  |  | (35.3) | 48.8±9.8 |  |  | (5mg daily) |  |  |  | IG1: 26.6±8.3 |
|  |  |  |  |  |  |  |  |  |  |  | IG2: 33.6±7.6 |
|  |  |  |  |  |  |  |  |  |  |  | IG3: 22.8±8.4 |
|  |  |  |  |  |  |  |  |  |  |  | IG4: |
|  |  |  |  |  |  |  |  |  |  |  | 32.1±10.9 |
| Nedogoba et | Russia | RCT | IG1: 27 | IG1: | Hypertension | IG1: ACEI + | IG1: Lisinopril + | 36 | cf-PWv | Complior | cf-PWv |
| al. 2013 (a) |  |  | (55.6) | 60.6±8.2 |  | CCB | Amlodipine |  | cr-PWv |  | IG1: 13.4±1.5 |
|  |  |  | IG2: 27 | IG2: |  | IG2: ACEI + | (10mg/5mg daily) |  |  |  | IG2: 13.1±3.6 |
|  |  |  | (68.0) | 63.7±5.2 |  | Thiazide diuretics | IG2: Enalapril + |  |  |  |  |
|  |  |  |  |  |  |  | HCTZ |  |  |  | cr-PWv |
|  |  |  |  |  |  |  | (20mg/12.5mg |  |  |  | IG1: 10.3±1.7 |
|  |  |  |  |  |  |  | daily) |  |  |  | IG2: 10.6±2.9 |
| Nedogoda et | Russia | RCT | IG1: 30 | IG1: | Hypertension | IG1: ACEI | IG1: Perindopril | 24 | cf-PWv cr-PWv AIx | Complior | cf-PWv |
| al. 2013 (b) |  |  | (46.7) | 49.7±8.2 |  | IG2: ACEI | (10mg daily) |  |  | Sphygmocor | IG1: 12.8±1.7 |
|  |  |  | IG2: 30 | IG2: |  | IG3: ARB | IG2: Enalapril |  |  |  | IG2: 12.1±1.5 |
|  |  |  | (50.0) | 47.8±8.1 |  | IG4: ARB | (20mg daily) |  |  |  | IG3: 12.1±1.9 |
|  |  |  | IG3: 30 | IG3: |  |  | IG3: Losartan |  |  |  | IG4: 12.2±1.7 |
|  |  |  | (50.0) | 46.7±8.2 |  |  | (100mg daily) |  |  |  |  |
|  |  |  | IG4: 30 | IG4: |  |  | IG4: Telmisartan |  |  |  | cr-PWv |
|  |  |  | (50.0) | 47.4±9.2 |  |  | (80mg daily) |  |  |  | IG1: 12.4±1.6 |
|  |  |  |  |  |  |  |  |  |  |  | IG2: 11.9±1.5 |
|  |  |  |  |  |  |  |  |  |  |  | IG3: 12.1±2.2 |
|  |  |  |  |  |  |  |  |  |  |  | IG4: 11.9±1.6 |
|  |  |  |  |  |  |  |  |  |  |  | AIx: |
|  |  |  |  |  |  |  |  |  |  |  | IG1: 45.5±5.0 |
|  |  |  |  |  |  |  |  |  |  |  | IG2: 41.5±5.0 |
|  |  |  |  |  |  |  |  |  |  |  | IG3: 40.0±4.0 |
|  |  |  |  |  |  |  |  |  |  |  | IG4: 43.0±5.0 |

| **Reference** | **Country** | **Study design** | **Population characteristics** | | | **Intervention: Antihypertensive drugs** | | | **Outcome: Arterial stiffness** | | |
| --- | --- | --- | --- | --- | --- | --- | --- | --- | --- | --- | --- |
|  |  |  | **Sample size (n,**  **%female**  **)** | **Mean age (years)** | **Type of population** | **Pharmacological group** | **Drug (dose and frequency)** | **Length (weeks)** | **Index** | **Device** | **Basal levels** |
| Radchenko | Ukraine | RCT | IG1: 32 | IG1: | Hypertension | IG1: ARB + | IG1: Losartan + | 12 | cf-PWv | SphygmoCor | cf-PWv |
| et al. 2013 |  |  | (50.0) | 56.2±2.0 |  | Thiazide diuretics | HCTZ |  | AIx |  | IG1: 13.2±2.8 |
|  |  |  | IG2: 27 | IG2: |  | IG2: Beta-blocker | (100mg/25mg |  |  |  | IG2: 13.1±3.6 |
|  |  |  | (33.5) | 51.3±3.4 |  | + Thiazide | daily) |  |  |  |  |
|  |  |  |  |  |  | diuretics | IG2: Bisoprolol + |  |  |  | AIx: |
|  |  |  |  |  |  |  | HCTZ (10mg/25mg |  |  |  | IG1: |
|  |  |  |  |  |  |  | daily) |  |  |  | 23.1±10.2 |
|  |  |  |  |  |  |  |  |  |  |  | IG2: 19.7±8.8 |
| Zhou et al. | China | RCT | IG1: 54 | IG1: | Essential | IG1: Beta-blocker | IG1: Bisoprolol | 4 and 8 | AIx | SphygmoCor | IG1: |
| 2013 |  |  | (31.5) | 43.1±9.8 | hypertension | IG2: Beta-blocker | (5mg daily) |  |  |  | 25.8±11.8 |
|  |  |  | IG2: 55 | IG2: |  |  | IG2: Atenolol |  |  |  | IG2: |
|  |  |  | (31.0) | 44.7±10.9 |  |  | (50mg daily) |  |  |  | 25.3±14.3 |
| Kim et al. | Korea | RCT | IG1:88 | IG1: | Hypertension | IG1: ARB | IG1: Losartan | 24 | cf-PWv | PP-1000 | cf-PWv |
| 2014 |  |  | (37.5) | 48.7±9.5 |  | IG2: Beta- | (50mg daily) |  | AIx | Gaon21A System | IG1: 7.5±1.3 |
|  |  |  | IG2: 94 | IG2: |  | blockers | IG2: Carvedilol |  |  |  | IG2: 7.7±1.42 |
|  |  |  | (43.6) | 50.5±10.3 |  |  | (12.5mg daily) |  |  |  |  |
|  |  |  |  |  |  |  |  |  |  |  | AIx |
|  |  |  |  |  |  |  |  |  |  |  | IG1: |
|  |  |  |  |  |  |  |  |  |  |  | 26.2±10.9 |
|  |  |  |  |  |  |  |  |  |  |  | IG2: |
|  |  |  |  |  |  |  |  |  |  |  | 29.1±14.4 |
| Pozzobon et | Brazil | RCT | IG1: 21 | IG1: | DM type 2 and | IG1: ARB | IG1: Amlodipine | 6 | cr-PWv | Complior | cr-PWv |
| al. 2014 |  |  | (71.4) | 54.9±4.5 | hypertension | IG2: CCB | (5mg daily) |  | cf-PWv | SP device | IG1: 9.9±1.1 |
|  |  |  | IG2: 21 | IG2: |  |  | IG2: Losartan |  | AIx |  | IG2: 9.5±1.4 |
|  |  |  | (71.4) | 54.0±6.9 |  |  | (100mg daily) |  |  |  |  |
|  |  |  |  |  |  |  |  |  |  |  | cf-PWv |
|  |  |  |  |  |  |  |  |  |  |  | IG1: 10.4±2.2 |
|  |  |  |  |  |  |  |  |  |  |  | IG2: 10.6±2.7 |
|  |  |  |  |  |  |  |  |  |  |  | AIx |
|  |  |  |  |  |  |  |  |  |  |  | IG1: 36.0±8.0 |
|  |  |  |  |  |  |  |  |  |  |  | IG2: 30.0±9.0 |
| Semenkin et | Russia | RCT | IG1: 20 | IG1: | Essential | IG1: ACEI + | IG1: Perindopril + | 24 | PWv | PulseTrace | IG1: 8.1±1.6 |
| al. 2014 |  |  | (60.0) | 51.9±7.6 | hypertension | Thiazide diuretics | Indapamide (5- |  |  |  | IG2: 7.2±1.3 |
|  |  |  | IG2: 20 | IG2: |  | IG2: ACEI + | 10mg/1.5mg daily) |  |  |  |  |
|  |  |  | (55.0) | 52.8±8.4 |  | Thiazide diuretics | IG2: Perindopril + |  |  |  |  |
|  |  |  |  |  |  |  | HCTZ (5- |  |  |  |  |
|  |  |  |  |  |  |  | 10mg/25mg daily) |  |  |  |  |

| **Reference** | **Country** | **Study design** | **Population characteristics** | | | **Intervention: Antihypertensive drugs** | | | **Outcome: Arterial stiffness** | | |
| --- | --- | --- | --- | --- | --- | --- | --- | --- | --- | --- | --- |
|  |  |  | **Sample size (n,**  **%female**  **)** | **Mean age (years)** | **Type of population** | **Pharmacological group** | **Drug (dose and frequency)** | **Length (weeks)** | **Index** | **Device** | **Basal levels** |
| Sumbria et | India | RCT | IG1: 50 | IG1: | Hypertension | IG1: Beta-blocker | IG1: Metoprolol | 36 | cf-PWv | Periscope | cf-PWv |
| al. 2014 |  |  | (30.9) | 45.0±10.6 |  | IG2: ARB | (25-50mg daily) |  |  |  | IG1: 10.7±3.7 |
|  |  |  | IG2: 50 | IG2: |  |  | IG2: Telmisartan |  |  |  | IG2: 10.4±4.7 |
|  |  |  | (23.5) | 45.0±10.3 |  |  | (20-40mg daily) |  |  |  |  |
| Hayek et al. | USA | Crossover | IG1:30 | 44.7±10.1 | Essential | IG1: Beta- | IG1: Nevibolol | 12 | cf-PWv | SphygmoCor | cf-PWv |
| 2015 |  | RCT | (57.0) |  | hypertension | blockers | (5mg daily) |  | AIx |  | 9.1±2.1 |
|  |  |  | IG2:30 |  |  | IG2: Beta- | IG2: Metoprolol |  |  |  |  |
|  |  |  | (57.0) |  |  | blockers | (50mg daily) |  |  |  | AIx |
|  |  |  |  |  |  |  |  |  |  |  | 23.0±8.9 |
| Pizoń et al. | Polonia | RCT | IG1: 19 | 53.1±13.0 | Hypertension | IG1: ACEI | IG1: Quinapril (20 | 4, 12 and | cf-PWv | Complior | IG1: 10.5±1.9 |
| 2015 |  |  | (NA) |  |  | IG2: CCB | mg daily) | 36 |  |  | IG2: 10.1±1.3 |
|  |  |  | IG2: 19 |  |  | IG3: Thiazide | IG2: Amlodipine |  |  |  | IG3: 10.8±1.7 |
|  |  |  | (NA) |  |  | diuretics | (5mg daily) |  |  |  | IG4: 10.8±1.6 |
|  |  |  | IG3: 19 |  |  | IG4: ARB | IG3: HCTZ (25mg |  |  |  | IG5: 10.5±1.5 |
|  |  |  | (NA) |  |  | IG5: Beta-blocker | daily) |  |  |  |  |
|  |  |  | IG4: 19 |  |  |  | IG4: Losartan |  |  |  |  |
|  |  |  | (NA) |  |  |  | (50mg daily) |  |  |  |  |
|  |  |  | IG5: 19 |  |  |  | IG5: Bisoprolol |  |  |  |  |
|  |  |  | (NA) |  |  |  | (5mg) |  |  |  |  |
| Posadzy- | Poland | RCT | IG1: 50 | 49-53 | Postmenopausal | IG1: ACEI | IG1: Perindopril | 48 | cf-PWv | Complior | IG1: 10.8±1.4 |
| Malaczynska |  |  | (100.0) |  | essential | IG2: Thiazide | (4mg daily) |  |  |  | IG2: 10.8±2.1 |
| et al. 2015 |  |  | IG2: |  | hypertension | diuretics | IG2: HCTZ (25mg |  |  |  |  |
|  |  |  | 50 |  | women |  | daily) |  |  |  |  |
|  |  |  | (100.0) |  |  |  |  |  |  |  |  |
| Rajzer et al. | Poland | RCT | IG1: 19 | IG1: | Essential | IG1: ACEI | IG1: Quinapril | 36 | cf-PWv | Complior | IG1: 10.5±2.2 |
| 2015 |  |  | (48.0) | 52.8±3 | hypertension | IG2: CCB | (20mg daily) |  |  |  | IG2: 10.1±1.3 |
|  |  |  | IG2: 19 | IG2: |  | IG3: Thiazide | IG2: Amlodipine |  |  |  | IG3: 10.8±1.3 |
|  |  |  | (48.0) | 53.4±2.8 |  | diuretics | (5mg daily) |  |  |  | IG4: 10.8±1.8 |
|  |  |  | IG3: 19 | IG3: |  | IG4: ARB | IG3: HCTZ (25mg |  |  |  | IG5: 10.5±1.3 |
|  |  |  | (42.0) | 51.4±2.7 |  | IG5: Beta-blocker | daily) |  |  |  |  |
|  |  |  | IG4: 19 | IG4: |  |  | IG4: Losartan |  |  |  |  |
|  |  |  | (48.0) | 53.5±3.2 |  |  | (50mg daily) |  |  |  |  |
|  |  |  | IG5: 19 | IG5: |  |  | IG5: Bisoprolol |  |  |  |  |
|  |  |  | (48.0) | 52.9±2.9 |  |  | (5mg daily) |  |  |  |  |

| **Reference** | **Country** | **Study design** | **Population characteristics** | | | **Intervention: Antihypertensive drugs** | | | **Outcome: Arterial stiffness** | | |
| --- | --- | --- | --- | --- | --- | --- | --- | --- | --- | --- | --- |
|  |  |  | **Sample size (n,**  **%female**  **)** | **Mean age (years)** | **Type of population** | **Pharmacological group** | **Drug (dose and frequency)** | **Length (weeks)** | **Index** | **Device** | **Basal levels** |
| Redon and | Spain | RCT | IG1: 41 | IG1: | DM type 2 and | IG1: ACEI + | IG1: Olmesartan + | 24 | cf-PWv | SphygmoCor | cf-PWv |
| Pichler 2016 |  |  | (34.1) | 58.7±8.3 | hypertension | CCB | Amlodipine |  | AIx |  | IG1: 8.9±1.8 |
|  |  |  | IG2: 47 | IG2: |  | IG2: ARB + CCB | (20mg/5mg daily) |  |  |  | IG2: 9.6±1.9 |
|  |  |  | (44.7) | 58.1±7.8 |  |  | IG2: Perindopril + |  |  |  |  |
|  |  |  |  |  |  |  | Amlodipine |  |  |  | AIx |
|  |  |  |  |  |  |  | (4mg/5mg daily) |  |  |  | IG1: |
|  |  |  |  |  |  |  |  |  |  |  | 30.2±11.5 |
|  |  |  |  |  |  |  |  |  |  |  | IG2: |
|  |  |  |  |  |  |  |  |  |  |  | 28.9±12.0 |
| Tsioufis et | Greece | RCT | IG1: 19 | IG1: | Essential | IG1: CCB + | IG1: Lercanidipine | 4 and 12 | cf-PWv | Complior | IG1: NA |
| al. 2016 |  |  | (42.0) | 54.0±10.0 | hypertension | ACEI | + Enalapril |  |  |  | IG2: NA |
|  |  |  | IG2: 18 | IG2: |  | IG2: CCB + | (10mg/20 mg daily) |  |  |  | IG3: NA |
|  |  |  | (50.0) | 58.0±9.0 |  | ACEI | IG2: Amlodipine + |  |  |  |  |
|  |  |  | IG3: 19 | IG3: |  | IG3: ACEI + | Enalapril |  |  |  |  |
|  |  |  | (47.0) | 58.0±10.0 |  | Thiazide diuretics | (5mg/20mg daily) |  |  |  |  |
|  |  |  |  |  |  |  | IG3: Enalapril + |  |  |  |  |
|  |  |  |  |  |  |  | HCTZ |  |  |  |  |
|  |  |  |  |  |  |  | (20mg/12.5mg |  |  |  |  |
|  |  |  |  |  |  |  | daily) |  |  |  |  |
| Nedogoba et | Russia | RCT | IG1: 20 | IG1: | Hypertension | IG1: ACEI + | IG1: NA | 24 | cf-PWv | Complior | cf-PW |
| al. 2017 |  |  | (35.0) | 50.3±8.7 |  | Diuretics | IG2: NA |  | AIx |  | IG1: 10.7±1.8 |
|  |  |  | IG2: 20 | IG2: |  | IG2: ACEI + | IG3: NA |  |  |  | IG2: 11.1±1.3 |
|  |  |  | (50.0) | 53.0±6.0 |  | CCB | IG4: NA |  |  |  | IG3: 10.4±1.6 |
|  |  |  | IG3: 20 | IG3: |  | IG3: ARB + |  |  |  |  | IG4: 11.3±2.0 |
|  |  |  | (50.0) | 47.6±11.6 |  | Diuretics |  |  |  |  |  |
|  |  |  | IG4: 20 | IG4: |  | IG4: ARB + CCB |  |  |  |  | AIx |
|  |  |  | (35.0) | 49.9±11.4 |  |  |  |  |  |  | IG1: 22.1±9.5 |
|  |  |  |  |  |  |  |  |  |  |  | IG2: 23.3±7.7 |
|  |  |  |  |  |  |  |  |  |  |  | IG3: |
|  |  |  |  |  |  |  |  |  |  |  | 22.4±10.2 |
|  |  |  |  |  |  |  |  |  |  |  | IG4: 24.8±8.6 |
| Williams et | United | RCT | IG1:192 | IG1: | Hypertension | IG1: ACEI + | IG1: Sacubitril + | 12 and 52 | cf-PWv | SphygmoCor | IG1: 10.3±2.1 |
| al. 2017 | Kingdom |  | (48.0) | 68.2±5.73 |  | ARB | Valsartan (200mg |  |  |  | IG2: 10.2±1.9 |
|  |  |  | IG2: 199 | IG2: |  | IG2: ARB | daily) |  |  |  |  |
|  |  |  | (47.6) | 67.2±5.9 |  |  | IG2: Olmesartan |  |  |  |  |
|  |  |  |  |  |  |  | (20mg daily) |  |  |  |  |

| **Reference** | **Country** | **Study design** | **Population characteristics** | | | **Intervention: Antihypertensive drugs** | | | **Outcome: Arterial stiffness** | | |
| --- | --- | --- | --- | --- | --- | --- | --- | --- | --- | --- | --- |
|  |  |  | **Sample size (n,**  **%female**  **)** | **Mean age (years)** | **Type of population** | **Pharmacological group** | **Drug (dose and frequency)** | **Length (weeks)** | **Index** | **Device** | **Basal levels** |
| Acetto et al. | Multinational | RCT | IG1: 9 | IG1: | Essential | IG1: ARB | IG1: Valsartan (80 | 16 | cf-PWv | SphygmoCor | cf-PWv |
| 2018 | (Slovenia, |  | (51.0) | 49.9±3.3 | hypertension | IG2: ARB + | mg daily) |  | AIx |  | IG1: 9.2±2.3 |
|  | Czech |  | IG2: 30 | IG2: |  | Thiazide diuretics | IG2: Valsartan + |  |  |  | IG2: 11.9±2.1 |
|  | Republic, |  | (47.0) | 53.2±2.5 |  |  | HCTZ |  |  |  |  |
|  | Croatia, Russia, |  |  |  |  |  | (160mg/12.5mg |  |  |  | AIx |
|  | Ukraine and |  |  |  |  |  | daily) |  |  |  | IG1: |
|  | Russian |  |  |  |  |  |  |  |  |  | 16.5±16.0 |
|  | Federation) |  |  |  |  |  |  |  |  |  | IG2: 25.0±8.9 |
| Jekell and | Sweden | RCT | IG1:26 | IG1: | Hypertension | IG1: AARA | IG1: Doxazosin | 12 | a-PWv | Arteriograph | a-PWv |
| Kahan 2018 |  |  | (27.0) | 53.5±11.3 |  | IG2: ACEI | (4mg daily) |  | AIx |  | IG1: 8.9±1.6 |
|  |  |  | IG2: 32 | IG2: |  |  | IG2: Ramipril (5mg |  |  |  | IG2: 9.0±2.6 |
|  |  |  | (38.0) | 53.7±13.3 |  |  | daily) |  |  |  |  |
|  |  |  |  |  |  |  |  |  |  |  | AIx |
|  |  |  |  |  |  |  |  |  |  |  | IG1: |
|  |  |  |  |  |  |  |  |  |  |  | 43.8±11.6 |
|  |  |  |  |  |  |  |  |  |  |  | IG2: |
|  |  |  |  |  |  |  |  |  |  |  | 44.1±14.9 |
| Dudinskaya | Russia | RCT | IG1: 57 | IG1: | Hypertension and | IG1: Direct- | IG1: Moxonidine | 48 | cf-PWv | SphygmoCor | IG1: 10.4±2.6 |
| et al. 2021 |  |  | (100.0) | 63.0±9.0 | osteopenia | acting vasodilator | (0.4mg daily) |  |  |  | IG2: 10.4±2.5 |
|  |  |  | IG2: 57 | IG2: |  | IG2: Beta- | IG2: Bisoprolol |  |  |  |  |
|  |  |  | (100.0) | 61.6±9.0 |  | blockers | (5mg daily) |  |  |  |  |
| Shisko et al. | Russia | RCT | IG1: 22 | IG1: | Hypertension | IG1: ACEI + | IG1: Lisinopril + | 8 | cr-PWv | Ipecard-M | IG1: 10.3 |
| 2022 |  |  | (31.8) | 47±33.5 |  | Thiazide diuretics | HCTZ (10mg/25mg |  |  |  | (8.7-11.2) |
|  |  |  | IG2: 18 | IG2: |  | IG2: ACEI + | daily) |  |  |  | IG2: 10.5 |
|  |  |  | (38.9) | 48±39.5 |  | Thiazide diuretics | IG2: Lisinopril + |  |  |  | (8.4-11.2) |
|  |  |  |  |  |  |  | Chlorthalidone |  |  |  |  |
|  |  |  |  |  |  |  | (10mg/25mg daily) |  |  |  |  |
| Data are shown as mean ± standard deviation (SD) or median (interquartile range); AAD, antialdesterone diuretics; AAIx, aortic augmentation index; AARA, alpha-adrenergic receptor antagonists; ACEI, angiotensin-convertingn enzyme inhibitors; AHA, American Heart asociation; AIx, augmentation index; a-PWv: aortic pulse wave velocity; ARB, angiotensin receptor blockers; ba-PWv, brachial-ankle pulse wave velocity; br-PWv, brachial-radial pulse wave velocity; CAIx, carotid augmentation index; CCB, calcium channel blockers; cf-PWv, carotid-femoral pulse wave velocity; cr-PWv, carotid-radial pulse wave  velocity; CVD, cardiovascular disease; DM, diabetes mellitus; ff-PWv, foot-foot pulse wave velocity; ft-PWv, femoral-tibial pulse wave velocity; HCTZ, hydrochlorothiazithe; IG, intervention group; IGT, impaired glucose tolerance; NA, not available; RCT, randomized controlled trial; TD: thiazide diuretics; UK, United Kingdon; USA, United States of America. | | | | | | | | | | | |

**Table S4.** Quality grading of evidence for pulse wave velocity.

| **Certainty asessment** | | | | |  |  | **№ of patients** | |  | **Effect** |  | **Certainty** | **Importance** |
| --- | --- | --- | --- | --- | --- | --- | --- | --- | --- | --- | --- | --- | --- |
| **№ of studies** | **Study design** | **Risk of bias** | **Inconsistency** | **Indirectness** | **Imprecision** | **Other considerations** | **Treatment** | **Treatment+Placebo** | | **Relative (95% CI)** | **Absolute (95% CI)** |  |  |
| ACEI-ACEI/ARB | | | | | | | | | | | | | |
| **2** | Randomised trials | Serious^a^ | Serious^b^ | Not serious | Not serious | None | 23 |  | 22 | - | SMD 1.76  SD lower (0.48 lower a  3.04 lower) | ⨁⨁◯◯  Low | IMPORTANT |
| ACEI-AARA |  |  |  |  |  |  |  |  |  |  |  |  |  |
| **1** | Randomised trials | Very serious^c^ | - | Not serious | Not serious | None | 32 |  | 26 | - | SMD 0.15  SD higher (0.35 lower a  0.66 higher) | ⨁⨁◯◯  Low | NOT IMPORTANT |
| ACEI-ARB |  |  |  |  |  |  |  |  |  |  |  |  |  |
| **12** | Randomised trials | Serious^a^ | Very serious^d^ | Not serious | Not serious | None | 252 |  | 250 | - | SMD 0.06  SD lower (0.48 lower a  0.35 higher) | ⨁◯◯◯  Very low | NOT IMPORTANT |
| ACEI-CCB |  |  |  |  |  |  |  |  |  |  |  |  |  |
| **9** | Randomised trials | Very serious^c^ | Not serious | Not serious | Not serious | None | 176 |  | 163 | - | SMD 0.2 SD  higher (0.04 lower a  0.44 higher) | ⨁⨁◯◯  Low | NOT IMPORTANT |
| ACEI-PB |  |  |  |  |  |  |  |  |  |  |  |  |  |
| **4** | Randomised trials | Serious^a^ | Very serious^d^ | Not serious | Not serious | None | 65 |  | 61 | - | SMD 1.15  SD lower (2.13 lower a  0.18 lower) | ⨁◯◯◯  Very low | CRITICAL |
| ACEI-RI |  |  |  |  |  |  |  |  |  |  |  |  |  |
| **2** | Randomised trials | Serious^a^ | Not serious | Not serious | Not serious | None | 45 |  | 43 | - | SMD 0.03  SD higher (0.39 lower a  0.45 higher) | ⨁⨁⨁◯  Moderate | NOT IMPORTANT |
| ACEI/CCB-ARB/CCB | | | | | | | | | | | | | |
| **2** | Randomised trials | Serious^a^ | Not serious | Not serious | Not serious | None | 61 |  | 67 | - | SMD 0.27  SD lower (0.62 lower a  0.08 higher) | ⨁⨁⨁◯  Moderate | NOT IMPORTANT |
| ARB-ACEI/ARB | | | | | | | | | | | | | |
| **3** | Randomised trials | Serious^a^ | Very serious^d^ | Not serious | Not serious | None | 221 |  | 214 | - | SMD 1.06  SD lower (2.49 lower a  0.36 higher) | ⨁◯◯◯  Very low | NOT IMPORTANT |
| ARB-CCB |  |  |  |  |  |  |  |  |  |  |  |  |  |
| **14** | Randomised trials | Very serious^c^ | Serious^b^ | Not serious | Not serious | Publication bias^e^ | 477 |  | 475 | - | SMD 0.3 SD  higher (0.06 higher  a 0.55  higher) | ⨁◯◯◯  Very low | IMPORTANT |
| ARB-TD/ARB |  |  |  |  |  |  |  |  |  |  |  |  |  |
| **1** | Randomised trials | Very serious^c^ | - | Not serious | Not serious | None | 9 |  | 30 | - | SMD 0.34  SD lower (1.09 lower a  0.41 higher) | ⨁⨁◯◯  Low | NOT IMPORTANT |
| ARB-PB |  |  |  |  |  |  |  |  |  |  |  |  |  |
| **1** | Randomised trials | Serious^a^ | - | Not serious | Not serious | None | 33 |  | 35 | - | SMD 0.01  SD higher | ⨁⨁⨁◯  Moderate | NOT IMPORTANT |

|  |  |  |  |  |  |  |  |  |  | (0.47 lower a  0.49 higher) |  |  |
| --- | --- | --- | --- | --- | --- | --- | --- | --- | --- | --- | --- | --- |
| ARB-RI |  |  |  |  |  |  |  |  |  |  |  |  |
| **1** | Randomised trials | Serious^a^ | - | Not serious | Not serious | None | 14 | 15 | - | SMD 0.5 SD  higher (0.03 lower a  1.02 higher) | ⨁⨁⨁◯  Moderate | NOT IMPORTANT |
| BB-ACEI |  |  |  |  |  |  |  |  |  |  |  |  |
| **9** | Randomised trials | Very serious^c^ | Not serious | Not serious | Not serious | None | 146 | 155 | - | SMD 0.24  SD lower (0.47 lower a  0.01 lower) | ⨁⨁◯◯  Low | IMPORTANT |
| BB-ARB |  |  |  |  |  |  |  |  |  |  |  |  |
| **6** | Randomised trials | Serious^a^ | Not serious | Not serious | Serious^f^ | None | 230 | 226 | - | SMD 0.12  SD higher (0.07 lower a  0.3 higher) | ⨁⨁◯◯  Low | NOT IMPORTANT |
| BB-DAV |  |  |  |  |  |  |  |  |  |  |  |  |
| **1** | Randomised trials | Serious^a^ | - | Not serious | Not serious | None | 57 | 57 | - | SMD 0.34  SD lower (0.71 lower a  0.03 higher) | ⨁⨁⨁◯  Moderate | NOT IMPORTANT |
| BB-CCB |  |  |  |  |  |  |  |  |  |  |  |  |
| **4** | Randomised trials | Serious^a^ | Serious^g^ | Not serious | Serious^f^ | None | 69 | 66 | - | SMD 0.05  SD lower (0.51 lower a  0.41 higher) | ⨁◯◯◯  Very low | NOT IMPORTANT |
| BB-TD |  |  |  |  |  |  |  |  |  |  |  |  |
| **3** | Randomised trials | Serious^a^ | Not serious | Not serious | Not serious | None | 55 | 51 | - | SMD 0.09  SD higher (0.3 lower a  0.47 higher) | ⨁⨁⨁◯  Moderate | NOT IMPORTANT |
| BB-TD/ACEI | | | | | | | | | | | | |
| **3** | Randomised trials | Serious^a^ | Not serious | Not serious | Not serious | None | 510 | 509 | - | SMD 0.02  SD higher (0.15 lower a  0.2 higher) | ⨁⨁⨁◯  Moderate | NOT IMPORTANT |
| BB-RI |  |  |  |  |  |  |  |  |  |  |  |  |
| **2** | Randomised trials | Serious^a^ | Not serious | Not serious | Serious^f^ | None | 34 | 36 | - | SMD 0.46  SD lower (0.93 lower a  0.02 higher) | ⨁⨁◯◯  Low | NOT IMPORTANT |
| BB/TD-ARB/TD | | | | | | | | | | | | |
| **2** | Randomised trials | Serious^a^ | Serious^g^ | Not serious | Not serious | None | 58 | 66 | - | SMD 0.49  SD lower (1.31 lower a  0.34 higher) | ⨁⨁◯◯  Low | NOT IMPORTANT |
| CCB-PB |  |  |  |  |  |  |  |  |  |  |  |  |
| **2** | Randomised trials | Serious^a^ | Not serious | Not serious | Not serious | None | 41 | 44 | - | SMD 0.36  SD lower (0.72 lower a  0.01 higher) | ⨁⨁⨁◯  Moderate | NOT IMPORTANT |
| TD-ACEI |  |  |  |  |  |  |  |  |  |  |  |  |
| **5** | Randomised trials | Serious^a^ | Not serious | Not serious | Not serious | None | 109 | 112 | - | SMD 0.3 SD  lower (0.65 lower a  0.05 higher) | ⨁⨁⨁◯  Moderate | NOT IMPORTANT |
| TD-ARB |  |  |  |  |  |  |  |  |  |  |  |  |
| **3** | Randomised trials | Serious^a^ | Not serious | Not serious | Not serious | None | 82 | 71 | - | SMD 0.04  SD lower | ⨁⨁⨁◯  Moderate | NOT IMPORTANT |

|  |  |  |  |  |  |  |  |  |  | (0.35 lower a  0.28 higher) |  |  |
| --- | --- | --- | --- | --- | --- | --- | --- | --- | --- | --- | --- | --- |
| TD-CCB |  |  |  |  |  |  |  |  |  |  |  |  |
| **5** | Randomised trials | Serious^a^ | Not serious | Not serious | Serious^f^ | Publication bias^e^ | 111 | 101 | - | SMD 0.17  SD lower (0.41 lower a  0.07 higher) | ⨁◯◯◯  Very low | NOT IMPORTANT |
| TD-PB |  |  |  |  |  |  |  |  |  |  |  |  |
| **1** | Randomised trials | Serious^a^ | - | Not serious | Serious^f^ | None | 44 | 35 | - | SMD 0.07  SD higher (0.38 lower a  0.51 higher) | ⨁⨁◯◯  Low | NOT IMPORTANT |
| TD/ACEI-ACEI/CCB | | | | | | | | | | | | |
| **3** | Randomised trials | Serious^a^ | Not serious | Not serious | Not serious | Publication bias^e^ | 65 | 64 | - | SMD 0.35  SD higher (0.07 lower a  0.78 higher) | ⨁⨁◯◯  Low | IMPORTANT |
| TD/ARB-ARB/CCB | | | | | | | | | | | | |
| **1** | Randomised trials | Serious^a^ | Not serious | Not serious | Not serious | None | 104 | 133 | - | SMD 0.27  SD lower (0.54 lower a  0.00 higher) | ⨁⨁⨁◯  Moderate | NOT IMPORTANT |
| BB-PB |  |  |  |  |  |  |  |  |  |  |  |  |
| **2** | Randomised trials | Serious^a^ | Not serious | Not serious | Not serious | None | 32 | 32 | - | SMD 0.48  SD lower (0.98 lower a  0.02 higher) | ⨁⨁⨁◯  Moderate | NOT IMPORTANT |
| TD/ARB-CCB | | | | | | | | | | | | |
| **1** | Randomised trials | Not serious | - | Not serious | Not serious | None | 73 | 71 | - | SMD 0.33  SD lower (0.68 lower a  0.01 higher) | ⨁⨁⨁⨁  High | NOT IMPORTANT |
| AAD-PB |  |  |  |  |  |  |  |  |  |  |  |  |
| **1** | Randomised trials | Serious^a^ | - | Not serious | Not serious | None | 58 | 52 | - | SMD 0.39  SD lower (0.76 lower a  0.02 lower) | ⨁⨁⨁◯  Moderate | IMPORTANT |
| AAD-CCB |  |  |  |  |  |  |  |  |  |  |  |  |
| **1** | Randomised trials | Serious^a^ | - | Not serious | Not serious | None | 134 | 135 | - | SMD 0.020  SD higher (0.13 lower a  0.54 higher) | ⨁⨁⨁◯  Moderate | NOT IMPORTANT |

AAD: antialdosterone diuretics; AARA: Alpha-adrenergic receptor antagonists; ACEI: Angiotensin-converting enzyme inhibitors; ARB: Angiotensin receptor blockers; BB: beta-blockers; CCB: Calcium channel blockers; CI: Confidence intervals; DAV: direct-acting vasodilators; PB: placebo; RI: renin inhibitors; SMD: Standardized mean difference; TD: thiazide diuretics. a. Some concerns risk of bias; b. Substantial heterogeneity; c. High risk of bias; d. Considerable heterogeneity; e. Publication bias; f. The direction of the effect is controversial; g. Moderate heterogeneity.

**Table S5.** Quality grading of evidence for augmentation index.

| **Certainty asessment** | | | | |  |  | **№ of patients** | |  | **Effect** |  | **Certainty** | **Importance** |
| --- | --- | --- | --- | --- | --- | --- | --- | --- | --- | --- | --- | --- | --- |
| **№ of studies** | **Study design** | **Risk of bias** | **Inconsistency** | **Indirectness** | **Imprecision** | **Other considerations** | **Treatment** | **Treatment+Placebo** | | **Relative (95% CI)** | **Absolute (95% CI)** |  |  |
| ACEI-AARA | | | | | | | | | | | | | |
| **1** | Randomised trials | Very serious^a^ | - | Not serious | Not serious | None | 32 |  | 26 | - | SMD 0.21 SD  lower  (-0.71 lower a 0.30 higher) | ⨁⨁◯◯  Low | NOT IMPORTANT |
| ACEI-ARB |  |  |  |  |  |  |  |  |  |  |  |  |  |
| **5** | Randomised trials | Serious^b^ | Very serious^c^ | Not serious | Not serious | Publication bias^d^ | 150 |  | 150 | - | SMD 0.39 SD  lower  (0.19 lower a 0.96 higher) | ⨁◯◯◯  Very low | NOT IMPORTANT |
| ACEI-CCB |  |  |  |  |  |  |  |  |  |  |  |  |  |
| **3** | Randomised trials | Very serious^a^ | Not serious | Not serious | Serious^e^ | None | 40 |  | 48 | - | SMD 0.15 SD  higher  (0.28 lower a 0.59 higher) | ⨁◯◯◯  Very low | NOT IMPORTANT |
| ACEI-PB |  |  |  |  |  |  |  |  |  |  |  |  |  |
| **5** | Randomised trials | Serious^b^ | Very serious^c^ | Not serious | Not serious | None | 146 |  | 151 | - | SMD 1.32 SD  lower  (2.42 lower a 0.22 lower) | ⨁◯◯◯  Very low | CRITICAL |
| ACEI-RI |  |  |  |  |  |  |  |  |  |  |  |  |  |
| **2** | Randomised trials | Serious^b^ | Not serious | Not serious | Not serious | None | 45 |  | 43 | - | SMD 0.14 SD  higher  (0.21 lower a 0.49 higher) | ⨁⨁⨁◯  Moderate | NOT IMPORTANT |
| ACEI/CCB-ARB/CCB | | | | | | | | | | | | | |
| **2** | Randomised trials | Serious^b^ | Serious^f^ | Not serious | Not serious | None | 61 |  | 67 | - | SMD 0.14 SD  higher  (0.30 lower a 0.57 higher) | ⨁⨁◯◯  Low | NOT IMPORTANT |
| ARB-CCB |  |  |  |  |  |  |  |  |  |  |  |  |  |
| **2** | Randomised trials | Very serious^a^ | Not serious | Not serious | Not serious | None | 132 |  | 134 | - | SMD 0.02 SD  higher  (0.22 lower a 0.26 higher) | ⨁⨁◯◯  Low | NOT IMPORTANT |
| ARB-TD/ARB | | | | | | | | | | | | | |
| **1** | Randomised trials | Very serious^a^ | - | Not serious | Serious^e^ | None | 9 |  | 30 | - | SMD 0.45 SD  higher  (0.31 lower a 1.20 higher) | ⨁◯◯◯  Very low | NOT IMPORTANT |
| ARB-PB |  |  |  |  |  |  |  |  |  |  |  |  |  |
| **2** | Randomised trials | Serious^b^ | Very serious^c^ | Not serious | Not serious | None | 53 |  | 55 | - | SMD 1.00 SD  lower (2.61 lower a 0.6  higher) | ⨁◯◯◯  Very low | NOT IMPORTANT |
| ARB-RI |  |  |  |  |  |  |  |  |  |  |  |  |  |
| **1** | Randomised trials | Serious^b^ | - | Not serious | Not serious | None | 14 |  | 15 | - | SMD 0.29 SD  higher  (0.44 lower a 1.02 higher) | ⨁⨁⨁◯  Moderate | NOT IMPORTANT |
| BB-ACEI |  |  |  |  |  |  |  |  |  |  |  |  |  |
| **6** | Randomised trials | Very serious^a^ | Very serious^c^ | Not serious | Not serious | None | 110 |  | 127 | - | SMD 0.28 SD  higher  (0.30 lower a 0.87 higher) | ⨁◯◯◯  Very low | NOT IMPORTANT |
| BB-ARB |  |  |  |  |  |  |  |  |  |  |  |  |  |

| **2** | Randomised trials | Serious^b^ | Serious^f^ | Not serious | Not serious | None | 175 | 163 | - | SMD 0.36 SD  higher  (-0.00 a 0.73  higher) | ⨁⨁◯◯  Low | NOT IMPORTANT |
| --- | --- | --- | --- | --- | --- | --- | --- | --- | --- | --- | --- | --- |
| BB-CCB |  |  |  |  |  |  |  |  |  |  |  |  |
| **2** | Randomised trials | Serious^b^ | Very serious^c^ | Not serious | Not serious | None | 28 | 38 | - | SMD 1.20 SD  higher  (0.66 higher a 1.75 higher) | ⨁◯◯◯  Very low | NOT IMPORTANT |
| BB-TD |  |  |  |  |  |  |  |  |  |  |  |  |
| **1** | Randomised trials | Serious^b^ | - | Not serious | Not serious | None | 17 | 14 | - | SMD 0.47 SD  higher  (0.26 lower a 1.20 higher) | ⨁⨁◯◯  Low | NOT IMPORTANT |
| BB-TD/ACEI | | | | | | | | | | | | |
| **4** | Randomised trials | Serious^b^ | Serious^f^ | Not serious | Not serious | None | 532 | 539 | - | SMD 0.39 SD  higher  (0.19 higher a 0.58 higher) | ⨁⨁◯◯  Low | IMPORTANT |
| BB-PB |  |  |  |  |  |  |  |  |  |  |  |  |
| **2** | Randomised trials | Serious^b^ | Not serious | Not serious | Not serious | None | 32 | 32 | - | SMD 0.73 SD  higher  (0.22 higher a 1.24 higher) | ⨁⨁◯◯  Low | NOT IMPORTANT |
| BB-RI |  |  |  |  |  |  |  |  |  |  |  |  |
| **2** | Randomised trials | Serious^b^ | Serious^f^ | Not serious | Serious^e^ | None | 34 | 36 | - | SMD 0.35 SD  lower  (1.03 lower a 0.34 higher) | ⨁◯◯◯  Very low | NOT IMPORTANT |
| BB/TD-TD/ARB | | | | | | | | | | | | |
| **2** | Randomised trials | Serious^b^ | Serious^f^ | Not serious | Not serious | None | 58 | 66 | - | SMD 0.9 SD higher (0.18 higher a 1.71  higher) | ⨁⨁◯◯  Low | NOT IMPORTANT |
| CCB-TD/ACEI | | | | | | | | | | | | |
| **1** | Randomised trials | Serious^b^ | - | Not serious | Not serious | None | 28 | 28 | - | SMD 1.33 SD  higher  (0.75 higher a 1.91 higer) | ⨁⨁⨁◯  Moderate | NOT IMPORTANT |
| CCB-PB |  |  |  |  |  |  |  |  |  |  |  |  |
| **1** | Randomised trials | Serious^b^ | - | Not serious | Not serious | None | 33 | 55 | - | SMD 0.25 SD  lower  (0.73 lower a 0.23 higher) | ⨁⨁⨁◯  Moderate | NOT IMPORTANT |
| TD-ACEI |  |  |  |  |  |  |  |  |  |  |  |  |
| **2** | Randomised trials | Serious^b^ | Not serious | Not serious | Not serious | None | 63 | 66 | - | SMD 0.37 SD  higher  (0.02 higher a 0.72 higher) | ⨁⨁⨁◯  Moderate | NOT IMPORTANT |
| TD-ARB |  |  |  |  |  |  |  |  |  |  |  |  |
| **2** | Randomised trials | Serious^b^ | Very serious^c^ | Not serious | Not serious | None | 64 | 53 | - | SMD 0.83 SD  higher  (0.86 lower a 2.51 higher) | ⨁◯◯◯  Very low | NOT IMPORTANT |
| TD-CCB |  |  |  |  |  |  |  |  |  |  |  |  |
| **3** | Randomised trials | Serious^b^ | Serious^f^ | Not serious | Serious^e^ | None | 85 | 75 | - | SMD 0.09 SD  lower  (0.65 lower a 0.46  higher) | ⨁◯◯◯  Very low | NOT IMPORTANT |
| TD-TD/ACEI | | | | | | | | | | | | |
| **1** | Randomised trials | Serious^b^ | - | Not serious | Not serious | None | 28 | 28 | - | SMD 1.02 SD  higher | ⨁⨁⨁◯  Moderate | NOT IMPORTANT |

|  |  |  |  |  |  |  |  |  |  | (0.46 higher a 1.58 higher) |  |  |
| --- | --- | --- | --- | --- | --- | --- | --- | --- | --- | --- | --- | --- |
| TD-PB |  |  |  |  |  |  |  |  |  |  |  |  |
| **2** | Randomised trials | Serious^b^ | Not serious | Not serious | Not serious | None | 64 | 55 | - | SMD 0.24 SD  lower  (0.60 lower a 0.12 higher) | ⨁⨁⨁◯  Moderate | NOT IMPORTANT |
| TD/ACEI-ACEI/CCB | | | | | | | | | | | | |
| **1** | Randomised trials | Serious^b^ | - | Not serious | Not serious | None | 20 | 20 | - | SMD 0.14 SD  higher  (0.48 lower a 0.76 higher) | ⨁⨁⨁◯  Moderate | NOT IMPORTANT |
| TD/ACEI-ARB/CCB | | | | | | | | | | | | |
| **1** | Randomised trials | Serious^b^ | - | Not serious | Serious^e^ | None | 20 | 20 | - | SMD 0.01 SD  lower  (0.63 lower a 0.61 higher) | ⨁⨁◯◯  Low | NOT IMPORTANT |
| TD/ACEI-TD/ARB | | | | | | | | | | | | |
| **1** | Randomised trials | Serious^b^ | - | Not serious | Not serious | None | 20 | 20 | - | SMD 0.02 SD  lower  (0.64 lower a 0.60 higher) | ⨁⨁⨁◯  Moderate | NOT IMPORTANT |
| TD/ARB-ACEI/CCB | | | | | | | | | | | | |
| **1** | Randomised trials | Serious^b^ | - | Not serious | Not serious | None | 20 | 20 | - | SMD 0.14 SD  lower  (0.48 lower a 0.76  higher) | ⨁⨁⨁◯  Moderate | NOT IMPORTANT |
| TD/ARB-ARB/CCB | | | | | | | | | | | | |
| **2** | Randomised trials | Serious^b^ | Not serious | Not serious | Not serious | None | 124 | 123 | - | SMD 0.16 SD  higher  (0.03 lower a 0.34 higher) | ⨁⨁⨁◯  Moderate | NOT IMPORTANT |
| CCB-TD/ARB | | | | | | | | | | | | |
| **1** | Randomised trials | Not serious | - | Not serious | Not serious | None | 71 | 73 | - | SMD 0.06 SD  higher  (0.28 lower a 0.40 higher) | ⨁⨁⨁⨁  High | NOT IMPORTANT |

AARA: Alpha-adrenergic receptor antagonists; ACEI: Angiotensin-converting enzyme inhibitors; ARB: Angiotensin receptor blockers; BB: beta-blockers; CCB: Calcium channel blockers; CI: Confidence intervals; PB: placebo; RI: renin inhibitors; SMD: Standardized mean difference; TD: thiazide diuretics. a. High risk of bias; b. Some concerns risk of bias; c. Considerable heterogeneity; d. Publication bias ; e. The direction of the effect is controversial; f. Moderate heterogeneity; g. Substantial heterogeneity.

**Table S6.** Effectiveness ranking of different types of antihypertensive drugs on pulse wave velocity.

|  | **Rank statistics** | **Probabilities** | |
| --- | --- | --- | --- |
|  | **Mean** | **Best** | **SUCRA** |
| **PB** | 15.1 | 0.00 | 0.06 |
| **BB** | 9.8 | 0.00 | 0.41 |
| **TD** | 10.9 | 0.00 | 0.34 |
| **AAD** | 9.4 | 0.01 | 0.44 |
| **ACEI** | 6.4 | 0.00 | 0.64 |
| **ARB** | 8.1 | 0.00 | 0.53 |
| **CCB** | 12.8 | 0.00 | 0.22 |
| **RI** | 10.0 | 0.00 | 0.40 |
| **AARA** | 11.3 | 0.02 | 0.32 |
| **DAV** | 5.1 | 0.19 | 0.73 |
| **BB/TD** | 9.2 | 0.03 | 0.45 |
| **TD/ACEI** | 8.6 | 0.00 | 0.50 |
| **TD/ARB** | 6.8 | 0.01 | 0.62 |
| **ACEI/ARB** | 2.0 | 0.53 | 0.93 |
| **ACEI/CCB** | 6.1 | 0.05 | 0.66 |
| **ARB/CCB** | 4.3 | 0.16 | 0.78 |

AAD: antialdosterone diuretics; AARA: Alpha-adrenergic receptor antagonists; ACEI: Angiotensin- converting enzyme inhibitors; ARB: Angiotensin receptor blockers; BB: beta-blockers; CCB: Calcium channel blockers; DAV: direct-acting vasodilators; PB: placebo; RI: renin inhibitors; TD: thiazide diuretics.

**Table S7.** Effectiveness ranking of different types of antihypertensive drugs on augmentation index.

|  | **Rank statistics** | **Probabilities** | |
| --- | --- | --- | --- |
|  | **Mean** | **Best** | **SUCRA** |
| **PB** | 11.4 | 0.00 | 0.13 |
| **BB** | 11.9 | 0.00 | 0.09 |
| **TD** | 9.3 | 0.00 | 0.31 |
| **ACEI** | 4.2 | 0.08 | 0.73 |
| **ARB** | 7.0 | 0.01 | 0.50 |
| **CCB** | 6.4 | 0.01 | 0.55 |
| **RI** | 5.0 | 0.14 | 0.67 |
| **AARA** | 8.4 | 0.01 | 0.39 |
| **BB/TD** | 10.1 | 0.02 | 0.24 |
| **TD/ACEI** | 5.1 | 0.06 | 0.66 |
| **TD/ARB** | 5.6 | 0.04 | 0.62 |
| **ACEI/CCB** | 3.7 | 0.28 | 0.77 |
| **ARB/CCB** | 3.0 | 0.35 | 0.83 |

AARA: Alpha-adrenergic receptor antagonists; ACEI: Angiotensin-converting enzyme inhibitors; ARB: Angiotensin receptor blockers; BB: beta-blockers; CCB: Calcium channel blockers; PB: placebo; RI: renin inhibitors; TD: thiazide diuretics.

**Table S8.** Pooled mean differences of different types of antihypertensive drugs on central pulse wave velocity.

| PB | -0.48  (-0.98, 0.02) | 0.07  (-0.38, 0.51) | **-0.39**  **(-0.76, -**  **0.02)** | -1.09  (-2.34, 0.16) | 0.01  (-0.47, 0.49) | -0.46  (-1.14, 0.23) | NA | NA | NA | NA | NA | NA | NA | NA | NA |
| --- | --- | --- | --- | --- | --- | --- | --- | --- | --- | --- | --- | --- | --- | --- | --- |
| **-0.51**  **(-0.99, -0.03)** | BB | -0.09  (-0.47, 0.30) | NA | 0.22  (-0.03, 0.47) | -0.12  (-0.30, 0.07) | -0.16  (-0.54, 0.22) | 0.46  (-0.01, 0.94) | NA | 0.34  (-0.03, 0.71) | -0.02  (-0.20, 0.15) | NA | NA | NA | NA | NA |
| -0.46  (-0.96, 0.03) | 0.05  (-0.36, 0.45) | TD | NA | 0.30  (-0.05, 0.65) | 0.04  (-0.28, 0.35) | 0.11  (-0.16, 0.38) | NA | NA | NA | NA | NA | NA | NA | NA | NA |
| -0.51  (-1.32, 0.29) | -0.00  (-0.86, 0.85) | -0.05  (-0.91, 0.81) | AAD | NA | NA | -0.20  (-0.54, 0.14) | NA | NA | NA | NA | NA | NA | NA | **NA** |  |
| **-0.85** | -0.34 | **-0.38** | -0.33 | ACEI | -0.26 | -0.19 | -0.03 | -0.15 | NA | NA | NA | NA | NA | **2.44** | NA |
| **(-1.29, -0.40)** | (-0.69, 0.02) | **(-0.76, -** | (-1.17, 0.51) |  | (-0.77, 0.26) | (-0.50, 0.12) | (-0.45, 0.39) | (-0.66, 0.35) |  |  |  |  |  | **(1.38,** |  |
|  |  | **0.01)** |  |  |  |  |  |  |  |  |  |  |  | **3.50)** |  |
| **-0.48** | 0.03 | -0.02 | 0.03 | **0.36** | ARB | 0.01 | -0.73 | NA | NA | NA | NA | 0.34 | NA | 1.16 | NA |
| **(-0.95, -0.02)** | (-0.32, 0.37) | (-0.40, 0.35) | (-0.81, 0.87) | **(0.06, 0.66)** |  | (-0.18, 0.19) | (-1.49, 0.03) |  |  |  |  | (-0.41, |  | (-1.23, |  |
|  |  |  |  |  |  |  |  |  |  |  |  | 1.09) |  | 3.55) |  |
| -0.33 | 0.18 | 0.13 | 0.18 | **0.51** | 0.15 | CCB | NA | NA | NA | NA | NA | -0.33 | NA | NA | NA |
| (-0.79, 0.13) | (-0.20, 0.55) | (-0.25, 0.50) | (-0.62, 0.99) | **(0.17, 0.85)** | (-0.19, 0.49) |  |  |  |  |  |  | (-0.68, |  |  |  |
|  |  |  |  |  |  |  |  |  |  |  |  | 0.01) |  |  |  |
| -0.66 | -0.15 | -0.20 | -0.15 | 0.19 | -0.18 | -0.33 | RI | NA | NA | NA | NA | NA | NA | NA | NA |
| (-1.54, 0.22) | (-0.99, 0.68) | (-1.05, 0.65) | (-1.28, 0.99) | (-0.59, 0.96) | (-0.98, 0.62) | (-1.16, 0.51) |  |  |  |  |  |  |  |  |  |
| -0.72 | -0.21 | -0.25 | -0.20 | 0.13 | -0.23 | -0.38 | -0.06 | AARA | NA | NA | NA | NA | NA | NA | NA |
| (-2.00, 0.56) | (-1.46, 1.05) | (-1.52, 1.01) | (-1.67, 1.27) | (-1.07, 1.33) | (-1.47, 1.01) | (-1.63, 0.87) | (-1.49, 1.36) |  |  |  |  |  |  |  |  |
| -0.98 | -0.47 | -0.52 | -0.47 | -0.13 | -0.50 | -0.65 | -0.32 | -0.26 | DAV | NA | NA | NA | NA | NA | NA |
| (-2.18, 0.22) | (-1.57, 0.63) | (-1.69, 0.65) | (-1.86, 0.93) | (-1.29, 1.02) | (-1.65, 0.66) | (-1.81, 0.52) | (-1.70, 1.06) | (-1.93, 1.41) |  |  |  |  |  |  |  |
| -0.49 | 0.02 | -0.03 | 0.02 | 0.35 | -0.01 | -0.16 | 0.17 | 0.22 | 0.49 | TD/ACEI | -0.45 | -0.14 | -0.04 | NA | NA |
| (-1.22, 0.24) | (-0.55, 0.58) | (-0.71, 0.65) | (-0.99, 1.04) | (-0.30, 1.01) | (-0.65, 0.64) | (-0.82, 0.50) | (-1.17, 0.83) | (-1.15, 1.59) | (-0.75, 1.72) |  | (-1.45, 0.54) | (-0.76, | (-0.66, |  |  |
|  |  |  |  |  |  |  |  |  |  |  |  | 0.48) | 0.58) |  |  |
| **-1.11** | -0.60 | -0.65 | -0.59 | -0.26 | -0.62 | -0.77 | -0.45 | -0.39 | -0.13 | -0.62 | ACEI/CCB | 0.00 | 0.27 | NA | NA |
| **(-2.03, -0.18)** | (-1.42, 0.22) | (-1.53, 0.24) | (-1.75, 0.56) | (-1.13, 0.60) | (-1.48, 0.23) | (-1.64, 0.09) | (-1.60, 0.70) | (-1.88, 1.09) | (-1.50, 1.24) | (-1.32, 0.08) |  | (-0.62, | (-0.08, |  |  |
|  |  |  |  |  |  |  |  |  |  |  |  | 0.62) | 0.62) |  |  |
| **-0.93** | -0.42 | -0.47 | -0.42 | -0.09 | -0.45 | -0.60 | -0.27 | -0.22 | 0.05 | -0.44 | 0.18 | TD/ARB | 0.27 | NA | 0.35 |
| **(-1.80, -0.07)** | (-1.22, 0.37) | (-1.29, 0.35) | (-1.52, 0.68) | (-0.89, 0.72) | (-1.23, 0.34) | (-1.37, 0.17) | (-1.37, 0.83) | (-1.66, 1.23) | (-1.31, 1.40) | (-1.31, 0.43) | (-1.06, 0.71) |  | (-0.00, |  | (-0.01, 0.71) |
|  |  |  |  |  |  |  |  |  |  |  |  |  | 0.54) |  |  |
| **-1.05** | -0.54 | -0.59 | -0.54 | -0.21 | -0.57 | -0.72 | -0.39 | -0.34 | -0.07 | -0.56 | 0.06 | -0.12 | ARB/CCB | NA | NA |
| **(-2.02, -0.08)** | (-1.43, 0.35) | (-1.52, 0.34) | (-1.72, 0.65) | (-1.12, 0.71) | (-1.47, 0.33) | (-1.61, 0.18) | (-1.57, 0.79) | (-1.85, 1.18) | (-1.49, 1.34) | (-1.44, 0.33) | (-0.71, 0.83) | (-0.81, |  |  |  |
|  |  |  |  |  |  |  |  |  |  |  |  | 0.57) |  |  |  |
| **-1.11** | -0.60 | -0.65 | -0.60 | -0.26 | -0.63 | -0.78 | -0.45 | -0.39 | -0.13 | -0.62 | -0.00 | -0.18 | -0.06 | ACEI/AR | NA |
| **(-2.00, -0.22)** | (-1.43, 0.23) | (-1.49, 0.20) | (-1.73, 0.54) | (-1.07, 0.54) | (-0.14, 1.39) | (-1.61, 0.06) | (-1.55, 0.65) | (-1.05, 1.84) | (-1.51, 1.25) | (-1.61, 0.38) | (-1.14, 1.14) | (-1.27, | (-1.24, | B |  |
|  |  |  |  |  |  |  |  |  |  |  |  | 0.92) | 1.12) |  |  |
| -0.83  (-2.02, 0.37) | -0.32  (-1.46, 0.83) | -0.36  (-1.53, 0.80) | -0.31  (-1.69, 1.06) | 0.02  (-1.13, 1.17) | -0.34  (-1.48, 0.79) | -0.49  (-1.62, 0.64) | -0.17  (-1.54, 1.21) | -0.11  (-1.77, 1.55) | 0.15  (-1.43, 1.74) | -0.33  (-1.53, 0.86) | 0.28  (-0.92, 1.49) | -0.11  (-0.71,  0.93) | 0.11  (-0.87,  1.10) | 0.28  (-1.08,  1.65) | BB/TD |

AAD: antialdosterone diuretics; AARA: Alpha-adrenergic receptor antagonists; ACEI: Angiotensin-converting enzyme inhibitors; ARB: Angiotensin receptor blockers; BB: beta-blockers; CCB: Calcium channel blockers; DAV: direct-acting vasodilators; PB: placebo; RI: renin inhibitors; TD: thiazide diuretics.

**Table S9.** Pooled mean differences of different types of antihypertensive drugs on peripheral pulse wave velocity.

| PB | NA | NA | NA | NA | -0.36  (-1.03, 0.31) | NA |
| --- | --- | --- | --- | --- | --- | --- |
| -0.04  (-1.67, 1.59) | BB | NA | -0.39  (-1.07, 0.29) | -0.09  (-0.48, 0.30) | **-0.82**  **(-1.60, -0.05)** | NA |
| 0.06  (-1.99, 2.11) | 0.10  (-1.66, 1.86) | TD | NA | NA | -0.36  (-1.06,0.34) | NA |
| -0.15  (-1.61, 1.30) | -0.12  (-1.01, 0.78) | -0.21  (-1.81, 1.38) | ACEI | 0.09  (-0.43, 0.61) | 0.01  (-0.46, 0.48) | **-1.13**  **(-2.06, 0.21)** |
| -0.43  (-1.87, 1.00) | -0.39  (-1.26, 0.48) | -0.49  (-2.06, 1.08) | -0.28  (-0.79, 0.23) | ARB | 0.34  (-0.02, 0.70) | **-0.97**  **(-1.89, -0.05)** |
| -0.07  (-1.45, 1.31) | -0.03  (-0.91, 0.85) | -0.13  (-1.65, 1.39) | 0.08  (-0.39, 0.56) | 0.36  (-0.03, 0.76) | CCB | NA |
| **-2.09**  **(-4.17, -0.01)** | **-2.05**  **(-3.79, -0.31)** | -2.15  (-4.33, 0.03) | **-1.93**  **(-3.47, -0.39)** | **-1.65**  **(-3.20, -0.11)** | **-2.02**  **(-3.58, -0.46)** | ACEI/ARB |

ACEI: Angiotensin-converting enzyme inhibitors; ARB: Angiotensin receptor blockers; BB: beta-blockers; CCB: Calcium channel blockers; PB: placebo; TD: thiazide diuretics

**Table S10.** Pooled mean differences of different types of antihypertensive drugs on pulse wave velocity in patients with exclusively hypertension.

| PB | -0.48  (-0.98, 0.02) | 0.07  (-0.38,  0.51) | **-0.39**  **(-0.76, -**  **0.02)** | NA | 0.01  (-0.47,  0.49) | -0.36  (-0.72, 0.00) | NA | NA | NA | NA | NA | NA | NA | NA | NA |
| --- | --- | --- | --- | --- | --- | --- | --- | --- | --- | --- | --- | --- | --- | --- | --- |
| -0.23  (-0.62,0.17) | BB | 0.09  (-0.30,  0.47) | NA | **-0.24**  **(-0.47, -**  **0.01)** | 0.09  (-0.10, 0.29) | -0.05  (-0.51, 0.41) | -0.46  -0.93, 0.02) | NA | -0.34  (-0.71,  0.03) | 0.02  (-0.15, 0.20) | NA | NA | NA | NA | NA |
| -0.20  (-0.61,0.20) | 0.03  (-0.27,0.33) | TD | NA | -0.30  (-0.65,  0.05) | -0.04  (-0.35, 0.28) | -0.17  (-0.41, 0.07) | NA | NA | NA | NA | NA | NA | NA | NA | NA |
| -0.41  (-0.97, 0.14) | -0.19  (-0.78,0.41) | -0.21  (- 0.81,0.39) | AAD | NA | NA | 0.20  (-0.14, 0.54) | NA | NA | NA | NA | NA | NA | NA | NA | NA |
| **-0.43**  **(-0.83,-0.02)** | -0.20  (-0.45,0.06) | -0.22  (- 0.51,0.07) | -0.01  (-0.60,0.58) | ACEI | -0.10  (-0.35,  0.15) | 0.20  (-0.04, 0.44) | 0.03  (-0.39, 0.45) | 0.15  (-0.35, 0.66) | NA | NA | NA | NA | NA | **-1.76**  **(-3.04, -0.48)** | NA |
| -0.30  (-0.68,0.09) | -0.07  (-0.30,0.17) | -0.09  (- 0.37,0.19) | 0.12  (-0.46,0.70) | 0.13 (-  0.10,0.36) | ARB | 0.15  (-0.07, 0.36) | NA | NA | NA | NA | NA | -0.34  (-1.09, 0.41) | NA | -1.06  (-2.49, 0.36) | NA |
| -0.13  (-0.50,0.23) | 0.09  (-0.16,0.35) | 0.07 (-  0.21,0.34) | 0.28  (-0.27,0.83) | **0.29**  **(0.05,0.53)** | 0.16  (-0.05,0.37) | CCB | NA | NA | NA | NA | NA | NA | NA | NA | NA |
| -0.34  (-1.06,0.38) | -0.11  (-0.75,0.52) | -0.14  (- 0.81,0.53) | 0.07  (-0.77,0.91) | 0.08 (-  0.53,0.70) | -0.05  (-0.69,0.60) | -0.21  (-0.86,0.44) | RI | NA | NA | NA | NA | NA | NA | NA | NA |
| -0.03  (-0.89,0.83) | 0.20  (-0.61,1.01) | 0.18 (-  0.64,1.00) | 0.39  (-0.58,1.36) | 0.40  (-0.37,1.17) | 0.27  (-0.53,1.07) | 0.11  (-0.70,0.91) | 0.32  (-0.67,1.30) | AARA | NA | NA | NA | NA | NA | NA | NA |
| -0.70  (-1.54,0.15) | -0.47  (-1.22,0.28) | -0.50  (- 1.30,0.31) | -0.28  (-1.24,0.67) | -0.27  (-1.06,0.52) | -0.40  (-1.19,0.38) | -0.56  (-1.36,0.23) | -0.36  (1.34,0.63) | -0.67  (-1.77,0.43) | DAV | NA | NA | NA | NA | NA | NA |
| -0.27  (-0.81,0.26) | -0.04  (-0.41,0.32) | -0.07  (- 0.54,0.40) | 0.14  (-0.55,0.84) | 0.15 (-  0.29,0.60) | 0.02  (-0.41,0.46) | -0.14  (-0.58,0.31) | 0.07  (-0.66,0.80) | -0.25  (-1.13,0.64) | 0.43 (-  0.41,1.26) | TD/ACEI | 0.30  (-0.44, 1.05) | 0.14  (-0.48, 0.76) | -0.04  (-0.66, 0.58) | NA | NA |
| -0.56  (-1.47,0.35) | -0.33  (-1.15,0.50) | -0.35  (- 1.22,0.51) | -0.14  (-1.15,0.86) | -0.13  (- 0.98,0.72) | -0.26  (-1.11,0.58) | -0.42  (-1.28,0.43) | -0.22  (-1.25,0.82) | -0.53  (-1.68,0.62) | 0.14 (-  0.97,1.26) | -0.29  (-1.06,0.49) | ACEI/CCB | 0.00  (-0.62, 0.62) | 0.09  (-0.53, 0.71) | NA | NA |
| -0.33  (-1.35,0.70) | -0.10  (-1.07,0.87) | -0.13  (- 1.11,0.86) | 0.09  (-1.03,1.20) | 0.10 (-  0.88,1.07) | -0.03  (-0.99,0.92) | -0.19  (-1.17,0.78) | 0.01  (-1.13,1.16) | -0.30  (-1.54,0.94) | 0.37 (-  0.85,1.59) | -0.06  (-1.03,0.92) | 0.23  (-0.74,1.20) | TD/ARB | -0.27  (-0.54, 0.00) | NA | 0.49  (-0.34,  1.31) |
| -0.37  (-1.41,0.68) | -0.14  (-1.12,0.85) | -0.16  (- 1.17,0.85) | 0.05  (-1.08,1.18) | 0.06  (-0.94,1.06) | -0.07  (-1.05,0.91) | -0.23  (-1.23,0.77) | -0.02  (-1.19,1.14) | -0.34  (-1.60,0.92) | 0.33 (-  0.91,1.57) | -0.09  (-1.08,0.90) | 0.19  (-0.74,1.13) | -0.04  (-0.53,0.45) | ARB/CCB | NA | NA |
| **-0.83**  **(-1.49,-0.17)** | **-0.60**  **(-1.19,-0.02)** | **-0.63**  **(-1.23,-**  **0.03)** | -0.42  (-1.21,0.37) | -0.41  (-0.97,0.16) | **-0.54**  **(-1.07,-**  **0.00)** | **-0.70**  **(-1.28,-0.12)** | -0.49  (-1.32,0.34) | -0.81  (-1.76,-0.15) | -0.13  (- 1.08,0.82) | -0.56  (-1.24,0.12) | -0.28  (-1.26,0.71) | -0.52  (-1.59,0.58) | -0.47  (-1.58,0.65) | ACEI/ARB | NA |
| -0.12  (-1.28,1.03) | 0.10  (-1.00,1.21) | 0.08 (-  1.05,1.20) | 0.29  (-0.95,1.53) | 0.30 (-  0.81,1.42) | 0.17  (-0.93,1.27) | 0.01  (-1.10,1.12) | 0.22  (-1.04,1.48) | -0.10  (-1.45,1.26) | 0.57 (-  0.76,1.91) | 0.15  (-0.97,1.27) | 0.43  (-0.68,1.54) | 0.20  (-0.33,0.74) | 0.24  (-0.48,0.97) | 0.71  (-0.50,1.92) | BB/TD |

AAD: antialdosterone diuretics; AARA: Alpha-adrenergic receptor antagonists; ACEI: Angiotensin-converting enzyme inhibitors; ARB: Angiotensin receptor blockers; BB: beta-blockers; CCB: Calcium channel blockers; DAV: direct-acting vasodilators; PB: placebo; RI: renin inhibitors; TD: thiazide diuretics.

**Table S11.** Pooled mean differences of different types of antihypertensive drugs on augmentation index in patients with exclusively hypertension.

| PB | **0.73**  **(0.22, 1.24)** | -0.24  (-0.60, 0.12) | -0.24  (-0.62, 0.14) | -1.00  (-2.61, 0.60) | -0.25  (-0.73, 0.23) | NA | -0.32  (-0.83, 0.19) | NA | NA | NA | NA | NA |
| --- | --- | --- | --- | --- | --- | --- | --- | --- | --- | --- | --- | --- |
| 0.15  (-0.46, 0.76) | BB | -0.47  (-1.20, 0.26) | -0.44  (-1.21, 0.33) | -0.35  (-0.73, 0.04) | **-1.20**  **(-1.75, -0.66)** | 0.41  (-0.16, 0.98) | -0.22  (-0.73, 0.29) | **-0.39**  **(-0.58, -0.19)** | NA | NA | NA | NA |
| -0.37  (-0.99, 0.26) | -0.52  (-1.17, 0.14) | TD | -0.37  (-0.72, 0.02) | -0.83  (-2.51, 0.86) | 0.09  (-0.46, 0.65) | NA | -0.07  (-0.57, 0.44) | **-1.02**  **(-1.58, -0.46)** | NA | NA | NA | NA |
| **-0.64**  **(-1.24, -0.04)** | **-0.79**  **(-1.27,- 0.32)** | -0.27  (-0.89, 0.35) | ACEI | 0.64  (-0.13, 1.40) | 0.02  (-0.49, 0.53) | -0.14  (-0.49, 0.21) | 0.21  (-0.30, 0.71) | NA | NA | NA | NA | NA |
| -0.43  (-1.00, 0.14) | **-0.58**  **(-1.08, -0.09)** | -0.07  (-0.65, 0.52) | 0.21  (-0.25, 0.66) | ARB | -0.02  (-0.26, 0.22) | NA | NA | NA | NA | -0.45  (-1.21, 0.31) | NA | NA |
| -0.40  (-1.08, 0.28) | -0.55  (-1.20, 0.10) | -0.03  (-0.71, 0.65) | 0.24  (-0.39, 0.87) | 0.03  (-0.56, 0.62) | CCB | NA | 0.13  (-0.38, 0.64) | **-1.33**  **(-1.91, -0.75)** | NA | NA | NA | NA |
| -0.52  (-1.40, 0.37) | -0.67  (-1.40, 0.06) | -0.15  (-1.06, 0.76) | 0.12  (-0.61, 0.85) | -0.09  (-0.89, 0.72) | -0.12  (-1.03, 0.57) | RI | NA | NA | NA | NA | NA | NA |
| -0.54  (-2.03, 0.96) | -0.69  (-2.14, 0.76) | -0.17  (-1.68, 1.33) | 0.10  (-1.27, 1.47) | -0.11  (-1.55, 1.34) | -0.14  (-1.65, 1.37) | -0.02  (-1.57, 1.53) | AARA | NA | NA | NA | NA | NA |
| -0.59  (-1.40, 0.22) | **-0.74**  **(-1.37, -0.12)** | -0.23  (-1.04, 0.59) | 0.05  (-0.68, 0.78) | -0.16  (-0.78, 0.42) | -0.19  (-1.02, 0.63) | -0.07  (-1.01, 0.86) | -0.05  (-1.60, 1.50) | TD/ACEI | -0.14  (-0.76, 0.48) | 0.02  (-0.60, 0.64) | 0.01  (-0.61, 0.63) | NA |
| -0.93  (-2.18, 0.32) | -1.08  (-2.23, 0.07) | -0.56  (-1.82, 0.69) | -0.29  (-1.49, 0.91) | -0.50  (-0.88, 0.56) | -0.53  (-1.79, 0.73) | -0.41  (-1.75, 0.93) | -0.39  (-2.21, 1.43) | -0.34  (-1.36, 0.69) | ACEI/CCB | 0.14  (-0.48, 0.76) | 0.15  (-0.47, 0.77) | NA |
| -0.54  (-1.62, 0.54) | -0.70  (-1.67, 0.28) | -0.18  (-1.26, 0.91) | 0.10  (-0.93, 1.12) | -0.11  (-1.10, 0.88) | -0.14  (-1.24, 0.95) | 0.02  (-1.17, 1.21) | 0.00  (-1.71, 1.71) | 0.05  (-0.82, 0.92) | 0.39  (-0.59, 1.36) | TD/ARB | -0.16  (-0.34, 0.03) | **0.90**  **(0.18, 1.62)** |
| -0.75  (-1.92, 0.43) | -0.90  (-1.97, 0.17) | -0.38  (-1.56, 0.79) | -0.11  (-1.23, 1.01) | -0.32  (-1.42, 0.78) | -0.35  (-1.54, 0.84) | -0.23  (-1.50, 1.04) | -0.21  (-1.98, 1.56) | -0.16  (-1.10, 0.79) | 0.18  (-0.82, 1.18) | -0.21  (-0.99, 0.58) | ARB/CCB | NA |
| -0.03  (-1.50, 1.44) | -0.18  (-1.58, 1.21) | 0.34  (-1.14, 1.81) | 0.61  (-0.82, 2.04) | 0.40  (-1.00, 1.81) | 0.37  (-1.11, 1.85) | 0.49  (-1.06, 2.04) | 0.51  (-1.47, 2.49) | 0.56  (-0.76, 1.88) | 0.90  (-0.50, 2.29) | 0.51  (-0.48, 1.51) | 0.72  (-0.55, 1.99) | BB/TD |

AARA: Alpha-adrenergic receptor antagonists; ACEI: Angiotensin-converting enzyme inhibitors; ARB: Angiotensin receptor blockers; BB: beta-blockers; CCB: Calcium channel blockers; PB: placebo; RI: renin inhibitors; TD: thiazide diuretics.

**Table S12.** Meta-regression according to mean age, percentage of female and length of treatment for different types of antihypertensive drugs on pulse wave velocity.

|  | **Coefficient** | **95%CIs** | **P value** |
| --- | --- | --- | --- |
| **ACEI-ARB** | | | |
| Mean age | 0.024 | -0.041, 0.089 | 0.436 |
| % Female | -0.013 | -0.049, 0.023 | 0.423 |
| Length of treatment | -0.005 | -0.060, 0.060 | 0.830 |
| Systolic blood pressure | 0.053 | -0.006, 0.112 | 0.069 |
| Diastolic blood pressure | 0.146 | -0.085, 0.377 | 0.174 |
| **ACEI-CCB** | | | |
| Mean age | -0.020 | -0.059, 0.018 | 0.253 |
| % Female | 0.012 | -0.004, 0.028 | 0.112 |
| Length of treatment | 0.006 | -0.018, 0.030 | 0.562 |
| Systolic blood pressure | -0.056 | -0.122, 0.012 | 0.089 |
| Diastolic blood pressure | **-0.032** | **-0.064, -0.000** | **0.049** |
| **ACEI-PB** | | | |
| Mean age | -0.059 | -0.289, 0.171 | 0.387 |
| % Female | -0.117 | -1.363, 1.130 | 0.445 |
| Length of treatment | 0.042 | -0.153, 0.237 | 0.455 |
| Systolic blood pressure | -0.099 | -0.542, 0.343 | 0.436 |
| Diastolic blood pressure | -0.299 | -1.378, 0.779 | 0.355 |
| **ARB-ACEI/ARB** | | | |
| Mean age | -0.125 | -0.456, 0.207 | 0.131 |
| % Female | - | - | - |
| Length of treatment | -0.053 | -0.194, 0.089 | 0.132 |
| Systolic blood pressure | 0.502 | -3.571, 4.576 | 0.362 |
| Diastolic blood pressure | 1.024 | -2.302, 4.349 | 0.159 |
| **ARB-CCB** | | | |
| Mean age | **-0.063** | **-0.097, -0.030** | **0.001** |
| % Female | 0.009 | -0.004, 0.021 | 0.149 |
| Length of treatment | 0.008 | -0.006, 0.023 | 0.233 |
| Systolic blood pressure | -0.072 | -0.195, 0.050 | 0.219 |
| Diastolic blood pressure | **-0.070** | **-0.112, -0.030** | **0.003** |
| **BB-ACEI** | | | |
| Mean age | -0.023 | -0.062, 0.016 | 0.204 |
| % Female | -0.024 | -0.056, 0.008 | 0.118 |
| Length of treatment | 0.003 | -0.041, 0.047 | 0.878 |
| Systolic blood pressure | -0.011 | -0.079, 0.057 | 0.709 |
| Diastolic blood pressure | 0.015 | -0.177, 0.207 | 0.856 |
| **BB-ARB** | | | |
| Mean age | 0.009 | -0.061, 0.080 | 0.728 |
| % Female | -0.005 | -0.046, 0.035 | 0.728 |
| Length of treatment | 0.008 | -0.046, 0.062 | 0.706 |
| Systolic blood pressure | 0.019 | -0.029, 0.068 | 0.337 |
| Diastolic blood pressure | 0.041 | -0.162, 0.243 | 0.606 |
| **BB-CCB** | | | |
| Mean age | -0.035 | -0.191, 0.120 | 0.432 |
| % Female | -0.030 | -0.089, 0.029 | 0.161 |
| Length of treatment | -0.018 | -0.207, 0.172 | 0.729 |
| Systolic blood pressure | 0.077 | -0.087, 0.242 | 0.179 |
| Diastolic blood pressure | 0.102 | -0.197, 0.402 | 0.279 |
| **BB-TD** | | | |
| Mean age | -0.031 | -0.383, 0.320 | 0.460 |
| % Female | 0.128 | -6.440, 6.695 | 0.846 |
| Length of treatment | 0.036 | -0.363, 0.435 | 0.459 |
| Systolic blood pressure | 0.076 | -0.770, 0.922 | 0.459 |

| Diastolic blood pressure | 0.095 | -0.993, 1.183 | 0.467 |
| --- | --- | --- | --- |
| **TD-ACEI** | | | |
| Mean age | - | - | - |
| % Female | 0.016 | -0.016, 0.048 | 0.161 |
| Length of treatment | 0.024 | -0.013, 0.062 | 0.132 |
| Systolic blood pressure | -0.002 | -0.134, 0.130 | 0.963 |
| Diastolic blood pressure | -0.007 | -0.293, 0.278 | 0.941 |
| **TD-ARB** | | | |
| Mean age | 0.013 | -0.747, 0.773 | 0.866 |
| % Female | -0.024 | -1.518, 1.469 | 0.869 |
| Length of treatment | -0.006 | -0.351, 0.339 | 0.865 |
| Systolic blood pressure | 0.009 | -0.549, 0.569 | 0.865 |
| Diastolic blood pressure | 0.100 | -5.810, 6.010 | 0.865 |
| **TD-CCB** | | | |
| Mean age | 0.002 | -0.082, 0.086 | 0.953 |
| % Female | -0.021 | -0.076, 0.033 | 0.301 |
| Length of treatment | -0.028 | -0.081, 0.026 | 0.200 |
| Systolic blood pressure | 0.031 | -0.063, 0.124 | 0.371 |
| Diastolic blood pressure | 0.027 | -0.156, 0.209 | 0.674 |
| **TD/ACEI-ACEI/CCB** | | | |
| Mean age | -0.109 | -0.964, 0.746 | 0.353 |
| % Female | -0.038 | -0.331, 0.255 | 0.348 |
| Length of treatment | -0.050 | -0.430, 0.330 | 0.343 |
| Systolic blood pressure | -0.098 | -0.936, 0.739 | 0.377 |
| Diastolic blood pressure | -0.077 | -0.666, 0.511 | 0.344 |

AARA: Alpha-adrenergic receptor antagonists; ACEI: Angiotensin-converting enzyme inhibitors; ARB: Angiotensin receptor blockers; BB: beta-blockers; CCB: Calcium channel blockers; DAV: direct-acting vasodilators; PB: placebo; RI: renin inhibitors; TD: thiazide diuretics.

**Table S13.** Meta-regression according to mean age, percentage of female and length of treatment for different types of antihypertensive drugs on augmentation index.

|  | **Coefficient** | **95%CIs** | **P value** |
| --- | --- | --- | --- |
| **ACEI-ARB** | | | |
| Mean age | -0.483 | -2.661, 1.696 | 0.532 |
| % Female | -0.161 | -0.678, 0.355 | 0.393 |
| Length of treatment | - | - | - |
| Systolic blood pressure | **0.090** | **0.015, 0.164** | **0.031** |
| Diastolic blood pressure | **0.168** | **0.056, 0.280** | **0.017** |
| **ACEI-CCB** | | | |
| Mean age | -0.027 | -0.577, 0.522 | 0.640 |
| % Female | -0.013 | -1.038, 1.012 | 0.901 |
| Length of treatment | -0.095 | -0.963, 0.774 | 0.397 |
| Systolic blood pressure | -0.097 | -2.091, 1.898 | 0.649 |
| Diastolic blood pressure | -0.238 | -2.572, 2.096 | 0.418 |
| **ACEI-PB** | | | |
| Mean age | -0.093 | -0.356, 0.169 | 0.340 |
| % Female | -0.004 | -0.241, 0.234 | 0.954 |
| Length of treatment | 0.015 | -0.149, 0.180 | 0.784 |
| Systolic blood pressure | -0.020 | -0.602, 0.562 | 0.919 |
| Diastolic blood pressure | 0.118 | -0.852, 1.089 | 0.724 |
| **BB-ACEI** | | | |
| Mean age | 0.054 | -0.041, 0.149 | 0.192 |
| % Female | 0.045 | -0.064, 0.155 | 0.319 |
| Length of treatment | -0.049 | -0.729, 0.631 | 0.850 |
| Systolic blood pressure | 0.026 | -0.129, 0.180 | 0.670 |
| Diastolic blood pressure | 0.064 | -0.326, 0.454 | 0.672 |
| **BB-TD/ACEI** | | | |
| Mean age | - | - | - |
| % Female | - | - | - |
| Length of treatment | - | - | - |
| Systolic blood pressure | -0.030 | -0.330, 0.270 | 0.708 |
| Diastolic blood pressure | 0.151 | -11.544, 11.846 | 0.897 |
| **TD-CCB** | | | |
| Mean age | 0.045 | -1.061, 1.151 | 0.697 |
| % Female | -0.022 | -1.957, 1.914 | 0.909 |
| Length of treatment | 0.151 | -2.373, 2.676 | 0.586 |
| Systolic blood pressure | - | - | - |
| Diastolic blood pressure | - | - | - |

ACEI: Angiotensin-converting enzyme inhibitors; ARB: Angiotensin receptor blockers; BB: beta-blockers; CCB: Calcium channel blockers; PB: placebo; TD: thiazide diuretics.

**Table S14.** Heterogeneity statistics for each comparison for pulse wave velocity.

|  | **Q (df)** | **I2** | τ2 | **p** |
| --- | --- | --- | --- | --- |
| **TD-PB** | 0.00 (0) | - | 0.00 | - |
| **AAD-PB** | - | - | - | - |
| **BB-PB** | 0.04 (1) | 0.00% | 0.00 | 0.844 |
| **ACEI-PB** | 17.79 (3) | 83.10% | 0.81 | 0.000 |
| **ARB-PB** | 0.00 (0) | - | 0.00 | - |
| **CCB-PB** | 0.77 (1) | 0.00% | 0.00 | 0.381 |
| **BB-TD** | 1.29 (2) | 0.00% | 0.00 | 0.523 |
| **BB-ACEI** | 5.29 (8) | 0.00% | 0.00 | 0.727 |
| **BB-ARB** | 3.66 (5) | 0.00% | 0.00 | 0.599 |
| **BB-CCB** | 5.24 (3) | 42.70% | 0.09 | 0.155 |
| **BB-RI** | 0.17 (1) | 0.00% | 0.00 | 0.680 |
| **BB-DAV** | 0.00 (0) | - | 0.00 | - |
| **TD-ACEI** | 4.96 (4) | 19.40% | 0.03 | 0.291 |
| **TD-ARB** | 0.05 (2) | 0.00% | 0.00 | 0.977 |
| **TD-CCB** | 3.25 (4) | 0.00% | 0.00 | 0.516 |
| **AAD-CCB** | - | - | - | - |
| **ACEI-ARB** | 59.38 (11) | 81.50% | 0.41 | 0.000 |
| **ACEI-CCB** | 12.01 (8) | 33.40% | 0.05 | 0.151 |
| **ACEI-AARA** | 0.00 (0) | - | 0.00 | - |
| **ACEI-RI** | 0.03 (1) | 0.00% | 0.00 | 0.870 |
| **ARB-CCB** | 46.06 (13) | 71.80% | 0.14 | 0.000 |
| **ARB-RI** | 0.00 (0) | - | 0.00 | - |
| **BB-TD/ACEI** | 2.68 (2) | 25.40% | 0.01 | 0.262 |
| **BB/TD-TD/ARB** | 1.76 (1) | 43.10% | 0.18 | 0.185 |
| **TD/ARB-CCB** | 0.00 (0) | - | 0.00 | - |
| **TD/ACEI-TD/ARB** | 0.00 (0) | - | 0.00 | - |
| **TD/ACEI-ARB/CCB** | 0.00 (0) | - | 0.00 | - |
| **TD/ACEI-ACEI/CCB** | 2.79 (2) | 28.30% | 0.04 | 0.248 |
| **TD/ARB-ACEI/CCB** | 0.00 (0) | - | 0.00 | - |
| **TD/ARB-ARB/CCB** | - | - | - | - |
| **ACEI-ACEI/ARB** | 3.33 (1) | 70.00% | 0.60 | 0.068 |
| **ACEI/CCB-ARB/CCB** | 0.46 (1) | 0.00% | 0.00 | 0.496 |
| **ARB-TD/ARB** | 0.00 (0) | - | 0.00 | - |
| **ARB-ACEI/ARB** | 23.03 (2) | 91.30% | 1.41 | 0.000 |

AAD: antialdosterone diuretics; AARA: Alpha-adrenergic receptor antagonists; ACEI: Angiotensin- converting enzyme inhibitors; ARB: Angiotensin receptor blockers; BB: beta-blockers; CCB: Calcium channel blockers; DAV: direct-acting vasodilators; PB: placebo; RI: renin inhibitors; TD: thiazide diuretics.

**Table S15.** Heterogeneity statistics for each comparison for augmentation index.

|  | **Q (df)** | **I2** | τ2 | **p** |
| --- | --- | --- | --- | --- |
| **BB-PB** | 0.51 (1) | 0.00% | 0.00 | 0.475 |
| **TD-PB** | 0.00 (1) | 0.00% | 0.00 | 1.000 |
| **ACEI-PB** | 48.67 (4) | 91.80% | 1.40 | 0.000 |
| **ARB-PB** | 13.36 (1) | 92.50% | 1.24 | 0.000 |
| **CCB-PB** | 0.00 (0) | - | 0.00 | - |
| **BB-TD** | 0.00 (0) | - | 0.00 | - |
| **BB-ACEI** | 25.53 (5) | 80.40% | 0.41 | 0.000 |
| **BB-ARB** | 1.57 (1) | 36.40% | 0.03 | 0.210 |
| **BB-CCB** | 0.66 (1) | 0.00% | 0.00 | 0.418 |
| **BB-RI** | 1.87 (1) | 46.50% | 0.12 | 0.172 |
| **TD-ACEI** | 0.15 (1) | 0.00% | 0.00 | 0.694 |
| **TD-ARB** | 15.61 (1) | 93.60% | 1.38 | 0.000 |
| **TD-CCB** | 5.67 (2) | 64.70% | 0.15 | 0.059 |
| **ACEI-ARB** | 24.25 (4) | 83.50% | 0.36 | 0.000 |
| **ACEI-CCB** | 1.96 (2) | 0.00% | 0.00 | 0.375 |
| **ACEI-AARA** | 0.00 (0) | - | 0.00 | - |
| **ACEI-RI** | 0.09 (1) | 0.00% | 0.00 | 0.763 |
| **ARB-CCB** | 0.00 (1) | 0.00% | 0.00 | 1.000 |
| **ARB-RI** | 0.00 (0) | - | 0.00 | - |
| **CCB-TD/ARB** | 0.00 (0) | - | 0.00 | - |
| **BB-TD/ACEI** | 5.69 (3) | 47.30% | 0.02 | 0.128 |
| **BB/TD-TD/ARB** | 2.20 (1) | 54.60% | 0.15 | 0.138 |
| **TD-TD/ACEI** | 0.00 (0) | - | 0.00 | - |
| **TD/ACEI-TD/ARB** | 0.00 (0) | - | 0.00 | - |
| **TD/ACEI-ARB/CCB** | 0.00 (0) | - | 0.00 | - |
| **TD/ACEI-ACEI/CCB** | 0.00 (0) | - | 0.00 | - |
| **TD/ARB-ACEI/CCB** | 0.00 (0) | - | 0.00 | - |
| **TD/ARB-ARB/CCB** | 0.23 (1) | 0.00% | 0.00 | 0.629 |
| **ACEI/CCB-ARB/CCB** | 1.45 (1) | 31.00% | 0.03 | 0.229 |
| **ARB-TD/ARB** | 0.00 (0) | - | 0.00 | - |
| **CCB-TD/ACEI** | 0.00 (0) | - | 0.00 | - |

AARA: Alpha-adrenergic receptor antagonists; ACEI: Angiotensin-converting enzyme inhibitors; ARB: Angiotensin receptor blockers; BB: beta-blockers; CCB: Calcium channel blockers; PB: placebo; RI: renin inhibitors; TD: thiazide diuretics.

**Figure. S1.** Risk of bias using RoB2 tool.


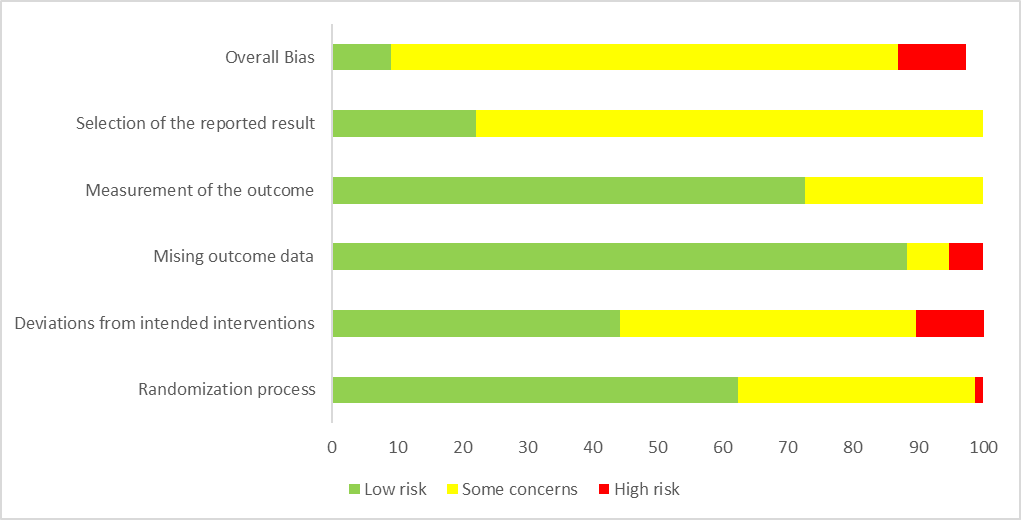


**Figure S2.** Network of available comparisons between different types of antihypertensive drugs on pulse wave velocity including only studies over 6 months duration.


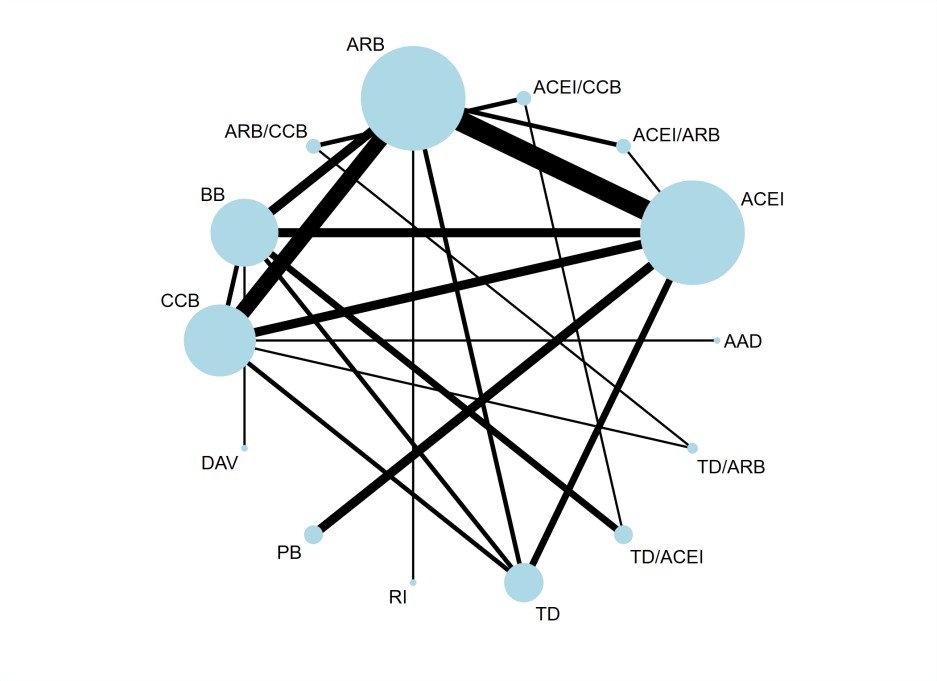


AAD: antialdosterone diuretics; ACEI: Angiotensin-converting enzyme inhibitors; ARB: Angiotensin receptor blockers; BB: beta-blockers; CCB: Calcium channel blockers; DAV: direct-acting vasodilators; PB: placebo; RI: renin inhibitors; TD: thiazide diuretics.

**Figure S3.** Network of available comparisons between different types of antihypertensive drugs on augmentation index including only studies longer than 6 months length.


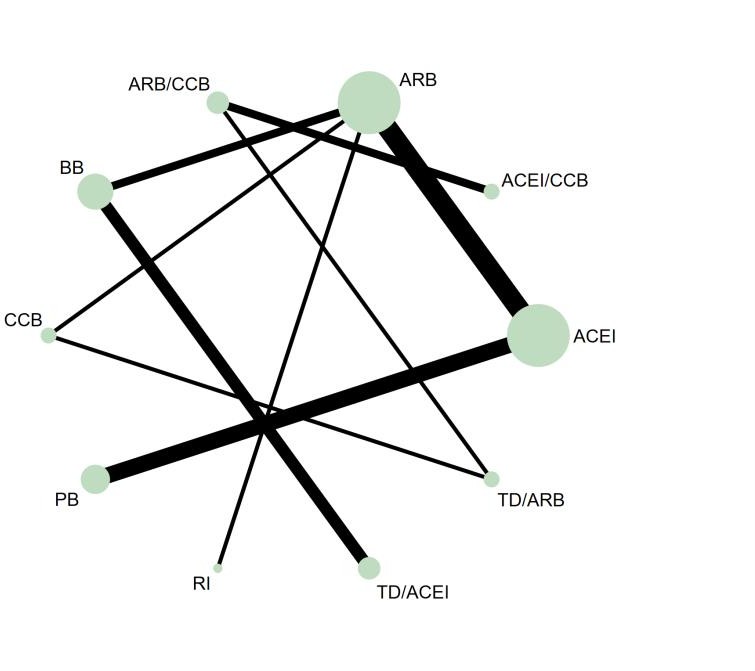


ACEI: Angiotensin-converting enzyme inhibitors; ARB: Angiotensin receptor blockers; BB: beta-blockers; CCB: Calcium channel blockers; DAV: direct-acting vasodilators; PB: placebo; RI: renin inhibitors; TD: thiazide diuretics.

**Figure S4.** Network of available comparisons between different types of antihypertensive

drugs on central pulse wave velocity.


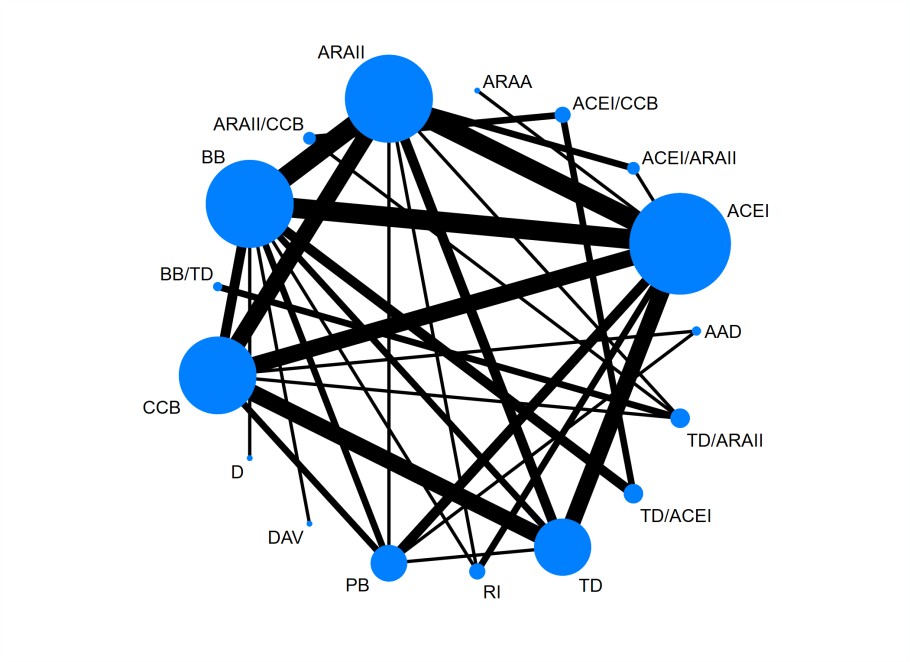


AAD: antialdosterone diuretics; AARA: Alpha-adrenergic receptor antagonists; ACEI: Angiotensin- converting enzyme inhibitors; ARB: Angiotensin receptor blockers; BB: beta-blockers; CCB: Calcium channel blockers; DAV: direct-acting vasodilators; PB: placebo; RI: renin inhibitors; TD: thiazide diuretics.

**Figure S5.** Network of available comparisons between different types of antihypertensive drugs on peripheral pulse wave velocity.


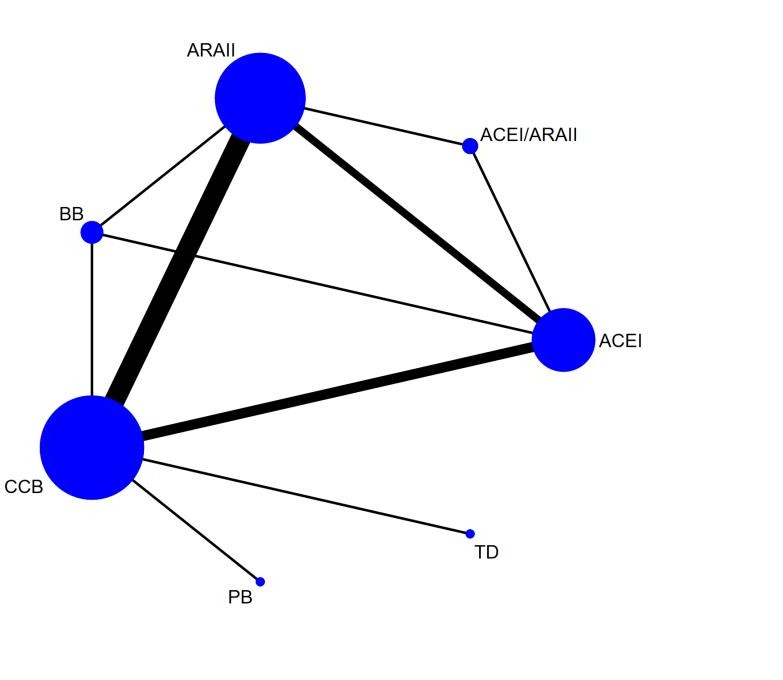


ACEI: Angiotensin-converting enzyme inhibitors; ARB: Angiotensin receptor blockers; BB: beta-blockers; CCB: Calcium channel blockers; PB: placebo; TD: thiazide diuretics.

**Figure S6.** Network of available comparisons between different types of antihypertensive drugs on pulse wave velocity in patients with exclusively hypertension.


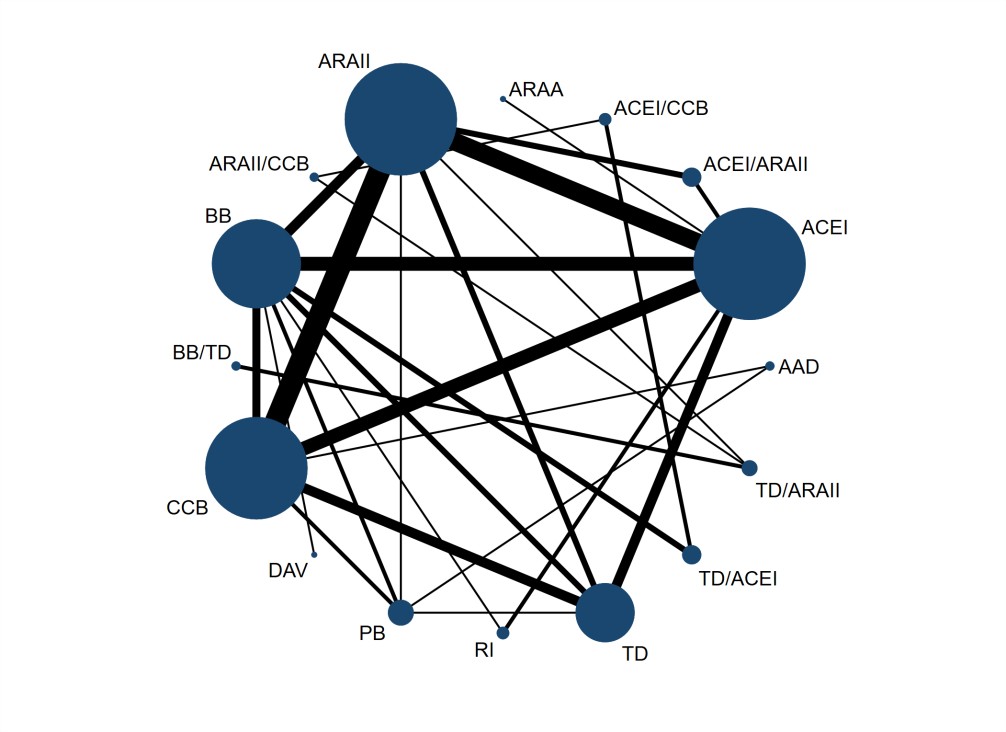


AAD: antialdosterone diuretics; AARA: Alpha-adrenergic receptor antagonists; ACEI: Angiotensin- converting enzyme inhibitors; ARB: Angiotensin receptor blockers; BB: beta-blockers; CCB: Calcium channel blockers; DAV: direct-acting vasodilators; PB: placebo; RI: renin inhibitors; TD: thiazide diuretics.

**Figure S7.** Network of available comparisons between different types of antihypertensive drugs on augmentation index in patients with exclusively hypertension.


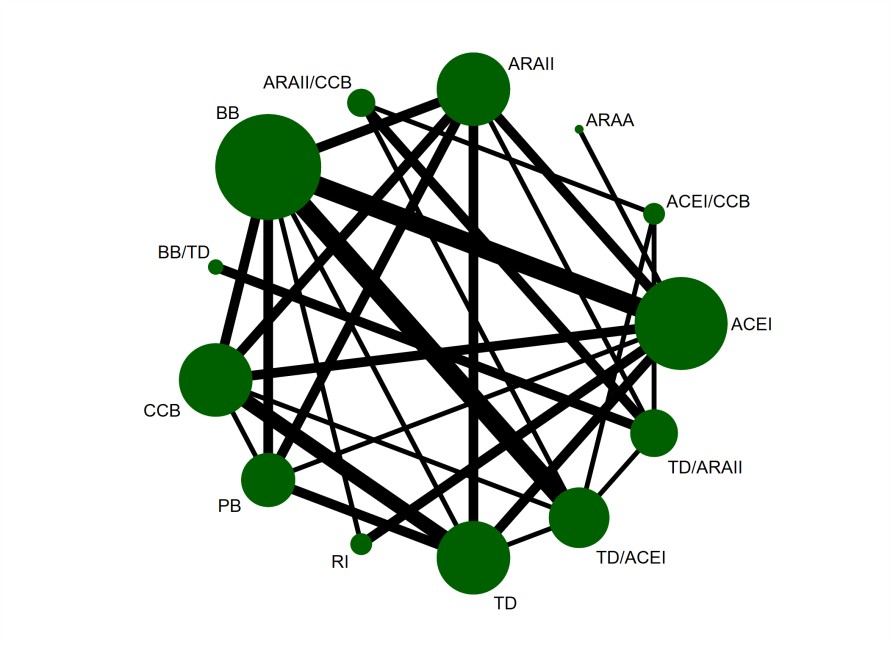


AARA: Alpha-adrenergic receptor antagonists; ACEI: Angiotensin-converting enzyme inhibitors; ARB: Angiotensin receptor blockers; BB: beta-blockers; CCB: Calcium channel blockers; PB: placebo; RI: renin inhibitors; TD: thiazide diuretics.

# Supplementary References

- Asmar RG, Benetos A, Chaouche-Teyara K, Raveau-Landon CM, Safar ME. Comparison of effects of felodipine versus hydrochlorothiazide on arterial diameter and pulse-wave velocity in essential hypertension. The American journal of cardiology. 1993 Oct 1;72(11):794-8.
- Merli I, Simon A, Pino MD, Brautigam M, Welzel D, Levenson J. Intrinsic effect of antihypertensive treatment with isradipine and metoprolol on large artery geometric and elastic properties. Clinical Pharmacology & Therapeutics. 1993 Jul;54(1):76-83.
- London GM, Pannier B, Guerin AP, Marchais SJ, Safar ME, Cuche JL. Cardiac hypertrophy, aortic compliance, peripheral resistance, and wave reflection in end- stage renal disease. Comparative effects of ACE inhibition and calcium channel blockade. Circulation. 1994 Dec;90(6):2786-96.
- Chen CH, Ting CT, Lin SJ, Hsu TL, Yin FC, Siu CO, Chou P, Wang SP, Chang MS. Different effects of fosinopril and atenolol on wave reflections in hypertensive patients. Hypertension. 1995 May;25(5):1034-41.
- Breithaupt-Grögler K, Leschinger M, Belz GG, Butzer R, Erb K, de Mey C, Sinn

W. Influence of antihypertensive therapy with cilazapril and hydrochlorothiazide on the stiffness of the aorta. Cardiovascular drugs and therapy. 1996 Mar;10:49- 57.

- Savolainen A, Keto P, Poutanen VP, Hekali P, Standertskjöld-Nordenstam CG, Rames A, Kupari M. Effects of angiotensin-converting enzyme inhibition versus β-adrenergic blockade on aortic stiffness in essential hypertension. Journal of cardiovascular pharmacology. 1996 Jan 1;27(1):99-104.
- Armentano RL, Graf S, Ramirez AJ, Espinosa JD, Brandani L, Baglivo H, Sanchez R. Mechanical vs intrinsic components in the improvement of brachial arterial compliance. Medicina (Buenos Aires). 2001;61(5/1):535-40.
- Asmar RG, London GM, O’Rourke ME, Safar ME, REASON Project Coordinators and Investigators. Improvement in blood pressure, arterial stiffness and wave reflections with a very-low-dose perindopril/indapamide combination in hypertensive patient: a comparison with atenolol. Hypertension. 2001 Oct 1;38(4):922-6.
- Asmar RG, London GM, O'Rourke ME, Mallion JM, Romero R, Rahn KH, Trimarco B, Fitzgerald D, Hedner T, Duprez D, De Leeuw PW. Amelioration of arterial properties with a perindopril-indapamide very-low-dose combination. Journal of hypertension. Supplement: official journal of the International Society of Hypertension. 2001 Nov 1;19(4):S15-20.
- Dart AM, Reid CM, McGrath B. Effects of ACE inhibitor therapy on derived central arterial waveforms in hypertension. American journal of hypertension. 2001 Aug 1;14(8):804-10.
- Klingbeil AU, John S, Schneider MP, Jacobi J, Weidinger G, Schmieder RE. AT1-receptor blockade improves augmentation index: a double-blind,

randomized, controlled study. Journal of hypertension. 2002 Dec 1;20(12):2423- 8.

- Komai N, Ohishi M, Morishita R, Moriguchi A, Kaibe M, Matsumoto K, Rakugi H, Higaki J, Ogihara T. Serum hepatocyte growth factor concentration is correlated with the forearm vasodilator response in hypertensive patients. American journal of hypertension. 2002 Jun 1;15(6):499-506.
- Mahmud A, Feely J. Reduction in arterial stiffness with angiotensin II antagonist is comparable with and additive to ACE inhibition. American journal of hypertension. 2002 Apr 1;15(4):321-5.
- Rajzer M, Klocek M, Kawecka-Jaszcz K. Effect of amlodipine, quinapril, andlosartan on pulse wave velocity andplasma collagen markers in patients withmild-to-moderate arterial hypertension. American journal of hypertension. 2003 Jun 1;16(6):439-44.
- Takami T, Shigemasa M. Efficacy of various antihypertensive agents as evaluated by indices of vascular stiffness in elderly hypertensive patients. Hypertension Research. 2003;26(8):609-14.
- White WB, Duprez D, St Hillaire R, Krause S, Roniker B, Kuse-Hamilton J, Weber MA. Effects of the selective aldosterone blocker eplerenone versus the calcium antagonist amlodipine in systolic hypertension. Hypertension. 2003 May;41(5):1021-6.
- de Luca N, Asmar RG, London GM, O'Rourke MF, Safar ME, REASON Project Investigators. Selective reduction of cardiac mass and central blood pressure on low-dose combination perindopril/indapamide in hypertensive subjects. Journal of hypertension. 2004 Aug 1;22(8):1623-30.
- London GM, Asmar RG, O'Rourke MF, Safar ME, REASON Project Investigators. Mechanism (s) of selective systolic blood pressure reduction after a low-dose combination of perindopril/indapamide in hypertensive subjects: comparison with atenolol. Journal of the American College of Cardiology. 2004 Jan 7;43(1):92-9.
- Munakata M, Nagasaki A, Nunokawa T, Sakuma T, Kato H, Yoshinaga K, Toyota

T. Effects of valsartan and nifedipine coat-core on systemic arterial stiffness in hypertensive patients. American journal of hypertension. 2004 Nov 1;17(11):1050-5.

- Neal DA, Brown MJ, Wilkinson IB, Byrne CD, Alexander GJ. Hemodynamic effects of amlodipine, bisoprolol, and lisinopril in hypertensive patients after liver transplantation1. Transplantation. 2004 Mar 15;77(5):748-50.
- Anan F, Takahashi N, Ooie T, Yufu K, Hara M, Nakagawa M, Yonemochi H, Saikawa T, Yoshimatsu H. Effects of valsartan and perindopril combination therapy on left ventricular hypertrophy and aortic arterial stiffness in patients with essential hypertension. European journal of clinical pharmacology. 2005 Jul;61:353-9.
- Ichihara A, Kaneshiro Y, Takemitsu T, Sakoda M. Effects of amlodipine and valsartan on vascular damage and ambulatory blood pressure in untreated hypertensive patients. Journal of human hypertension. 2006 Oct;20(10):787-94.
- Kaiser T, Heise T, Nosek L, Eckers U, Sawicki PT. Influence of nebivolol and enalapril on metabolic parameters and arterial stiffness in hypertensive type 2 diabetic patients. Journal of hypertension. 2006 Jul 1;24(7):1397-403.
- Morimoto S, Yano Y, Maki K, Sawada K. Renal and vascular protective effects of telmisartan in patients with essential hypertension. Hypertension research. 2006 Aug;29(8):567-72.
- Yu WC, Lin YP, Lin IF, Chuang SY, Chen CH. Effect of ramipril on left ventricular mass in normotensive hemodialysis patients. American journal of kidney diseases. 2006 Mar 1;47(3):478-84.
- Jiang XJ, O'Rourke MF, Zhang YQ, He XY, Liu LS. Superior effect of an angiotensin-converting enzyme inhibitor over a diuretic for reducing aortic systolic pressure. Journal of hypertension. 2007 May 1;25(5):1095-9.
- Mitchell GF, Dunlap ME, Warnica W, Ducharme A, Arnold JM, Tardif JC, Solomon SD, Domanski MJ, Jablonski KA, Rice MM, Pfeffer MA. Long-term trandolapril treatment is associated with reduced aortic stiffness: the prevention of events with angiotensin-converting enzyme inhibition hemodynamic substudy. Hypertension. 2007 Jun 1;49(6):1271-7.
- Rahman S, Ismail AA, Ismail SB, Naing NN, Abdul Rahman AR. Effect of rosiglitazone/ramipril on preclinical vasculopathy in newly diagnosed, untreated diabetes and IGT patients: 1-year randomised, double-blind, placebo-controlled study. European journal of clinical pharmacology. 2007 Aug;63:733-41.
- Rehman A, Ismail SB, Naing L, Roshan TM, Rahman AR. Reduction in arterial stiffness with angiotensin II antagonism and converting enzyme inhibition: a comparative study among Malay hypertensive subjects with a known genetic profile. American journal of hypertension. 2007 Feb 1;20(2):184-9.
- Ahimastos AA, Dart AM, Lawler A, Blombery PA, Kingwell BA. Reduced arterial stiffness may contribute to angiotensin-converting enzyme inhibitor induced improvements in walking time in peripheral arterial disease patients. Journal of hypertension. 2008 May 1;26(5):1037-42.
- Dhakam Z, McEniery CM, Burton T, Brown MJ, Wilkinson IB. A comparison of atenolol and nebivolol in isolated systolic hypertension. Journal of hypertension. 2008 Feb 1;26(2):351-6.
- Ferguson JM, Minas J, Siapantas S, Komesaroff PA, Sudhir K. Effects of a fixed- dose ACE inhibitor-diuretic combination on ambulatory blood pressure and arterial properties in isolated systolic hypertension. Journal of cardiovascular pharmacology. 2008 Jun 1;51(6):590-5.
- Golovanova ED. Vascular remodeling and heart rate variability in different antihypertensive therapies. Rational Pharmacotherapy in Cardiology.2008;4(1):62-6.
- Ishii H, Tsukada T, Yoshida M. Angiotensin II type-I receptor blocker, candesartan, improves brachial-ankle pulse wave velocity independent of its blood pressure lowering effects in type 2 diabetes patients. Internal Medicine. 2008;47(23):2013-8.
- Karalliedde J, Smith A, DeAngelis L, Mirenda V, Kandra A, Botha J, Ferber P, Viberti G. Valsartan improves arterial stiffness in type 2 diabetes independently of blood pressure lowering. Hypertension. 2008 Jun 1;51(6):1617-23.
- Kosch M, Levers A, Lang D, Bartels V, Rahn KH, Pavenstädt H, Hausberg M. A randomized, double-blind study of valsartan versus metoprolol on arterial distensibility and endothelial function in essential hypertension. Nephrology Dialysis Transplantation. 2008 Jul 1;23(7):2280-5.
- Morimoto S, Maki K, Aota Y, Sakuma T, Iwasaka T. Beneficial effects of combination therapy with angiotensin II receptor blocker and angiotensin- converting enzyme inhibitor on vascular endothelial function. Hypertension Research. 2008 Aug;31(8):1603-10.
- Schneider MP, Delles C, Klingbeil AU, Ludwig M, Kolloch RE, Krekler M, Stumpe KO, Schmieder RE. Effect of angiotensin receptor blockade on central haemodynamics in essential hypertension: results of a randomised trial. Journal of the Renin-Angiotensin-Aldosterone System. 2008 Mar;9(1):49-56.
- Mackenzie IS, McEniery CM, Dhakam Z, Brown MJ, Cockcroft JR, Wilkinson IB. Comparison of the effects of antihypertensive agents on central blood pressure and arterial stiffness in isolated systolic hypertension. Hypertension. 2009 Aug 1;54(2):409-13.
- Matsui Y, Eguchi K, O'Rourke MF, Ishikawa J, Miyashita H, Shimada K, Kario

K. Differential effects between a calcium channel blocker and a diuretic when used in combination with angiotensin II receptor blocker on central aortic pressure in hypertensive patients. Hypertension. 2009 Oct 1;54(4):716-23.

- Musikhina NY, Gapon LI, Makhneva EA, Belikova EA, Petelina TY, Todosiychuk VV, Musikhina NA, Musikhina NA, Yuferova OV, Yuferova OV, Gapon LI. Structural-functional properties of the vascular wall in hypertensive patients with coronary heart disease: effects of felodipin and perindopril. Terapevticheskii arkhiv. 2009 Sep 15;81(9):13-6.
- Li Y, Ma SM, Du M, Chu WW, Cheng XM. Perindopril, amlodipine and telmisartan improve arterial stiffness in patients with hypertension. Zhonghua Xin Xue Guan Bing Za Zhi. 2009 Oct 1;37(10):908-12.
- Moltzer E, Raso FU, Karamermer Y, Boersma E, Webb GD, Simoons ML, Danser AJ, Van den Meiracker AH, Roos-Hesselink JW. Comparison of candesartan versus metoprolol for treatment of systemic hypertension after repaired aortic coarctation. The American journal of cardiology. 2010 Jan 15;105(2):217-22.
- Tomiyama H, Yoshida M, Yamada J, Matsumoto C, Odaira M, Shiina K, Yamashina A. Arterial-cardiac destiffening following long-term antihypertensive treatment. American journal of hypertension. 2011 Oct 1;24(10):1080-6.
- Hayoz D, Zappe DH, Meyer MA, Baek I, Kandra A, Joly MP, Mazzolai L, Haesler E, Periard D. Changes in aortic pulse wave velocity in hypertensive postmenopausal women: comparison between a calcium channel blocker vs angiotensin receptor blocker regimen. The Journal of Clinical Hypertension. 2012 Nov;14(11):773-8.
- Matsui Y, O'Rourke MF, Hoshide S, Ishikawa J, Shimada K, Kario K. Combined effect of angiotensin II receptor blocker and either a calcium channel blocker or diuretic on day-by-day variability of home blood pressure: the Japan Combined Treatment With Olmesartan and a Calcium-Channel Blocker Versus Olmesartan and Diuretics Randomized Efficacy Study. Hypertension. 2012 Jun;59(6):1132- 8.
- Nedogoda SV, Ledyaeva AA, Chumachek EV, Tsoma BB, Mazina GG, Salasyuk AS, Barykina IN. Comparative efficacy of perindopril and losartan in patients with arterial hypertension and obesity. Russian Cardiological Journal. 2012(1):63-9.
- Spanos G, Kalaitzidis R, Karasavvidou D, Pappas K, Siamopoulos KC. Efficacy of aliskiren and valsartan in hypertensive patients with albuminuria: a randomized parallel-group study. Journal of the Renin-Angiotensin-Aldosterone System. 2013 Dec;14(4):315-21.
- Tishina EV, Mychka VB, Saidova MA. Combined antihypertensive therapy based on moxonidine in patients with metabolic syndrome. Cardiovascular therapy and prophylaxis. 2012;11(3):36-46.
- Virdis A, Ghiadoni L, Qasem AA, Lorenzini G, Duranti E, Cartoni G, Bruno RM, Bernini G, Taddei S. Effect of aliskiren treatment on endothelium-dependent vasodilation and aortic stiffness in essential hypertensive patients. European heart journal. 2012 Jun 1;33(12):1530-8.
- Vitale C, Marazzi G, Iellamo F, Spoletini I, Dall'Armi V, Fini M, Volterrani M. Effects of nebivolol or irbesartan in combination with hydrochlorothiazide on vascular functions in newly-diagnosed hypertensive patients: the NINFE (Nebivololo, Irbesartan Nella Funzione Endoteliale) study. International journal of cardiology. 2012 Mar 8;155(2):279-84.
- Agnoletti D, Zhang Y, Borghi C, Blacher J, Safar ME. Effects of antihypertensive drugs on central blood pressure in humans: a preliminary observation. American journal of hypertension. 2013 Aug 1;26(8):1045-52.
- Hare JL, Sharman JE, Leano R, Jenkins C, Wright L, Marwick TH. Impact of spironolactone on vascular, myocardial, and functional parameters in untreated patients with a hypertensive response to exercise. Am J Hypertens. 2013 May;26(5):691-9.
- Sang-Hyun I, Hui-Kyung J, Shung CC, Do-Sun L, Kee-Sik K, Dong-Ju C, Jong- Won H. Benidipine has effects similar to losartan on the central blood pressure and arterial stiffness in mild to moderate essential hypertension. Chinese Medical Journal. 2013 Jun 5;126(11):2021-8.
- Koumaras C, Tziomalos K, Stavrinou E, Katsiki N, Athyros VG, Mikhailidis DP, Karagiannis A. Effects of renin-angiotensin-aldosterone system inhibitors and beta-blockers on markers of arterial stiffness. Journal of the American Society of Hypertension. 2014 Feb 1;8(2):74-82.
- Nedogoda SV, Chumachek EV, Ledyaeva AA, Tsoma AV, Salasyuk AS. Comparative effectiveness of fixed-dose combinations of lisinopril/amlodipine and enalapril/hydrochlorothiazide. Cardiovascular Therapy and Prevention. 2013 Apr 20;12(2):25-9.
- Nedogoda SV, Ledyaeva AA, Chumachok EV, Tsoma VV, Mazina G, Salasyuk AS, Barykina IN. Randomized trial of perindopril, enalapril, losartan and telmisartan in overweight or obese patients with hypertension. Clinical drug investigation. 2013 Aug;33:553-61.
- Radchenko GD, Sirenko YM, Kushnir SM, Torbas OO, Dobrokhod AS. Comparative effectiveness of a fixed-dose combination of losartan+ HCTZ versus bisoprolol+ HCTZ in patients with moderate-to-severe hypertension: results of the 6-month ELIZA trial. Vascular Health and Risk Management. 2013 Sep 27:535-49.
- Zhou WJ, Wang RY, Li Y, Chen DR, Chen EZ, Zhu DL, Gao PJ. A randomized controlled study on the effects of bisoprolol and atenolol on sympathetic nervous activity and central aortic pressure in patients with essential hypertension. PLoS One. 2013 Sep 10;8(9):e72102.
- Kim EJ, Song WH, Lee JU, Shin MS, Lee S, Kim BO, Hong KS, Han SW, Park CG, Seo HS. Efficacy of losartan and carvedilol on central hemodynamics in hypertensives: a prospective, randomized, open, blinded end point, multicenter study. Hypertension Research. 2014 Jan;37(1):50-6.
- Pozzobon CR, Gismondi RA, Bedirian R, Ladeira MC, Neves MF, Oigman W. Functional vascular study in hypertensive subjects with type 2 diabetes using losartan or amlodipine. Arquivos Brasileiros de Cardiologia. 2014 Jul 9;103:51- 9.
- Semenkin AA, Zhenatov AB, Zhivilova LA, Nechaeva GI, Pritykina TV, Chindareva OI, Stroeva TV. Direct comparison of endothelial and metabolic effects of perindopril combination with indapamide retard or hydrochlorothiazide. Kardiologiia. 2014 Jan 1;54(11):25-9.
- Sumbria M, Negi PC, Sahai AK, Kaundal PK. To compare the effect of telmisartan with metoprolol on arterial stiffness in hypertension: prospective randomized parallel group trial. indian heart journal. 2014 Jul 1;66(4):415-21.
- Hayek SS, Poole JC, Neuman R, Morris AA, Khayata M, Kavtaradze N, Topel ML, Binongo JG, Li Q, Jones DP, Waller EK. Differential effects of nebivolol and metoprolol on arterial stiffness, circulating progenitor cells, and oxidative stress. Journal of the American Society of Hypertension. 2015 Mar 1;9(3):206- 13.
- Pizoń T, Rajzer M, Wojciechowska W, Rojek M, Kameczura T, Jurczyszyn A, Czarnecka D. The influence of antihypertensive treatment on arterial stiffness,

shear stress and activity of chosen matrix metalloproteinases. Przeglad Lekarski. 2015 Jan 1;72(2):53-9.

- Posadzy-Malaczynska A, Rajpold K, Woznicka-Leskiewicz L, Marcinkowska J. Hemodynamic and metabolic effects of estrogen plus progestin therapy in hypertensive postmenopausal women treated with an ACE-inhibitor or a diuretic. Clinical research in cardiology. 2015 Jan;104:38-50.
- Rajzer M, Wojciechowska W, Kameczura T, Olszanecka A, Fedak D, Terlecki M, Kawecka-Jaszcz K, Czarnecka D. The effect of antihypertensive treatment on arterial stiffness and serum concentration of selected matrix metalloproteinases. Archives of Medical Science. 2017 Jun 8;13(4):760-70.
- Redon J, Pichler G. Comparative study of the efficacy of olmesartan/amlodipine vs. perindopril/amlodipine in peripheral and central blood pressure parameters after missed dose in type 2 diabetes. American Journal of Hypertension. 2016 Sep 1;29(9):1055-62.
- Tsioufis C, Dimitriadis K, Mantzouranis E, Mani I, Tousoulis D. Differential effects of lercanidipine/enalapril versus amlodipine/enalapril and hydrochlorothiazide/enalapril on target organ damage and sympathetic activation in non-obese essential hypertensive subjects. Current Medical Research and Opinion. 2016 Sep 30;32(sup2):35-41.
- Nedogoda SV, Konradi AO, Zvartau NE, Chumachek EV, Ledyaeva AA, Tsoma VV, Salasyuk AS, Judina JS, Smirnova VO, Khripaeva VJ, Palashkin RV. Optimization of BP control and arterial elasticity with fixed combination therapy perindopril and amlodipine in patients with arterial hypertension and increased pulse wave velocity. Kardiologiia. 2017 Mar 1;57(3):31-8.
- Williams B, Cockcroft JR, Kario K, Zappe DH, Brunel PC, Wang Q, Guo W. Effects of sacubitril/valsartan versus olmesartan on central hemodynamics in the elderly with systolic hypertension: the PARAMETER study. Hypertension. 2017 Mar;69(3):411-20.
- Accetto R, Widimsky J Jr, Vincelj J, Sirenko Y, Yevgenyevna IC, Barbic Zagar

B. The efficacy and safety of valsartan and a combination of valsartan and hydrochlorothiazide in the treatment of patients with mild to moderate arterial hypertension: a subgroup analysis of the effect of valsartan and its combination with hydrochlorothiazide on pulse wave velocity and central blood pressure. Kardiol Pol. 2018;76(2):328-337.

- Jekell A, Kahan T. The usefulness of a single arm cuff oscillometric method (Arteriograph) to assess changes in central aortic blood pressure and arterial stiffness by antihypertensive treatment: results from the Doxazosin-Ramipril Study. Blood Pressure. 2018 Mar 4;27(2):88-98.
- Dudinskaya E, Tkacheva O, Bazaeva E, Matchekhina L, Eruslanova K, Sharashkina N, Kotovskaya Y, Larina V. Influence of Moxonidine and Bisoprolol on Morphofunctional Condition of Arterial Wall and Telomerase Activity in Postmenopausal Women with Arterial Hypertension and Osteopenia. The Results

from a Moscow Randomized Study. Cardiovascular Drugs and Therapy. 2021 Sep 15:1-9.

- Shishko VI, Karpovich OA, Kornelyuk DG, Shulga EV, Larionova IN, Zhmakina EN. Comparative efficacy of chlorthalidone and hydrochlorthiazide in the combined treatment of patients with arterial hypertension grade I-II with abdominal obesity. Cardiology in Belarus, 2022 14(1):116-124.
